# Bent N‐Heterocyclic Allenes From a Well‐Defined Titanium Vinylidene Complex

**DOI:** 10.1002/anie.5744116

**Published:** 2026-04-02

**Authors:** Bastiaan Kooij, Damien W. Chen, Rosario Scopelliti, Farzaneh Fadaei‐Tirani, Kay Severin

**Affiliations:** ^1^ Institut des Sciences et Ingénierie Chimiques Ecole Polytechnique Fédérale de Lausanne (EPFL) Lausanne Switzerland

**Keywords:** allene, push‐pull systems, titanium, transition metal, vinylidene complex

## Abstract

Allenes typically display a linear geometry. A notable exception occurs in allenes bearing two electron‐donating N‐heterocyclic capping groups, which can display a strongly bent C═C═C unit. These push‐push allenes, known as carbodicarbenes, are powerful carbon‐donor ligands with broad utility in chemistry. Computational studies have suggested that push‐pull allenes, featuring both an electron‐donating and withdrawing capping group, can also adopt a bent geometry. However, experimental confirmation has so far been lacking. Herein, we report allenes featuring an N‐heterocyclic capping group on one side, and either diarylmethylidene or fluorenylidene capping groups on the other. The synthesis of these allenes was accomplished via a titanium vinylidene complex. The latter could be isolated and analyzed by X‐ray diffraction, and it represents the first structurally characterized Ti vinylidene complex. The push‐pull allenes with fluorenylidene capping groups revealed markedly bent C═C═C units (α_c‐c‐c_ < 140°), whereas the less polarized diarylmethylidene analogues were found to display a more linear geometry. The geometric differences correlate with a divergent reactivity upon thermal activation. Both types of N‐heterocyclic allenes can be used as carbon‐donor ligands for transition metal complexes.

## Graphical abstract

The syntheses and the structures of N‐heterocyclic allenes are reported. Allenes bearing fluorenylidene capping groups show markedly bent C═C═C units, a carbene‐like reactivity, and they can act as potent C‐donor ligands for metal complexes.

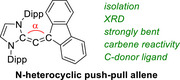

## 1 Introduction

**FIGURE 1:**
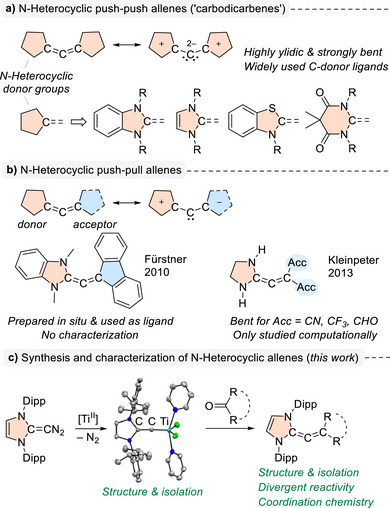
(a) N‐Heterocyclic push‐push allenes are highly ylidic compounds with a strongly bent geometry. (b) Computational results suggest that N‐heterocyclic push–pull allenes would also adopt an atypical, non‐linear geometry, but the isolation of such compounds has remained elusive. (c) Herein, we describe the synthesis, the structures, and the reactivity of bent N‐heterocyclic allenes using a well‐defined Ti vinylidene complex as a key intermediate. Dipp = 2,6‐diisopropylphenyl.

Allenes of the general formula R_2_C═C═CR_2_ (R = hydrogen, alkyl, aryl) possess two orthogonal π systems, resulting in a linear geometry [1]. In 2008, Bertrand and co‐workers provided experimental evidence that non‐cyclic allenes can adopt a strongly bent geometry [2]. They synthesized an allene capped by two 1,3‐dimethylbenzimidazolylidene groups, termed a “carbodicarbene.” This N‐heterocyclic allene features a C═C═C angle of 134.8°, as determined by a crystallographic analysis [2]. The bending is the result of a strongly ylidic character induced by the electron‐donating heterocyclic capping groups (Figure 1a).

Following the seminal report by Bertrand and co‐workers, carbodicarbenes have been studied extensively [3, 4, 5, 6, 7]. Structural variations were explored (Figure 1a), including carbodicarbenes with different caping groups [8, 9, 10, 11, 12], and the reactivity of these unusual allenes was investigated. A key characteristic is the Lewis basicity of the central carbon atoms, enabling the use of carbodicarbenes as C‐donor ligands in transition metal [6] and main‐group element chemistry [7].

Allenes with a push–pull substitution pattern can display carbene‐like character [13, 14], and those bearing N‐heterocyclic donor groups should be particularly well suited to reveal such behavior. However, only limited information about such allenes is available so far. In 2010, the Fürstner group reported the use of an allene with a 1,3‐dimethylbenzimidazolylidene donor group and a fluorenylidene acceptor group (Figure 1b) as a ligand for Au(I) and Rh(I) complexes [15]. The allene itself was prepared in situ at a low temperature (–78 –°C), and neither a spectroscopic nor a structural characterization was provided. A subsequent computational analysis suggested that the allene should have a bent geometry [16]. Additional computational studies of push‐pull allenes at the DFT level were performed by Kleinpeter and co‐workers [17]. Most allenes were found to adopt the normal linear geometry, with the exception of allenes with imidazoline‐2‐ylidene donor groups, for which a bent structure was observed (Figure 1b). Taken together, the computational results indicate that N‐heterocyclic push‐pull allenes are promising candidates in the search for allenes with atypical, non‐linear geometries.

Below, we describe allenes with an electron‐donating 1,3‐bis(2,6‐diisopropylphenyl)imidazol‐2‐ylidene (IDipp) capping group on one side, and either diarylmethylidene or fluorenylidene capping groups on the other. The synthesis of these allenes was achieved by combining the corresponding ketones with a titanium vinylidene complex (Figure 1c). The latter could be isolated and crystallized, and it represents the first structurally characterized Ti vinylidene complex. The allenes bearing fluorenylidene groups show markedly bent C═C═C units (α_c‐c‐c_ < 140°), whereas the less polarized diarylmethylidene analogs exhibited a more linear geometry. The geometric differences correlate with a divergent reactivity upon thermal activation. Moreover, we demonstrate that N‐heterocyclic allenes can serve as C‐donor ligands for transition metal complexes.

## 2 Results and Discussion

The combination of titanium alkylidene complexes with carbonyl compounds represents a versatile route for the synthesis of olefins [18, 19]. Typically, the reactive alkylidene complexes are generated in situ from more stable precursors, such as the Tebbe reagent. In a related fashion, it is possible to use Ti reagents for the synthesis of allenes [20, 21, 22, 23]. These transformations are believed to proceed via vinylidene complexes of type L*_n_* Ti(═C═CR^1^R^2^), but the isolation of Ti complexes bearing terminal vinylidene ligands has remained elusive [24, 25].

We have recently shown that the N‐heterocyclic diazoolefin (IDipp)CN_2_ (**1**) [26, 27, 28] can be used to prepare vanadium and iridium complexes with the vinylidene ligand (IDipp)C [29, 30, 31]. Encouraged by these results, we have explored whether we could prepare a Ti vinylidene complex from diazoolefin **1**. As a reaction partner, we used the Ti(II) complex TiCl_2_(Py)_4_ (Py = pyridine) [32]. The choice of a Ti(II) complex was motivated by reports about the formation of Ti alkylidene complexes in reactions of Ti(II) synthons with diazoalkanes [33, 34, 35].

**SCHEME 1:**
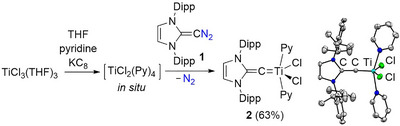
Synthesis of complex **2**, along with a depiction of the molecular structure of **2** in the solid state, as determined by single‐crystal XRD. Only one of the two independent molecules is shown. The thermal ellipsoids are at 50% probability, and hydrogen atoms are not shown.

When TiCl_2_(Py)_4_ was combined with diazoolefin **1** in THF, we observed the formation of complex **2**. Conveniently, TiCl_2_(Py)_4_ can be prepared in situ by reduction of TiCl_3_(THF)_3_ with KC_8_ in the presence of pyridine. This one‐step procedure allows isolating complex **2** in 63% yield (Scheme 1).

Although highly sensitive to air and moisture, complex **2** could be stored as a solid at –40°C for several weeks, and it remained stable in benzene at room temperature for multiple hours. The {^1^H}^13^C NMR signal of the metalated carbon resonates at 271.2 ppm (C_6_D_6_). This value is in the range found for Ti alkylidene complexes [36, 37].

Complex **2** was also characterized by single‐crystal X‐ray diffraction (XRD). Two independent molecules with comparable structural parameters were found in the unit cell. The complexes display a distorted square pyramidal coordination geometry, with two pyridine ligands, two chloro ligands, and the vinylidene ligand (Scheme 1). The Ti–C–C group in both complexes is nearly linear, with bond angles of 178.3(3)° and 177.3(3)°. The Ti–C bond lengths of 1.799(3) Å and 1.786(3) Å are short compared to what was reported for Ti alkylidene complexes (∼1.9 Å) [36, 37]. Similarly short Ti–C bonds, 1.784(3) and 1.785(3) Å, were found for a Ti complex with a C(PPh_2_Me) ligand [38]. This compound was described as an alkylidyne complex with ylene character. Accordingly, the vinylidene ligand in **2** should display some alkylidyne character. However, it is worth noting that the computed Ti–C bond length for an authentic Schrock alkylidyne with a Ti≡C–*^t^* Bu ligand, 1.743 Å, is even shorter than what was found for 2 [38]. The TiC–C bond lengths observed for complex **2**, 1.388(4) Å and 1.393(4) Å, suggest significant double bond character, underlining the description as a vinylidene complex.

**SCHEME 2:**
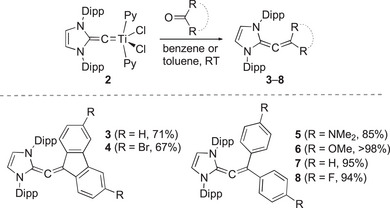
Synthesis of N‐heterocyclic allenes **3**–**8**.

With a convenient synthesis of complex **2** at hand, we investigated whether it could be used for the synthesis of allenes. When **2** was combined with fluorenone, the targeted N‐heterocyclic allene **3** was indeed formed (Scheme 2). Allene **3** could be obtained in 71% yield after extraction with pentane. Alternatively, it is possible to prepare complex **3** directly from TiCl_3_(THF)_3_, KC_8_, and diazoolefin **1**, resulting in a lower yield of 39%. Bromo substituents were expected to enhance the acceptor capabilities of the fluorenylidene group. Therefore, we synthesized the dibromo derivative **4** using 3,6‐dibromofluorenone and complex **2** as the starting materials (Scheme 2).

For comparison, we also prepared allenes with diarylmethylidene capping groups. These compounds (**5**–**8**) were synthesized analogously by coupling complex **2** with benzophenone or its derivatives (Scheme 2).

**FIGURE 2:**
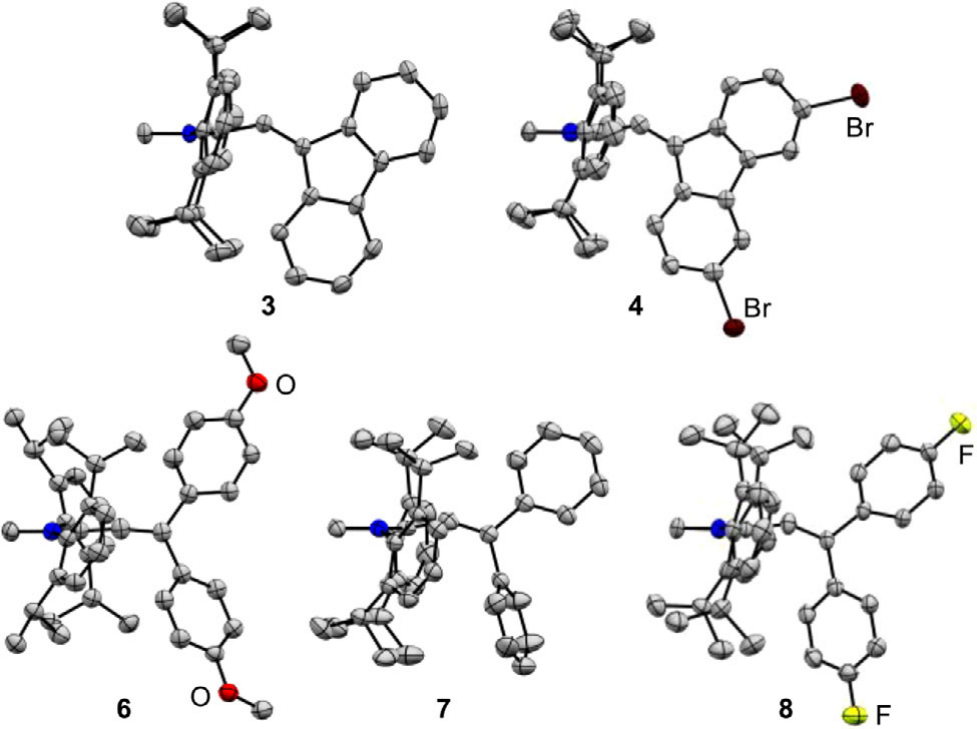
Molecular structures of the allenes **3, 4, 6, 7,** and **8** in the crystal. The thermal ellipsoids are at 50% probability for compounds **3, 4, 7,** and **8**, and at 30% for compound **6**. Hydrogen atoms are not shown.

In solution, allenes **3**–**8** displayed a variable stability, as discussed in more detail further below. Nonetheless, the allenes could be characterized by NMR spectroscopy and mass spectrometry. Importantly, we were able to grow single crystals of all new allenes, except compound **5**. Graphic representations of the solid‐state structures are shown in Figure 2, and key structural parameters are listed in Table 1.

**TABLE 1:** Selected structural and spectroscopic parameters for the allenes **3**, **4**, **6**, **7**, and **8**.

Allene	*α*_(C1–C2–C3)_ (°)	*d*_(C1–C2)_ (Å)	*d*_(C2–C3)_ (Å)	*δ*_(C2)_ (ppm)
**3**	138.0(1)	1.371(2)	1.331(2)	204
**4**	138.7(4)	1.384(5)	1.323(5)	216
**6**	172.0(5)	1.313(6)	1.326(6)	200
**7**	152.4(3)	1.340(4)	1.324(4)	204
**8**	149.2(2)	1.358(3)	1.322(3)	204

The two fluorenone‐based allenes, **3** and **4**, have markedly bent geometries, with bent angles of 138.0(1)° and 138.7(4)°, respectively (Table 1). These values are in the range found for the most bent carbodicarbenes [2, 8, 9]. In both allenes, the C1–C2 bonds are substantially longer than the C2–C3 bonds (Table 1). The data imply a partial single‐bond character for the C1–C2 bond, whereas the C2–C3 bond lengths are in the range found for allenes with fluorenylidene capping groups [15, 39, 40].

The allenes **6**–**8** with diarylmethylidene capping groups are overall less bent than the fluorenone‐based allenes **3** and **4**. While pronounced bending is still observed for **7** (152.4(3)°) and **8** (149.2(2)°), allene **6** is nearly linear (172.0(5)°). The degree of bending correlates with the length of the C1–C2 bond, with the shortest bond observed for allene **6**.

It is interesting to note that the chemical shift of the ^13^C NMR signal of the central carbon atom C2 is found in the range of 200 ppm (Table 1). Similar values are found for non‐polarized allenes of type R_2_C═C═CR_2_ (R = hydrogen, alkyl, aryl) [41]. In carbodicarbenes, on the other hand, these signals are shifted substantially toward higher fields (*δ* = 110–159 ppm) [2, 8, 9, 10].

**SCHEME 3:**
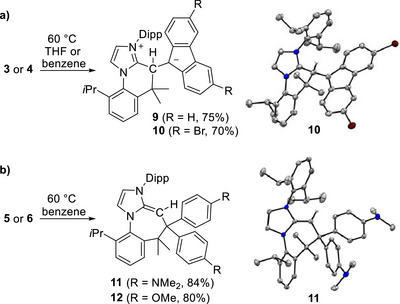
(a) Isomerization of **3** and **4** into the zwitterionic compounds **9** and **10**, along with a depiction of the molecular structure of **10** in the crystal, as determined by single‐crystal XRD. Only one of the two independent molecules is shown. The thermal ellipsoids are at 20%, and hydrogen atoms are not shown. The yields refer to reactions performed in THF. (b) Isomerization of **5** and **6** into the N‐heterocyclic olefins **11** and **12**, along with a depiction of the molecular structure of **11** in the crystal, as determined by single‐crystal XRD. The thermal ellipsoids are at 50%, and hydrogen atoms are not shown.

While performing NMR analyses, we noticed that the allenes **3**–**6** converted slowly into new products (**9**–**12**). This conversion could be completed by heating solutions of the compounds in THF or benzene at 60°C for 2–7 h (Scheme 3). In the case of **3** and **4**, the thermal rearrangement was visible to the “naked eye,” with the colors of the solutions turning from violet to dark blue (**3**→**9**) or from light pink to dark red (**4**→**10**). In the case of **5** and **6**, the solutions were only slightly colored (yellow/orange), and minor changes in color were detected upon thermal rearrangement.

NMR spectroscopic analyses of the products, along with XRD analyses of **10** and **11**, provided evidence for two distinct reaction pathways. The rearrangement of the allenes **3** and **4** led to the formation of the zwitterionic compounds **9** and **10**. This type of transformation underscores the carbene character of the push‐pull allenes **3** and **4** (Figure 1b), with the products originating from an intramolecular C–H activation of one isopropyl side chain at the central carbon atom of the allene [42, 43, 44]. The kinetics of the two reactions are in line with the assumption that the push‐pull polarization is crucial for the rearrangement, with allene **4** reacting faster than allene **3** (the bromo‐substituents accentuate the acceptor character of the fluorenylidene group).

The rearranged products **11** and **12** also show a new C–C bond involving a former isopropyl group, but the new bond has led to the formation of a 7‐membered ring. A plausible mechanism for this transformation consists of an initial deprotonation of the isopropyl group by the basic central carbon atom, followed by an intramolecular coupling of the resulting benzyl anion with the terminal carbon atom of the former allene. The reactivity implies that allenes **5** and **6** have push‐push character, similar to carbodicarbenes. The push‐push character is expected to be stronger for the dimethylamino‐substituted allene **5** when compared to the methoxy‐substituted allene **6**. Accordingly, the rearrangement of **5** into **11** was faster than the rearrangement of **6** into **12**.

In order to examine if the distinct reactivity can be traced back to differences in the electronic structure, we have performed quantum‐chemical analyses of the allenes **3** and **6** at the TPSSh/def2‐TZVP level of theory (for details, see the Supporting Information, Section 5) [45, 46]. By visual inspection of the Kohn–Sham orbitals, we noticed that the HOMO and HOMO–1 of **6** correspond to two orthogonal π systems centered on the C═C═C bonds, as in normal, linear allenes (Figure S89) [1]. In contrast, the HOMO of **3** shows a noticeable distortion, resembling a *σ*‐type lone pair. NBO analysis did not assign a lone pair on the central carbon of either allene, consistent with delocalization of the electrons within the π system. However, an electron localization function (ELF) [47] basin analysis revealed a monosynaptic valence basin at the central carbon of **3**, whose population, position, and size are consistent with lone‐pair‐like electron density (Figure S89) [48, 49]. No monosynaptic valence basins were found on the central carbon of **6**. These results show that the central carbon of **3** has a partial lone pair character that is absent in **6**.

**SCHEME 4:**
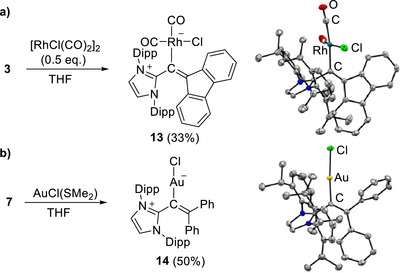
(a) Synthesis of complex **13**, along with a depiction of the molecular structure of **13** in the solid state, as determined by single‐crystal XRD. The thermal ellipsoids are at 50% probability. Hydrogen atoms are not shown. (b) Synthesis of complex **14**, along with a depiction of the molecular structure of **14** in the solid state, as determined by single‐crystal XRD. The thermal ellipsoids are at 50% probability. Hydrogen atoms are not shown.

Carbodicarbenes are extensively used as ligands in transition metal chemistry [6]. We were interested in exploring whether the herein reported N‐heterocyclic allenes could also function as C‐donor ligands when paired with transition metals. Combining the push‐pull allene **3** with [RhCl(CO)_2_]_2_ (0.5 equiv.) led to the formation of metal complex **13**, which could be isolated in 33% yield after workup (Scheme 4). The formation of complex **13** was confirmed by NMR spectroscopy, mass spectrometry, FT‐IR, and single‐crystal XRD analysis. The latter revealed *η* ^1^‐coordination of the RhCl(CO)_2_ fragment to the central carbon atom, and elongation of the C_imidazole_–C bond (1.466(2) Å), while the C–C_fluorenylidene_ bond length increased only slightly (1.355(2) Å). Thus, the metalated allene in complex **13** can be described as a vinyl ligand with an imidazolium substituent. The C–Rh bond length of 2.093(2) Å is consistent with values observed in structurally related carbodicarbene complexes [2, 8].

The infrared bands of the CO groups of **13** are of special interest because they reflect the donor capabilities of ligand **3**. The FT‐IR spectrum of complex **13** shows bands at 2051 and 1979 cm^−1^. These values are nearly identical to those observed for the first reported carbodicarbene complex by Bertrand and co‐workers (2052 and 1976 cm^−1^) [2]. This similarity suggests that push‐pull allene **3** is also a very strong C‐donor ligand.

We have also investigated the coordination ability of allene **7**, which features a less bent geometry when compared to **3**. When allene **7** was combined with AuCl(SMe_2_), the Au^I^ complex **14** could be isolated in 50% yield (Scheme 4). The product was analyzed by NMR spectroscopy, mass spectrometry, and single‐crystal XRD. Similar to complex **13**, ligand **7** binds to the central carbon atom, and strong elongation of the C_imidazole_–C bond is observed (1.446(4) Å), with only minor lengthening of the C–CPh_2_ bond (1.364(4) Å). The C–Au bond length in **14**, 2.021(2) Å, falls within the range observed in carbodicarbene‐gold complexes [10].

## 3 Conclusion

Most allenes have a linear geometry, and deviations from this geometry are rare and conceptually significant. We have reported a series of allenes featuring an electron‐donating N‐heterocyclic capping group on one side, and either diarylmethylidene or fluorenylidene capping groups on the other side. For the synthesis of these allenes, we have employed a structurally characterized titanium complex with a terminal vinylidene ligand. The N‐heterocyclic allenes **3** and **4** with fluorenylidene capping groups have strongly bent C═C═C units, as evidenced by XRD analyses. These results represent the first experimental evidence that a push‐pull substitution pattern in allenes can give rise to a similar degree of bending as observed for carbodicarbenes. In contrast to carbodicarbenes, the allenes **3** and **4** display a carbene‐like reactivity upon thermal activation. With the synthesis of the Rh^I^ and Au^I^ complexes **13** and **14**, we have demonstrated that N‐heterocyclic allenes can serve as C‐donor ligands for transition metals, and future applications in catalysis can be envisioned.

## Conflicts of Interest

The authors declare no conflicts of interest.

## Supporting information




**Supporting File 1**: anie72099‐sup‐0001‐SuppMat.pdf.

## Data Availability

The data that support the findings of this study are available in the Supporting Information of this article.
