# Supplementary material for: Bent N‐Heterocyclic Allenes From a Well‐Defined Titanium Vinylidene Complex
Source: Angew Chem Int Ed Engl. 2026 Apr 2;65(20):e5744116. doi: 10.1002/anie.5744116 (PMC13159420; doi:10.1002/anie.5744116)
Supplement: Supplementary file 1 — Supporting File 1: anie72099‐sup‐0001‐SuppMat.pdf. [file ANIE-65-e5744116-s001.pdf]

Supporting Information

Bent N-Heterocyclic Allenes from a Well-Defined Titanium Vinylidene Complex

Bastiaan Kooij, Damien W. Chen, Rosario Scopelliti, Farzaneh Fadaei-Tirani, and Kay Severin*

Institute of Chemical Sciences and Engineering, École Polytechnique Fédérale de Lausanne (EPFL), CH-1015 Lausanne, Switzerland

e-mail: kay.severin@epfl.ch

Contents

1. General 2

2. Syntheses 3

3. NMR Data 22

4. IR Data 58

5. UV/Vis Data 59

4. XRD Analyses 63

5. Quantum Chemical Calculations 93

6. References 98

1. General

Unless stated otherwise, all reactions were performed under an atmosphere of dry dinitrogen using an MBraun glove box (O_2 and H_2O < 0.5 ppm). Solvents were taken from an Innovative Technology solvent purification system, and were stored under 4 Å molecular sieves for a minimum of two days prior to use. All glassware was dried in an oven at 170 °C for a minimum of one day prior to use. Celite and glass wool filters were dried in an oven at 170 °C for a minimum of one day before use. Diazoolefin **1** and KC_8 were prepared according to the respective published procedures.[1,2] The commercially obtained $TiCl_3(THF)_3$ complex was purified by washing with dry THF (3x) before use. Liquid ketones were degassed and dried under 4 Å molecular sieves for a minimum of two days, and solid ketones were placed under a vacuum for a minimum of one day prior to use. If not stated otherwise, all other reagents were obtained from commercial sources and used without further purification.

The NMR spectra were measured on a Bruker Avance DPX-400 (^{1}H: 400 MHz), Bruker Avance NEO-500 (^{1}H: 500 MHz), Bruker Avance IIIHD-600 (^{1}H: 600 MHz), or Bruker Avance II (^{1}H: 800 MHz) spectrometer with BBFOz ATMA probe. Chemical shifts are given in parts per million (ppm) relative to their solvent signals [C_6D_6, 7.16 (^{1}H-NMR) and 128.06 (^{13}C-NMR), d_8-THF, 1.72 and 3.58 (^{1}H-NMR) 25.31 and 67.21 (^{13}C-NMR), CD_2Cl_2, 5.32 (^{1}H-NMR) and 53.84 (^{13}C-NMR)]. The NMR spectra were measured at 298 K, unless stated otherwise.

UV-vis data were recorded on a Cary 60 Spectrometer (Agilent Technologies). FT-IR spectra were acquired on a Perkin-Elmer Spectrum-One instrument with diamond-anvil configuration. Mass spectrometry were performed on a LTQ Orbitrap FTMS instrument (LTQ Orbitrap Elite FTMS, Thermo Scientific) operated in the positive mode coupled with a robotic chip-based nano-ESI source (TriVersa Nanomate, Advion Biosciences). A standard data acquisition and instrument control system was utilized (Thermo Scientific) whereas the ion source was controlled by Chipsoft 8.3.1 software (Advion BioScience). Samples were loaded onto a 96-well plate within an injection volume of 5 µl. The experimental conditions for the ionization voltage were +1.4 kV and the gas pressure was set at 0.30 psi. The temperature of ion transfer capillary was 200 °C. FTMS spectra were obtained in the 100-1000 *m/z* range in the reduced profile mode with a resolution set to 120,000. In all spectra, 1 microscan was acquired with a maximum injection time value of 1000 ms.

2. Syntheses

Complex $TiCl_2Py_4$ was synthesized using a modified version of the procedure reported by J. Telser et al.[3]

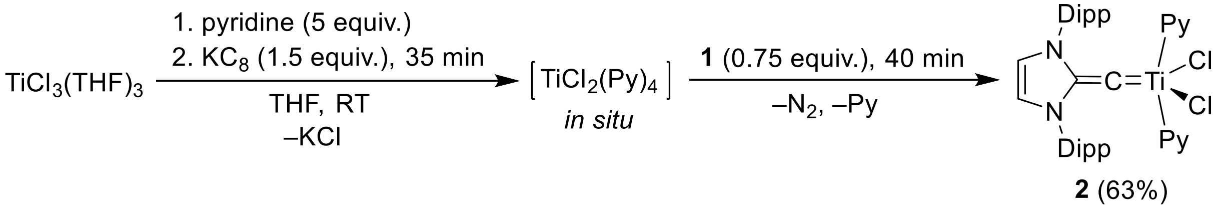

Compound 2: $TiCl_3(THF)_3$ (115 mg, 311 μmol, 1.00 equiv.) was dissolved in THF (15 mL) and pyridine (127 μL, 1.57 mmol, 5.04 equiv.) was added. Next, KC_8 (63.1 mg, 467 μmol, 1.50 equiv.) was added, using a small amount of THF to rinse out any remaining KC_8. The suspension was stirred for 35 min, and the color slowly changed from green to dark blue. Diazoolefin **1** (100 mg, 233 μmol, 0.750 eq.) was then added to the suspension, which resulted in rapid gas formation and a dark yellow mixture. The suspension was stirred for 40 min, after which the solvent was evaporated under reduced pressure. The product was then extracted with toluene (20 mL), and the solution was filtered through a glass frit. The solvent was removed under reduced pressure, and the dark solids were washed with pentane (3 x 10 mL). The solids were dried under reduced pressure, affording **2** as a dark yellow powder (100 mg, 63%). Complex **2** can be stored at – 40 °C for weeks as a solid, and only shows minor decomposition after days in solution at room temperature.

Crystals, suitable for single-crystal X-ray diffraction analysis, were grown by layering a benzene solution of **2** with diethyl ether. These crystals appeared yellow in color.

^{1}H NMR (800 MHz, C_6D_6) δ 11.51 (s, 1H, Py), 9.02 (s, 3H, Py), 7.06 (t, 2H, CH_{arom}, Dipp), 7.00 (d, J = 7.6 Hz, 4H, CH_{arom}, Dipp), 6.85 (s, 2H, Py), 6.50 (s, 4H, Py), 5.45 (s, 2H, $CH_{imidazole}$), 3.30 (hept, J = 6.9 Hz, 4H, C$\underline{H}$(CH_3)$_2$, Dipp), 1.67 (d, J = 6.9 Hz, 12H, CH_3, Dipp), 1.14 (d, J = 7.0 Hz, 12H, CH_3, Dipp).
^{13}C NMR (201 MHz, C_6D_6) δ 271.21 (C=$\underline{C}$=Ti), 148.03 (C_q, Dipp), 135.52 (C_q, Dipp), 129.84 (CH_{arom}, Dipp), 124.12 (CH_{arom}, Dipp), 116.01, 114.19 (C_q, imidazole), 29.10 ($\underline{C}$H(CH_3)$_2$, Dipp), 24.20 (CH_3, Dipp), 24.03 (CH_3, Dipp). *(Py peaks not detected due to broadening)*.
HRMS (nanochip-ESI/LTQ-Orbitrap) *m/z*: Only observed the [M + H^+] for the N-heterocyclic olefin ($C_{28}H_{38}N_2$).

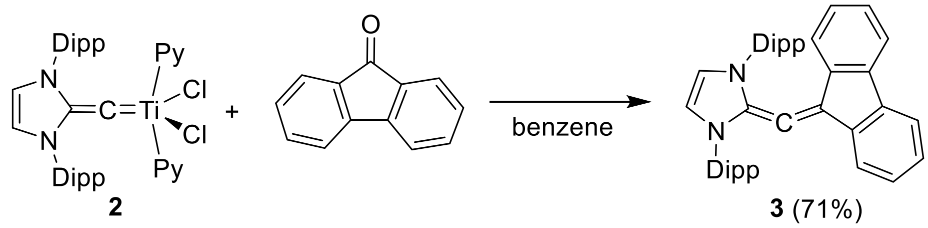

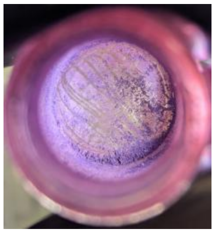

Compound 3: Complex **2** (10.0 mg, 14.8 μmol, 1.0 equiv.) and 9-fluorenone (3.1 mg, 17 μmol, 1.1 equiv.) were combined in a vial, and benzene (1 mL) was added. The mixture was stirred for 1 h, and the resulting suspension was filtered. The filtrate was dried under reduced pressure, and the product was extracted with pentane (2 x 5 mL). The pentane extracts were combined and filtered, after which the resulting filtrate solution was evaporated under reduced pressure to afford **3** as a violet solid (5.9 mg, 71%). Extra pure samples of **3** can be obtained by placing a concentrated pentane solution of **3** in the freezer at – 40 °C for 1 day, yielding violet crystals of **3** in ~ 40% overall yield. Compound **3** can be stored at – 40 °C for days as a solid, but shows decomposition after hours in solution at room temperature.

Alternative synthesis:

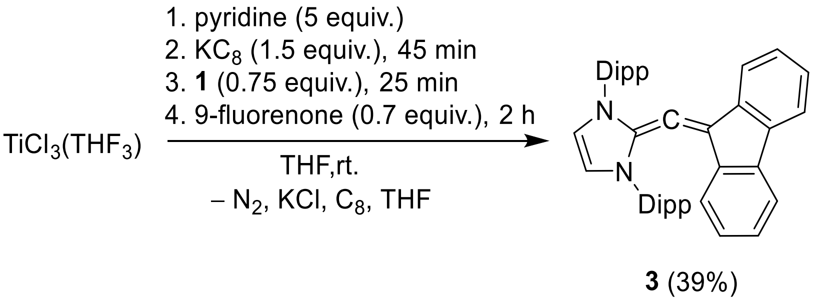

Compound 3: $TiCl_3(THF)_3$ (115 mg, 311 μmol, 1.00 equiv.) was dissolved in THF (10 mL) and pyridine (127 μL, 1.57 mmol, 5.00 equiv.) was added. Next, KC_8 (63.1 mg, 467 μmol, 1.50 equiv.) was added, using a small amount of THF to rinse out any remaining KC_8. The suspension was stirred for 45 min, and the color slowly changed from green to dark blue. Diazoolefin **1** (100 mg, 233 μmol, 0.75 equiv.) was then added to the suspension, which resulted in gas formation and a dark yellow mixture. The suspension was stirred for 25 min, after which it was filtered over celite. 9-Fluorenone (39.2 mg, 218 μmol, 0.70 equiv.) was then added to the filtrate while stirring, and the solution was stirred for 2 h. The volatiles were evaporated under reduced pressure, and the product was extracted with pentane (3 x 10 mL). The pentane extracts were combined and the solvent was removed under reduced pressure, affording **3** as a violet solid (51.6 mg, 39%).

Single crystals of **3** were obtained by placing a concentrated pentane solution in the freezer at – 40 °C for 1 day. These crystals appeared dark purple in color.

^{1}H NMR (400 MHz, C_6D_6) δ 7.79 (d, J = 7.5 Hz, 2H, CH_{arom}, fluorenylidene), 7.34 (d, J = 7.4 Hz, 2H, CH_{arom}, fluorenylidene), 7.19 (td, J = 7.4, 1.1 Hz, 2H, CH_{arom}, fluorenylidene), 7.07 (td, J = 7.4, 1.1 Hz, 2H, CH_{arom}, fluorenylidene), 6.99 – 6.75 (m, 6H, CH_{arom}, Dipp), 6.25 (s, 2H, $CH_{imidazole}$), 3.66 (hept, J = 6.9 Hz, 4H, $C\underline{H}(CH_3)_2$, Dipp), 1.33 (d, J = 6.9 Hz, 12H, CH_3, Dipp), 1.23 (d, J = 6.8 Hz, 12H, CH_3, Dipp).
^{13}C NMR (101 MHz, C_6D_6) δ 210.36 (C=$\underline{C}$=C), 150.80 (C_q, imidazole), 147.95 (C_q, Dipp), 141.21 (C_q, fluorenylidene), 138.96 (C_q, fluorenylidene), 134.41 (C_q, Dipp), 131.57 (C=$\underline{C}$(fluorenylidene)), 129.67 (CH_{arom}, Dipp), 127.32 (CH_{arom}, fluorenylidene), 126.70 (CH_{arom}, fluorenylidene), 124.42 (CH_{arom}, Dipp), 122.22 (CH_{arom}, fluorenylidene), 119.63 (CH_{arom}, fluorenylidene), 118.31 ($CH_{imidazole}$), 28.93 ($\underline{C}H(CH_3)_2$, Dipp), 25.49 (CH_3, Dipp), 23.60 (CH_3, Dipp).
HRMS (APCI/QTOF) *m/z*: $[M + H]^+$ Calculated for $C_{41}H_{45}N_2^+$ 565.3577; Found 565.3601.
UV/Vis (THF, λ_{max}, nm): 308 (ε = 1.7 x 10^4 $M^{-1}cm^{-1}$), 320 (ε = 1.66 x 10^4 $M^{-1}cm^{-1}$), 520 (ε = 830 $M^{-1}cm^{-1}$).

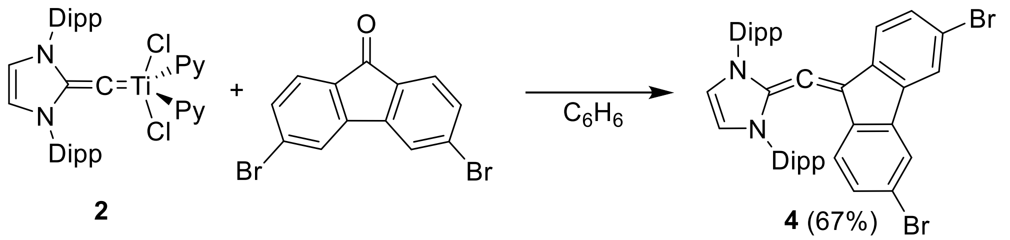

Compound 4: Complex **2** (20.0 mg, 29.5 µmol, 1.3 equiv.) and 3,6-dibromofluoren-9-one (7.8 mg, 23 µmol, 1.0 equiv.) were combined in a vial, and benzene (1 mL) was added. The mixture was stirred for 1.5 h, and the resulting suspension was filtered. The filtrate was dried under reduced pressure, and the product was extracted with pentane (2 x 5 mL). The pentane extracts were combined and filtered, after which the resulting filtrate solution was evaporated under reduced pressure to afford **4** as a light-brown/purple solid (11 mg, 67%). Compound **4** can be stored at – 40 °C for days as a solid, but shows decomposition after hours in solution at room temperature.

Single crystals of **4** were obtained by placing a concentrated pentane solution in the freezer at – 40 °C for 1 day. These crystals appeared light purple in color.

^{1}H NMR (400 MHz, C_6D_6) δ 7.45 (d, J = 8.0 Hz, 2H, 3,6-dibromofluorenylidene), 7.31 (dd, J = 8.0, 1.8 Hz, 2H, 3,6-dibromofluorenylidene), 7.24 (d, J = 1.8 Hz, 2H, CH_{arom}, 3,6-dibromofluorenylidene), 6.97 – 6.77 (m, 6H, CH_{arom}, Dipp), 6.21 (s, 2H, $CH_{imidazole}$), 3.50 (hept, J = 6.9 Hz, 4H, C$\underline{H}$(CH_3)$_2$, Dipp), 1.24 (d, J = 6.8 Hz, 12H, CH_3, Dipp), 1.18 (d, J = 6.9 Hz, 12H, CH_3, Dipp).

^{13}C NMR (101 MHz, C_6D_6) δ 216.16 (C=$\underline{C}$=C), 151.40 (C_q, imidazole), 147.76 (C_q, Dipp), 141.57 (C_q, 3,6-dibromofluorenylidene), 137.35 (C_q, 3,6-dibromofluorenylidene), 134.02 (C_q, Dipp), 130.02 (CH_{arom}, Dipp), 129.90 (CH_{arom}, 3,6-dibromofluorenylidene), 128.59 (C=$\underline{C}$, 3,6-dibromofluorenylidene), 124.50 (CH_{arom}, Dipp), 123.35 (CH_{arom}, 3,6-dibromofluorenylidene), 123.31 (CH_{arom}, 3,6-dibromofluorenylidene), 121.51 (C_q–Br), 118.56 ($CH_{imidazole}$), 28.92 ($\underline{C}$H(CH_3)$_2$, Dipp), 25.40 (CH_3, Dipp), 23.50 (CH_3, Dipp).

HRMS (ESI/QTOF) *m/z*: [M + H]$^+$ Calculated for $C_{41}H_{43}Br_2N_2^+$ 721.1788; Found 721.1789.

UV/Vis (THF, λ_{max}, nm): 315 (ε = 3.2 x 10^4 $M^{-1}cm^{-1}$), 330 (ε = 3.0 x 10^4 $M^{-1}cm^{-1}$), 542 (ε = 650 $M^{-1}cm^{-1}$).

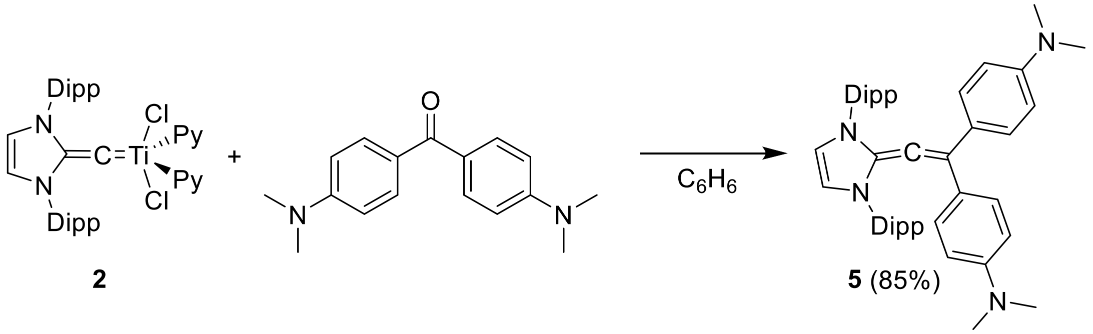

2 **5** (85%)

Compound 5: Complex **2** (15.5 mg, 23 μmol, 1.8 equiv.) and 4,4-bis(dimethylamino) benzophenone (3.5 mg, 13 μmol, 1.0 equiv.) were combined in a vial, and benzene (1 mL) was added. The mixture was stirred for 1 h, and the resulting suspension was filtered. The solvent was removed under reduced pressure, and the product was extracted with pentane (2 x 5 mL). The pentane extracts were combined and filtered, after which the resulting filtrate solution was evaporated under reduced pressure to afford **5** as a light-orange solid (7.2 mg, 85%).

^{1}H NMR (600 MHz, C_6D_6) δ 7.21 – 7.10 (m, 10H, CH_{arom}, C_6H_4–NMe_2 and Dipp (*overlap with solvent signal*), 6.58 – 6.51 (m, 4H, CH_{arom}, C_6H_4–NMe_2), 6.18 (s, 2H, $CH_{imidazole}$), 3.66 (hept, J = 6.9 Hz, 4H, C<u>H</u>(CH_3)$_2$, Dipp), 2.50 (s, 12H, N(CH_3)$_2$), 1.26 (dd, J = 8.0, 6.9 Hz, 24H, CH_3, Dipp).
^{13}C NMR (151 MHz, C_6D_6) δ 195.79 (C=<u>C</u>=C), 149.77 (C_q, C_6H_4–OMe or Dipp), 148.95 (C_q, C_6H_4–OMe or Dipp), 140.53 (C_q, imidazole), 136.29, 132.37, 131.97, 130.01, 128.96, 128.35, 124.22 (CH_{arom}), 116.99 ($CH_{imidazole}$), 112.18 (CH_{arom}, C_6H_4–NMe_2), 40.47 (N(CH_3)$_2$), 28.86 (<u>C</u>H(CH_3)$_2$, Dipp), 25.65 (CH_3, Dipp), 23.02 (CH_3, Dipp). *Some signals could not be assigned due to overlap with peaks in the 2D NMR spectra.*
HRMS (ESI/QTOF) *m/z*: [M + H]$^+$ Calculated for $C_{45}H_{57}N_4^+$ 653.4578; Found 653.4583.
UV/Vis (THF, λ_{max}, nm): 302 (ε = 1.7 x 10^4 $M^{-1}cm^{-1}$), 390 (ε = 0.47 x 10^4 $M^{-1}cm^{-1}$).

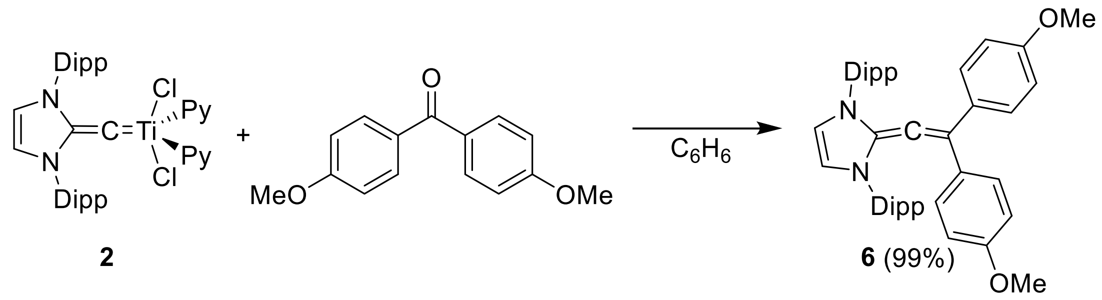

Compound 6: Complex **2** (15.2 mg, 22.4 μmol, 1.8 equiv.) and 4,4-dimethoxybenzophenone (3.1 mg, 13 μmol, 1.0 equiv.) were combined in a vial, and benzene (1 mL) was added. The mixture was stirred for 1 h, and the resulting suspension was filtered. The solution was removed under reduced pressure, and the product was extracted with pentane (2 x 5 mL). The pentane extracts were combined and filtered, after which the resulting filtrate solution was evaporated under reduced pressure to afford **6** as a light-orange solid (7.9 mg, 99%).

Single crystals of **6** were obtained by placing a concentrated pentane solution in the freezer at –40 °C for 1 day. These crystals appeared light-orange in color.

^{1}H NMR (600 MHz, C_6D_6) *δ* 7.15 (t, 2H, CH_{arom}, Dipp), 7.09 (d, *J* = 8.1 Hz, 4H, CH_{arom}, Dipp), 7.07 (d, *J* = 8.6 Hz, 4H, CH_{arom}, C_6H_4–OMe), 6.72 (d, *J* = 8.7 Hz, 4H, CH_{arom}, C_6H_4–OMe), 6.14 (s, 2H, $CH_{imidazole}$), 3.54 (hept, *J* = 6.9 Hz, 4H, C<u>H</u>(CH_3)$_2$, Dipp), 3.29 (s, 6H, OMe), 1.21 (d, *J* = 6.8 Hz, 12H, CH_3, Dipp), 1.19 (d, *J* = 6.9 Hz, 12H, CH_3, Dipp).
^{13}C NMR (151 MHz, C_6D_6) *δ* 199.72 (C=C=C), 159.08 (C_q, C_6H_4–OMe), 148.78 (C_q, Dipp), 141.61 (C_q, imidazole), 135.84 (C_q, Dipp), 135.53 (C_q, C_6H_4–OMe), 131.39 (C=<u>C</u>(C_6H_4OMe)$_2$, 130.11 (CH_{arom}, C_6H_4–OMe), 129.16 (CH_{arom}, Dipp), 124.27 (CH_{arom}, Dipp), 117.16 ($CH_{imidazole}$), 113.21 (CH_{arom}, C_6H_4–OMe), 54.75 (OMe), 28.84 (<u>C</u>H(CH_3)$_2$, Dipp), 25.57 (CH_3, Dipp), 22.86 (CH_3, Dipp).
HRMS (nanochip-ESI/LTQ-Orbitrap) *m/z*: $[M + H]^+$ calculated for $C_{43}H_{51}N_2O_2^+$ 627.3945; Found 627.3954.
UV/Vis (THF, λ_{max}, nm): 281 (ε = 3.2 x 10^4 $M^{-1}cm^{-1}$), 350 (ε = 0.75 x 10^4 $M^{-1}cm^{-1}$), 450 (ε = 0.22 x 10^4 $M^{-1}cm^{-1}$).

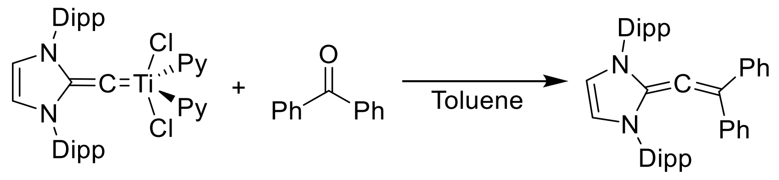

Compound 7: Complex **2** (22.1 mg, 32.6 μmol, 1.7 equiv.) and benzophenone (3.4 mg, 19 μmol,1.0 equiv.) were combined in a vial, and toluene (1 mL) was added. The mixture was stirred for 18 h, and the resulting suspension was filtered. The solution was removed under reduced pressure, and the product was extracted with pentane (2 x 5 mL). The pentane extracts were combined and filtered, after which the resulting filtrate solution was evaporated under reduced pressure to afford **7** as a light-orange solid (10 mg, 95%).

Single crystals of **7** were obtained by placing a concentrated pentane solution in the freezer at –40 °C for 1 day. These crystals appeared orange in color.

^{1}H NMR (600 MHz, C_6D_6) *δ* 7.15 – 7.12 (m, 2H, CH_{arom}, Dipp), 7.09 – 7.02 (m, 12H, CH_{arom}, Ph and Dipp), 7.02 – 6.96 (m, 2H, CH_{arom}, Ph), 6.14 (s, 2H, $CH_{imidazole}$), 3.48 (hept, *J* = 6.9 Hz, 4H, C<u>H</u>$(CH_3)_2$, Dipp), 1.20 (d, *J* = 6.9 Hz, 12H, CH_3, Dipp), 1.13 (d, *J* = 6.8 Hz, 12H, CH_3, Dipp).
^{13}C NMR (151 MHz, C_6D_6) *δ* 204.23 (C=<u>C</u>=C), 148.68 (C_q, Dipp), 142.46 (C_q, imidazole), 135.56 (C_q, Dipp), 132.41 (C=<u>C</u>Ph_2), 129.26 (CH_{arom}, Dipp), 128.90 (C_{arom}, Ph), 128.35 (C_{arom}, Ph), 127.75 (C_{arom}, Ph), 126.55 (CH_{arom}, Ph), 124.28 (CH_{arom}, Dipp), 117.31 ($CH_{imidazole}$), 28.84 (<u>C</u>H$(CH_3)_2$, Dipp), 25.57 (CH_3, Dipp), 22.75 (CH_3, Dipp).
HRMS (ESI/QTOF) *m/z*: $[M + H]^+$ Calculated for $C_{41}H_{47}N_2^+$ 567.3734; Found 567.3742.
UV/Vis (THF, λ_{max}, nm): 330 (ε = 0.50 x 10^4 $M^{-1}cm^{-1}$), 456 (ε = 0.075 x 10^4 $M^{-1}cm^{-1}$).

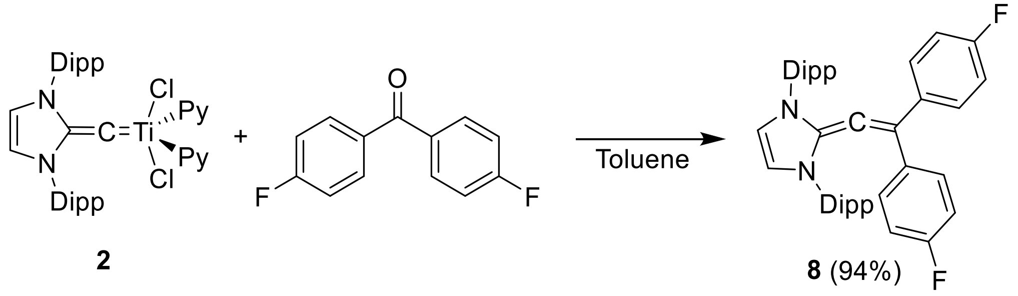

2

8 (94%)

Compound 8: Complex **2** (14 mg, 20.7 μmol, 1.8 equiv.) and 4,4'-difluorobenzophenone (2.5 mg, 12 μmol, 1.0 equiv.) were combined in a vial, and toluene (1 mL) was added. The mixture was stirred for 18 h, and the resulting suspension was filtered. The solution was removed under reduced pressure, and the product was extracted with pentane (2 x 5 mL). The pentane extracts were combined and filtered, after which the resulting filtrate solution was evaporated under reduced pressure to afford **8** as an orange solid (6.5 mg, 94%).

Single crystals of **8** were obtained by layering a THF solution with pentane. These crystals appeared light-orange in color.

^{1}H NMR (400 MHz, C_6D_6) δ 7.16 – 7.09 (m, 2H, CH_{arom}, Dipp), 7.04 (d, J = 7.2 Hz, 4H, CH_{arom}, Dipp), 6.87 – 6.78 (m, 4H, CH_{arom}, C_6H_4–F), 6.80 – 6.66 (m, 4H, CH_{arom}, C_6H_4–F), 6.10 (s, 2H, $CH_{imidazole}$), 3.37 (hept, J = 6.8 Hz, 4H, C<u>H</u>(CH_3)$_2$, Dipp), 1.16 (d, J = 6.9 Hz, 12H, CH_3, Dipp), 1.07 (d, J = 6.9 Hz, 12H, CH_3, Dipp).
^{13}C NMR (101 MHz, C_6D_6) δ 204.35 (C=<u>C</u>=C), 162.33 (d, J = 244.9 Hz, C_q, C–F), 148.52 (C_q, Dipp), 142.82 (C_q, imidazole), 138.51 (d, J = 3.2 Hz, C_q, C_6H_4–F), 135.32 (C_q, Dipp), 130.28 (d, J = 7.6 Hz, CH_{arom}, C_6H_4–F), 130.08 (C=<u>C</u>(C_6H_4F)$_2$), 129.38 (CH_{arom}, Dipp), 124.31 (CH_{arom}, Dipp), 117.38 ($CH_{imidazole}$), 114.49 (d, J = 21.1 Hz, CH_{arom}, C_6H_4–F), 28.80 (<u>C</u>H(CH_3)$_2$, Dipp), 25.47 (CH_3, Dipp), 22.67 (CH_3, Dipp).
^{19}F NMR (376 MHz, C_6D_6) δ -116.55.
HRMS (ESI/QTOF) *m/z*: $[M + H]^+$ Calculated for $C_{41}H_{45}F_2N_2^+$ 603.3545; Found 603.3544.
UV/Vis (THF, λ_{max}, nm): 325 (ε = 0.61 x 10^4 $M^{-1}cm^{-1}$), 453 (ε = 0.093 x 10^4 $M^{-1}cm^{-1}$).

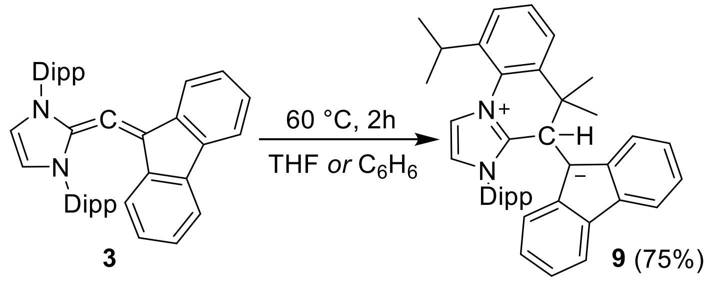

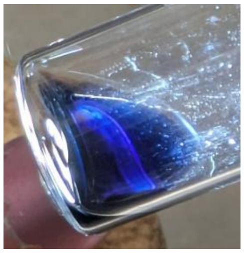

THF solution

Compound 9: Compound **3** (16.9 mg, 29.9 μmol) was dissolved in deuterated THF (0.6 mL) and placed in an NMR tube. The tube was heated to 60 °C for 2 h, during which the color of the solution turned dark blue. The compound was obtained in quantitative yield, as determined by an NMR spectroscopic analysis. The volatiles were then removed under reduced pressure, and the solids were washed with toluene (2 x 1 mL) and pentane (2 x 5 mL). The solids were then dried under reduced pressure, giving compound **9** as a deep red-purple solid (12.6 mg, 75%).

*Compound **9** can also be made by heating a benzene solution of **3** at 60 °C for 2 h. Due to the low solubility of **9**, most of the product precipitates as red-purple solids. Dissolving this precipitate in THF gave an identical NMR spectrum as was obtained by heating a THF solution of **3** directly.*

^{1}H NMR (500 MHz, THF) δ 8.08 (d, J = 2.2 Hz, 1H, $CH_{imidazole}$), 7.78 (d, J = 7.6 Hz, 1H, CH_{arom}, fluorenylidene), 7.76 – 7.69 (m, 2H, $CH_{imidazole}$ and CH_{arom}, fluorenylidene), 7.64 (dd, J = 8.0, 1.5 Hz, 1H, CH_{arom}, Dipp), 7.41 (dd, J = 7.9, 1.4 Hz, 1H, CH_{arom}, Dipp), 7.33 (dt, J = 19.0, 7.8 Hz, 2H, CH_{arom}, Dipp), 7.26 (dd, J = 7.6, 1.5 Hz, 1H, CH_{arom}, Dipp), 6.67 (dd, J = 7.8, 1.4 Hz, 1H, CH_{arom}, Dipp), 6.56 (t, J = 7.4 Hz, 1H, CH_{arom}, fluorenylidene), 6.45 (d, J = 7.8 Hz, 1H, CH_{arom}, fluorenylidene), 6.41 (t, J = 7.5 Hz, 1H, CH_{arom}, fluorenylidene), 6.34 (t, 1H, CH_{arom}, fluorenylidene), 6.29 (t, J = 7.7 Hz, 1H, CH_{arom}, fluorenylidene), 5.88 (d, J = 8.4 Hz, 1H, CH_{arom}, fluorenylidene), 4.67 (s, 1H, imidazole–C$\underline{H}$–fluorenylidene), 3.82 (hept, J = 6.7 Hz, 1H, $C\underline{H}(CH_3)_2$, Dipp), 2.65 (hept, J = 6.8 Hz, 1H, $C\underline{H}(CH_3)_2$, Dipp), 1.67 (d, J = 6.7 Hz, 3H, CH_3, Dipp), 1.56 (dd, J = 6.7, 3.5 Hz, 6H, CH_3, Dipp), 1.40 (hept, J = 6.7 Hz, 1H, $C\underline{H}(CH_3)_2$, Dipp), 1.34 (s, 3H, CH_3, Ph–C(C$\underline{H_3}$)$_2$–CH), 1.23 (d, J = 6.8 Hz, 3H, CH_3, Dipp), 1.14 (s, 3H, CH_3, Ph–C(C$\underline{H_3}$)$_2$–CH), 0.51 (d, J = 6.9 Hz, 3H, CH_3, Dipp), -0.76 (d, J = 6.5 Hz, 3H, CH_3, Dipp).

^{13}C NMR (126 MHz, THF) δ 151.88 (C_q, imidazole), 148.62 (C_q, Dipp), 144.93 (C_q, Dipp), 139.90 (C_q, Dipp), 139.80 (C_q, Dipp), 137.91 (C_q, fluorenylidene), 134.13 (C_q, fluorenylidene), 131.91 (CH_{arom}, Dipp), 131.31 (C_q, Dipp), 130.37 (C_q, Dipp), 130.17 (CH_{arom}, Dipp), 127.53 (CH_{arom}, Dipp), 125.27 (C_q, fluorenylidene), 125.25 (CH_{arom}, Dipp), 125.21 (CH_{arom}, Dipp), 124.49 (CH_{arom}, Dipp), 124.05 ($CH_{imidazole}$), 120.54 (CH_{arom}, fluorenylidene), 120.46 (CH_{arom}, fluorenylidene), 120.22 ($CH_{imidazole}$), 119.05 (CH_{arom}, fluorenylidene), 118.55 (CH_{arom}, fluorenylidene), 112.71 (CH_{arom},

fluorenylidene), 111.95 (CH_{arom}, fluorenylidene), 109.93 (CH_{arom}, fluorenylidene), 109.32 (CH_{arom}, fluorenylidene), 83.36 (HC–$\underline{C}_{q,fluorenylidene}$), 43.58 (imidazole–$\underline{C}$H–fluorenylidene), 40.52 (Ph–$\underline{C}(CH_3)_2$–CH), 31.35 (Ph–C($\underline{C}H_3)_2$–CH), 29.41 ($\underline{C}H(CH_3)_2$, Dipp), 29.38 ($\underline{C}H(CH_3)_2$, Dipp), 29.06 ($\underline{C}H(CH_3)_2$, Dipp), 25.96 (CH_3, Dipp), 25.68 (CH_3, Dipp), 24.58 (CH_3, Dipp), 24.13 (Ph–C($\underline{C}H_3)_2$–CH), 23.78 (CH_3, Dipp), 19.38 (CH_3, Dipp). *One C_q of the Fluorenylidene group is missing, likely due to overlap with another peak.*

HRMS (ESI/QTOF) *m/z*: $[M + H]^+$ Calculated for $C_{41}H_{45}N_2^+$ 565.3577; Found 565.3580.

UV/Vis (THF, λ_{max}, nm): 316 (ε = 1.14 x 10^4 $M^{-1}cm^{-1}$), 362 (ε = 0.50 x 10^4 $M^{-1}cm^{-1}$), 444 (ε = 0.07 x 10^4 $M^{-1}cm^{-1}$), 468 (ε = 0.09 x 10^4 $M^{-1}cm^{-1}$), 502 (ε = 0.10 x 10^4 $M^{-1}cm^{-1}$), 594 (ε = 0.14 x 10^4 $M^{-1}cm^{-1}$).

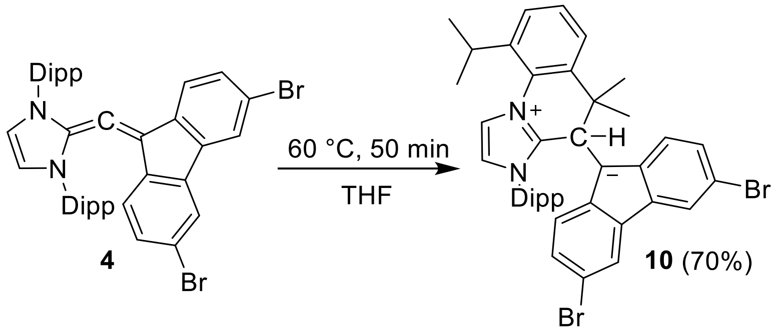

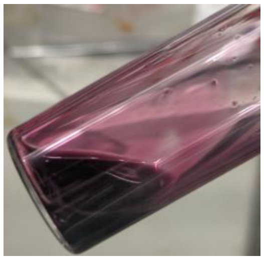

THF solution

Compound 10: Compound **4** (6.1 mg, 8.44 μmol) was dissolved in deuterated THF (0.6 mL) and placed in an NMR tube. The tube was heated to 60 °C for 50 min, during which the color of the solution turned dark violet-red. The compound was obtained in quantitative yield, as determined by spectroscopic analysis. The volatiles were then removed under reduced pressure, and the solids were dissolved in diethyl ether, after which a solid quickly started to crystallize. The suspension was left for 1 h to let all the product crystallize, after which the diethyl ether was then removed from the solids. The solids were then washed with pentane (3 x 1 mL) and dried under reduced pressure, giving compound **10** as a deep red-purple solid (4.3 mg, 70%).

Single crystals of **10** were obtained by making a concentrated benzene solution of **4**, which was left under inert atmosphere for 3 days. These crystals appeared dark red in color.

^{1}H NMR (600 MHz, THF, 254 K) δ 8.23 (d, J = 2.3 Hz, 1H, $CH_{imidazole}$), 8.03 (d, J = 2.2 Hz, 1H, $CH_{imidazole}$), 7.85 (d, J = 15.8 Hz, 2H, CH_{arom}, fluorenylidene), 7.70 (d, J = 8.0 Hz, 1H, CH_{arom}, Dipp), 7.49 (d, J = 7.8 Hz, 1H, CH_{arom}, Dipp), 7.39 (t, J = 7.8 Hz, 2H, CH_{arom}, Dipp), 7.24 (d, J = 7.6 Hz, 1H, CH_{arom}, Dipp), 6.78 (d, J = 7.7 Hz, 1H, CH_{arom}, Dipp), 6.65 (d, J = 8.5 Hz, 1H, CH_{arom}, fluorenylidene), 6.46 (d, J = 8.7 Hz, 1H, CH_{arom}, fluorenylidene), 6.41 (br, 1H, CH_{arom}, fluorenylidene), 5.78 (br, 1H, CH_{arom}, fluorenylidene), 4.55 (br, 1H, imidazole–C$\underline{H}$–fluorenylidene), 3.82 (hept, J = 6.7 Hz, 1H, $C\underline{H}(CH_3)_2$, Dipp), 2.62 (hept, J = 6.9 Hz, 1H, $C\underline{H}(CH_3)_2$, Dipp), 1.69 (d, J = 6.6 Hz, 3H, CH_3, Dipp), 1.55 (dd, J = 7.1, 7.1 Hz, 6H, CH_3, Dipp), 1.38 (hept, J = 6.7 Hz, 1H, $C\underline{H}(CH_3)_2$, Dipp), 1.33 (s, 3H, Ph–$C(C\underline{H_3})_2$–CH), 1.24 (d, J = 6.7 Hz, 3H, CH_3, Dipp), 1.04 (s, 3H, Ph–$C(C\underline{H_3})_2$–CH), 0.58 (d, J = 6.9 Hz, 3H, CH_3, Dipp), -0.75 (d, J = 6.5 Hz, 3H, CH_3, Dipp).

^{13}C NMR (151 MHz, THF, 254 K) δ 150.74 (C_q, imidazole), 148.08 (C_q, Dipp), 144.86 (C_q, Dipp), 139.86 (C_q, Dipp), 139.28 (C_q, Dipp), 135.91 (C_q, fluorenylidene), 132.26 (CH_{arom}, Dipp), 131.96 (C_q, fluorenylidene), 131.02 (C_q, Dipp), 130.55 (C_q, Dipp), 129.91 (CH_{arom}, Dipp), 127.92 (CH_{arom}, Dipp), 125.27 (CH_{arom}, Dipp), 124.79 (CH_{arom}, Dipp), 124.23 ($CH_{imidazole}$), 123.27 (CH_{arom}, fluorenylidene), 123.06 (CH_{arom}, fluorenylidene), 121.70 (CH_{arom}, fluorenylidene), 121.31 (CH_{arom}, fluorenylidene), 121.10 ($CH_{imidazole}$), 114.89 (CH_{arom}, fluorenylidene), 114.17 (CH_{arom},

fluorenylidene), 103.37 (C–Br), 102.81 (C–Br), 85.41 (HC–$\underline{C}_{q,fluorenylidene}$), 42.93 (imidazole–$\underline{C}$H–fluorenylidene), 40.46 (Ph–$\underline{C}(CH_3)_2$–CH), 30.93 (Ph–C($\underline{C}H_3)_2$–CH), 29.48 ($\underline{C}H(CH_3)_2$, Dipp), 29.34 ($\underline{C}H(CH_3)_2$, Dipp), 29.30 ($\underline{C}H(CH_3)_2$, Dipp), 25.94 (CH_3, Dipp), 25.61 (CH_3, Dipp), 24.59 (CH_3, Dipp), 23.99 (CH_3, Dipp or Ph–C($\underline{C}H_3)_2$–CH), 23.59 (CH_3, Dipp or Ph–C($\underline{C}H_3)_2$–CH), 19.29 (CH_3, Dipp).

HRMS (nanochip-ESI/LTQ-Orbitrap) *m/z*: $[M + H]^+$ Calculated for $C_{41}H_{43}Br_2N_2^+$ 721.1788; Found 721.1791.

UV/Vis (THF, λ_{max}, nm): 316 (ε = 3.92 x 10^4 $M^{-1}cm^{-1}$), 374 (ε = 1.65 x 10^4 $M^{-1}cm^{-1}$), 435 (ε = 0.12 x 10^4 $M^{-1}cm^{-1}$), 463 (ε = 0.22 x 10^4 $M^{-1}cm^{-1}$), 497 (ε = 0.39 x 10^4 $M^{-1}cm^{-1}$), 535 (ε = 0.50 x 10^4 $M^{-1}cm^{-1}$).

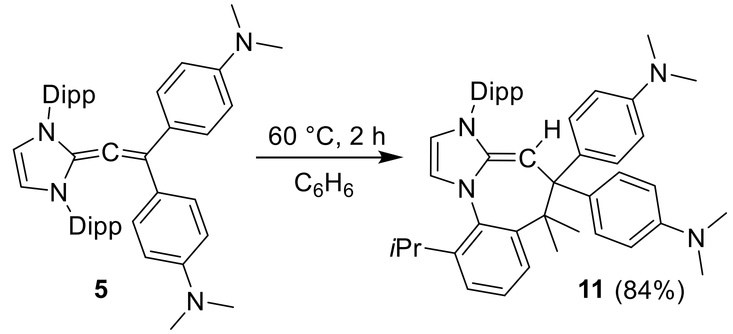

Compound 11: Compound **5** (7.4 mg, 11.3 µmol) was dissolved in benzene and placed in an NMR tube. The tube was heated to 60 °C for 2 h, during which no color change was observed. The compound was obtained in nearly quantitative yield, as determined by an NMR spectroscopic analysis. The volatiles were then removed under reduced pressure, and the product was extracted with pentane (2 x 5 mL). The pentane extracts were combined, and the solvent was removed under reduced pressure, giving compound **11** as a light-yellow solid (6.2 mg, 84%).

Single crystals of **11** were obtained by making a concentrated pentane solution of **11**, which was left in the freezer at – 40 °C for 3 days. These crystals appeared light-yellow in color.

^{1}H NMR (600 MHz, C_6D_6) *δ* 7.49 (d, *J* = 8.4 Hz, 2H, CH_{arom}, $C_6\underline{H}_4$–NMe_2), 7.37 – 7.27 (m, 3H, CH_{arom}, Dipp and $C_6\underline{H}_4$–NMe_2), 7.21 – 7.17 (m, 2H, CH_{arom}, Dipp , *overlap with C_6D_6*), 7.12 (dd, *J* = 6.9, 2.3 Hz, 1H, CH_{arom}, Dipp), 7.01 (dd, *J* = 7.7, 1.5 Hz, 1H, CH_{arom}, Dipp), 6.96 (t, *J* = 7.7 Hz, 1H, CH_{arom}, Dipp), 6.49 (d, *J* = 8.9 Hz, 2H, CH_{arom}, $C_6\underline{H}_4$–NMe_2), 6.43 (d, *J* = 8.9 Hz, 2H, CH_{arom}, $C_6\underline{H}_4$–NMe_2), 5.98 – 5.82 (m, 2H, $CH_{imidazole}$), 4.00 (s, 1H, imidazole=CH), 3.64 (dhept, *J* = 10.3, 3.6 Hz, 2H, $C\underline{H}(CH_3)_2$, Dipp), 3.50 (hept, *J* = 7.0 Hz, 1H, $C\underline{H}(CH_3)_2$, Dipp), 2.48 (s, 6H, $N(CH_3)_3$), 2.47 (s, 6H, $N(CH_3)_3$), 2.02 (s, 3H, Ph–$C(C\underline{H}_3)_2$–C), 1.73 (s, 3H, Ph–$C(C\underline{H}_3)_2$–C), 1.55 (d, *J* = 6.9 Hz, 3H, CH_3, Dipp), 1.35 (d, *J* = 6.9 Hz, 3H, CH_3, Dipp), 1.30 – 1.12 (m, 9H, CH_3, Dipp), 0.90 (d, *J* = 6.7 Hz, 3H, CH_3, Dipp).

^{13}C NMR (151 MHz, C_6D_6) *δ* 149.33 (C_q, Dipp), 149.29 (C_q, Dipp), 148.51 ($\underline{C}$–NMe_2), 147.88 ($\underline{C}$–NMe_2), 144.66 (C_q, Dipp), 142.68 (C_q, imidazole), 142.38 (C_q, Dipp), 140.72 (C_q, C_6H_4–NMe_2), 139.51 (C_q, C_6H_4–NMe_2), 137.64 (C_q, Dipp), 134.86 (C_q, Dipp), 131.97 (CH_{arom}, C_6H_4–NMe_2), 130.85 (CH_{arom}, C_6H_4–NMe_2), 129.34 (CH_{arom}, Dipp), 124.73 (CH_{arom}, Dipp), 124.59 (CH_{arom}, Dipp), 124.58 (CH_{arom}, Dipp), 124.57 (CH_{arom}, Dipp), 124.06 (CH_{arom}, Dipp), 116.00 ($CH_{imidazole}$), 115.69 ($CH_{imidazole}$), 111.91 (CH_{arom}, C_6H_4–NMe_2), 111.45 (CH_{arom}, C_6H_4–NMe_2), 79.25 (imidazole=$\underline{C}$H–C), 58.43 (imidazole=CH–$\underline{C}$), 49.12 (Ph–$\underline{C}(CH_3)_2$–C), 40.57 ($N(CH_3)_3$), 40.43 ($N(CH_3)_3$), 29.74 (Ph–$C(\underline{C}H_3)_2$–C), 29.00 ($\underline{C}H(CH_3)_2$, Dipp), 28.88 ($\underline{C}H(CH_3)_2$, Dipp), 28.67 ($\underline{C}H(CH_3)_2$, Dipp), 27.70 (Ph–

C(<u>C</u>H_3)$_2$–C), 25.78 (CH_3, Dipp), 25.30 (CH_3, Dipp), 25.04 (CH_3, Dipp), 23.98 (CH_3, Dipp), 23.30 (CH_3, Dipp), 22.65 (CH_3, Dipp).

HRMS (nanochip-ESI/LTQ-Orbitrap) *m/z*: $[M + H]^+$ Calculated for $C_{45}H_{57}N_4^+$ 653.4578; Found 653.4590.

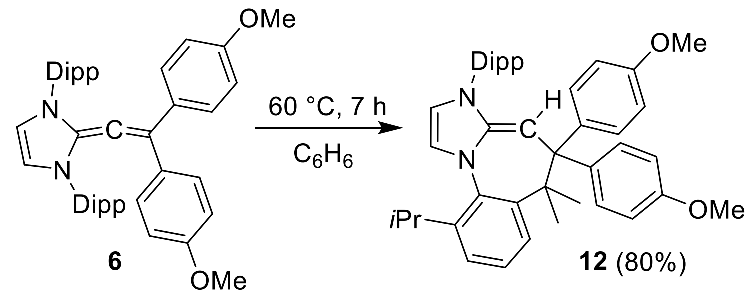

6 **12** (80%)

Compound 12: Compound **6** (8.4 mg, 13.4 µmol) was dissolved in C_6D_6 and placed in an NMR tube. The tube was heated to 60 °C for 7 h, during which no color change was observed. The compound was obtained in nearly quantitative yield, as determined by an NMR spectroscopic analysis. The volatiles were then removed under reduced pressure, and the product was extracted with pentane (2 x 5 mL). The pentane extracts were combined, and the solvent was removed under reduced pressure, giving compound **12** as a light-yellow solid (6.7 mg, 80%).

^{1}H NMR (600 MHz, C_6D_6) δ 7.43 (d, J = 8.4 Hz, 2H, CH_{arom}, $C_6\underline{H}_4$–OMe), 7.28 (d, J = 8.9 Hz, 2H, CH_{arom}, $C_6\underline{H}_4$–OMe), 7.21 – 7.11 (m, 3H, CH_{arom}, Dipp), 7.11 – 7.05 (m, 1H, CH_{arom}, Dipp), 7.02 – 6.95 (m, 1H, CH_{arom}, Dipp), 6.92 (t, J = 7.7 Hz, 1H, CH_{arom}, Dipp), 6.67 (d, J = 8.6 Hz, 2H, CH_{arom}, $C_6\underline{H}_4$–OMe), 6.58 (d, J = 8.8 Hz, 2H, CH_{arom}, $C_6\underline{H}_4$–OMe), 6.01 – 5.61 (m, 2H, $CH_{imidazole}$), 3.85 (s, 1H, imidazole=CH), 3.62 (hept, J = 6.8 Hz, 1H, $C\underline{H}(CH_3)_2$, Dipp), 3.55 (hept, J = 6.9 Hz, 1H, $C\underline{H}(CH_3)_2$, Dipp), 3.40 (hept, J = 7.0 Hz, 1H, $C\underline{H}(CH_3)_2$, Dipp), 3.27 (s, 3H, OCH_3), 3.26 (s, 3H, OCH_3), 1.96 (s, 3H, Ph–$C(C\underline{H}_3)_2$–C), 1.63 (s, 3H, Ph–$C(C\underline{H}_3)_2$–C), 1.46 (d, J = 6.8 Hz, 3H, CH_3, Dipp), 1.28 (d, J = 7.0 Hz, 3H, CH_3, Dipp), 1.23 (d, J = 7.1 Hz, 3H, CH_3, Dipp), 1.21 (dd, J = 7.0, 7.0 Hz, 6H, CH_3, Dipp), 0.88 (d, J = 6.7 Hz, 3H, CH_3, Dipp).

^{13}C NMR (151 MHz, C_6D_6) δ 157.78 ($\underline{C}$–OMe), 157.10 ($\underline{C}$–OMe), 149.23 (C_q, Dipp), 149.15 (C_q, Dipp), 144.32 (C_q, Dipp, imidazole or C_6H_4OMe), 144.03 (C_q, Dipp, imidazole or C_6H_4OMe), 142.83 (C_q, Dipp, imidazole or C_6H_4OMe), 142.81 (C_q, Dipp, imidazole or C_6H_4OMe), 142.65 (C_q, Dipp, imidazole or C_6H_4OMe), 137.40 (C_q, Dipp), 134.58 (C_q, Dipp), 132.28 (CH_{arom}, C_6H_4–OMe), 131.12 (CH_{arom}, C_6H_4–OMe), 129.47 (CH_{arom}, Dipp), 124.94 (CH_{arom}, Dipp), 124.65 (CH_{arom}, Dipp), 124.55 (CH_{arom}, Dipp), 124.51 (CH_{arom}, Dipp), 124.28 (CH_{arom}, Dipp), 116.03 ($CH_{imidazole}$), 115.54 ($CH_{imidazole}$), 112.68 (CH_{arom}, C_6H_4OMe), 111.93 (CH_{arom}, C_6H_4–OMe), 78.52 (imidazole=$\underline{C}$H–C), 59.02 (imidazole=CH–$\underline{C}$), 54.62 (O–CH_3), 54.59 (O–CH_3), 48.78 (Ph–$\underline{C}(CH_3)_2$–C), 29.41 (Ph–$C(\underline{C}H_3)_2$–C), 28.92 ($\underline{C}H(CH_3)_2$, Dipp), 28.87 ($\underline{C}H(CH_3)_2$, Dipp), 28.68 ($\underline{C}H(CH_3)_2$, Dipp), 27.58 (Ph–$C(\underline{C}H_3)_2$–C), 25.61 (CH_3, Dipp), 25.20 (CH_3, Dipp), 24.99 (CH_3, Dipp), 23.82 (CH_3, Dipp), 23.22 (CH_3, Dipp), 22.64 (CH_3, Dipp).

HRMS (nanochip-ESI/LTQ-Orbitrap) *m/z*: $[M + H]^+$ Calculated for $C_{43}H_{51}N_2O_2^+$ 627.3945; Found 627.3953.

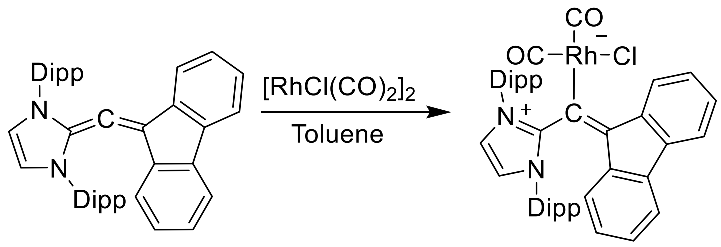

3 **13** (33%)

Complex 13: Compound **3** (10 mg, 18 μmol, 1.0 equiv.) and $[RhCl(CO)_2]_2$ (3.4 mg, 8.8 μmol, 0.49 equiv.) were combined in a vial, and toluene (2 mL) was added. The solution was stirred for 15 minutes, and the solvent was removed under reduced pressure. The solids were washed with pentane (3 x 3 mL), and were then redissolved in a minimal amount of toluene until almost all solids were dissolved. The solution was then placed in a freezer at – 40 °C for 1 day, after which light-yellow solids had formed. The solvent was then removed using a glass pipette, and the resulting solids were washed with pentane (5 mL) and dried under reduced pressure to afford complex **13** (4.5 mg, 33%).

Single crystals of **13** were obtained by layering a benzene solution of **13** with pentane. The crystals appeared yellow in color.

^{1}H NMR (600 MHz, CD_2Cl_2) δ 9.65 (d, J = 7.8 Hz, 1H), 7.71 (d, J = 7.6 Hz, 1H), 7.63 (d, J = 7.5 Hz, 1H), 7.41 (t, J = 7.7 Hz, 1H), 7.39 – 7.32 (m, 1H, CH_{arom}), 7.32 – 7.26 (m, 2H), 7.25 – 7.18 (m, 2H), 7.16 (td, J = 7.6, 1.2 Hz, CH_{arom}, Dipp), 7.09 (dd, J = 7.7, 1.6 Hz, 1H, CH_{arom}, Dipp), 7.05 (dd, J = 7.7, 1.5 Hz, 1H, CH_{arom}, Dipp), 6.27 (d, J = 7.8 Hz, 1H), 3.19 (dhept, J = 20.6, 6.8 Hz, 2H, C<u>H</u>$(CH_3)_2$, Dipp), 2.89 (dhept, J = 26.9, 6.8 Hz, 2H, C<u>H</u>$(CH_3)_2$, Dipp), 1.64 (d, J = 6.7 Hz, 3H, CH_3, Dipp), 1.60 (d, J = 6.7 Hz, 3H, CH_3, Dipp), 1.30 (d, J = 6.8 Hz, 3H, CH_3, Dipp), 1.24 (d, J = 6.7 Hz, 3H, CH_3, Dipp), 0.88 (d, J = 6.9 Hz, 3H, CH_3, Dipp), 0.85 (d, J = 6.9 Hz, 3H, CH_3, Dipp), 0.21 (d, J = 6.7 Hz, 3H, CH_3, Dipp), 0.16 (d, J = 6.7 Hz, 3H, CH_3, Dipp). *The hydrogen atoms in the aromatic region could not be fully assigned due to a high density of peaks, causing overlap of the 2D peaks in the 2D spectra.*

^{13}C NMR (151 MHz, CD_2Cl_2) δ 186.88 (d, J = 54.7 Hz, CO), 183.30 (d, J = 77.7 Hz, CO), 160.39, 157.43, 157.25, 150.50 (d, J = 1.7 Hz), 147.56, 147.49, 146.48, 146.10, 141.82, 141.80 (d, J = 1.4 Hz) 140.69, 135.44 (d, J = 2.1 Hz), 134.45, 134.36, 131.80, 130.90, 128.30, 128.25, 127.68, 126.66, 126.21, 125.80, 125.44, 125.31, 125.14, 125.04, 124.98, 124.40, 119.65 (CH_{arom}, fluorenylidene), 118.75 (CH_{arom}, fluorenylidene), 30.21 (<u>C</u>H$(CH_3)_2$, Dipp), 29.85 (<u>C</u>H$(CH_3)_2$, Dipp), 29.09 (<u>C</u>H$(CH_3)_2$, Dipp), 28.94 (<u>C</u>H$(CH_3)_2$, Dipp), 26.80 (CH_3, Dipp), 26.77 (CH_3, Dipp), 24.88 (CH_3, Dipp), 24.84 (CH_3, Dipp), 24.00 (CH_3, Dipp), 23.64 (CH_3, Dipp), 21.85 (CH_3, Dipp), 21.41

(CH_3, Dipp). *The carbon atoms in the aromatic region could not be fully assigned due to a high density of peaks, causing overlap of the 2D peaks in the 2D spectra.*

HRMS (nanochip-ESI/LTQ-Orbitrap) *m/z*: $[M–Cl]^+$ Calculated for $C_{43}H_{44}N_2RhO_2^+$ 723.2458; Found 723.2507.

FT-IR (solid, cm^{-1}): 2051 (νCO_{symm}), 1979 (νCO_{asymm}).

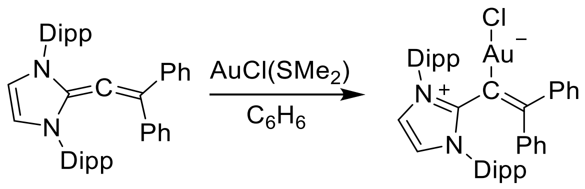

7 **14** (50%)

Complex 14: Compound **7** (10.9 mg, 19.2 µmol, 1.0 equiv.) and $AuCl(SMe_2)$ (5.6 mg, 19 µmol, 0.99 equiv.) were combined in a vial, and benzene (1 mL) was added. The solution was stirred for 15 min, and a crystalline precipitate was observed. Pentane (2 mL) was then added to the mixture, and the suspension was filtered. The light-yellow solids were then extracted with DCM (1 mL), and the resulting filtrate was dried under reduced pressure. The oily residue was suspended in cold toluene (1 mL), which resulted in the formation of crystals. The mixture was then placed in a freezer at – 40 °C for 1 h, after which it was filtered. The crystals were washed with more cold toluene (1 x 1 mL) and pentane (3 x 1 mL) to afford **14** as a light-beige solid (7.7 mg, 50%).

Single crystals of **14** were obtained by layering a benzene solution of **14** with pentane. The crystals appeared light-orange in color.

^{1}H NMR (600 MHz, CD_2Cl_2, 270K) δ 7.48 (t, J = 7.8 Hz, 2H CH_{arom}, Dipp), 7.36 (d, J = 7.7 Hz, 2H, CH_{arom}, Dipp), 7.33 (s, 2H, $CH_{imidazole}$), 7.21 – 7.17 (m, 2H, CH_{arom}, Dipp), 7.17 – 7.12 (m, 2H, CH_{arom}, Ph), 7.09 (t, J = 7.8 Hz, 4H, CH_{arom}, Ph), 6.82 (d, J = 6.7 Hz, 2H, CH_{arom}, Ph), 6.65 (d, J = 7.0 Hz, 2H, CH_{arom}, Ph), 2.89 (dhept, J = 12.9, 6.8 Hz, 4H, $C\underline{H}(CH_3)_2$, Dipp), 1.55 (d, J = 6.6 Hz, 6H, CH_3, Dipp), 1.18 (d, J = 6.7 Hz, 6H, CH_3, Dipp), 1.04 (d, J = 6.8 Hz, 6H, CH_3, Dipp), 0.76 (d, J = 6.7 Hz, 6H, CH_3, Dipp).

^{13}C NMR (151 MHz, CD_2Cl_2, 270K) δ 156.64 ($C{=}\underline{C}Ph_2$), 155.59 (C_q, imidazole), 150.80 (C_q, Ph), 145.91 (C_q, Dipp), 145.69 (C_q, Dipp), 140.52 (C_q, Ph), 135.36 (C–Au), 133.07 (C_q, Dipp), 131.07 (CH_{arom}, Dipp), 129.74 (CH_{arom}, Ph), 128.86 (CH_{arom}, Ph), 128.41 (CH_{arom}, Ph), 128.24 (CH_{arom}, Ph), 127.46 (CH_{arom}, Ph), 126.60 (CH_{arom}, Ph), 125.27 (CH_{arom}, Dipp), 124.60 (CH_{arom}, Dipp), 123.01 ($CH_{imidazole}$), 29.72 ($\underline{C}H(CH_3)_2$, Dipp), 29.15 ($\underline{C}H(CH_3)_2$, Dipp), 26.29 (CH_3, Dipp), 26.22 (CH_3, Dipp), 22.69 (CH_3, Dipp), 22.30 (CH_3, Dipp).

HRMS (nanochip-ESI/LTQ-Orbitrap) *m/z*: $[M + Na]^+$ Calculated for $C_{41}H_{46}AuClN_2Na^+$ 821.2907; Found 821.2923.

3. NMR Data

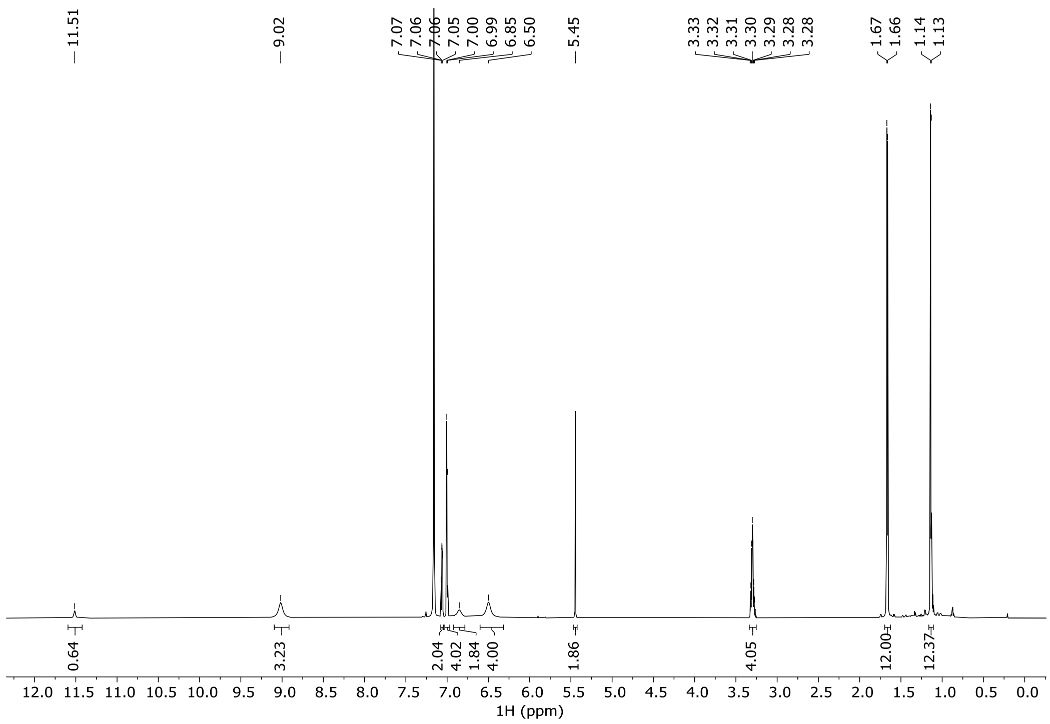

Figure S1. ${}^{1}H$ NMR (800 MHz, C_6D_6) spectrum of **2**.

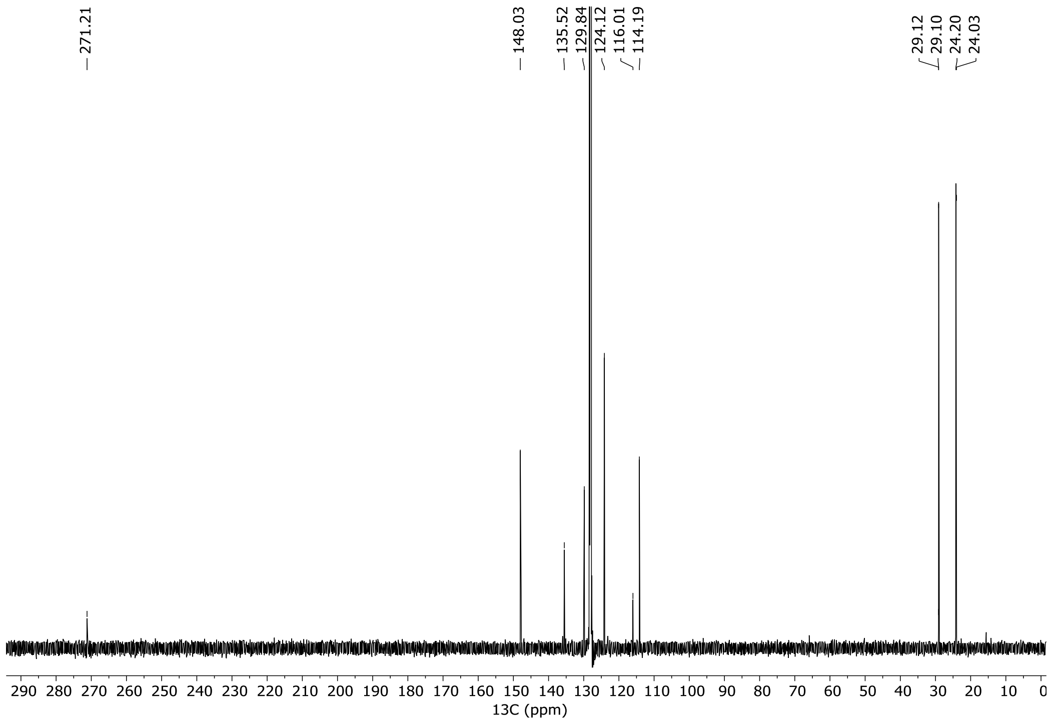

Figure S2. $\{{}^{1}H\}{}^{13}C$ NMR (201 MHz, C_6D_6) spectrum of **2**.

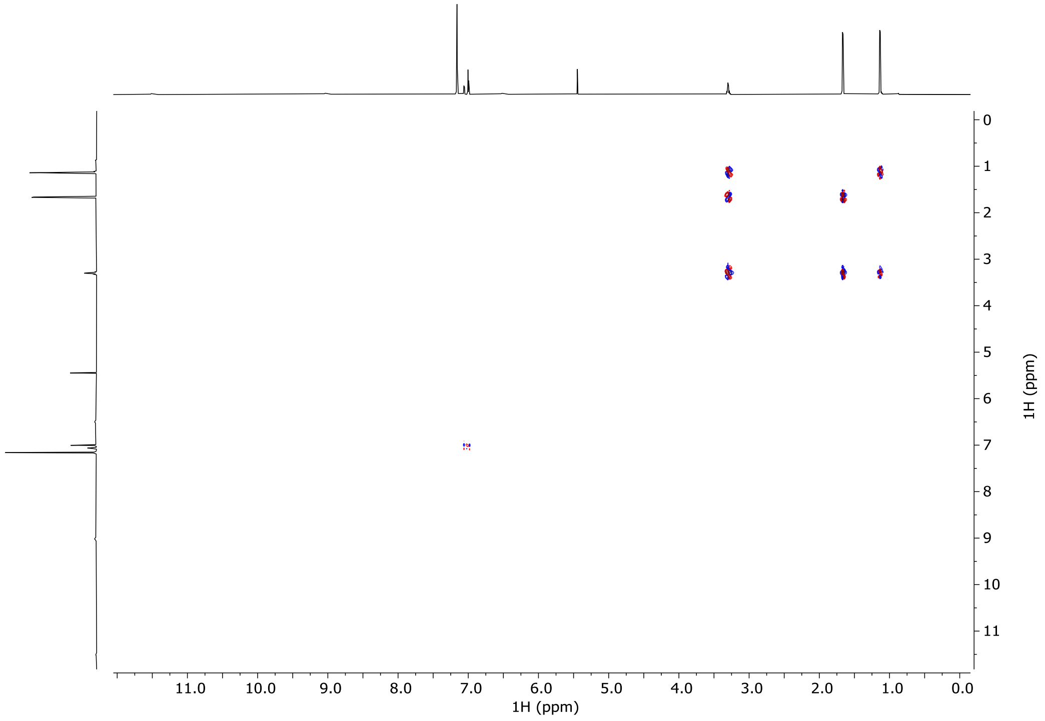

Figure S3. ^{1}H COSY NMR (C_6D_6) spectrum of **2**.

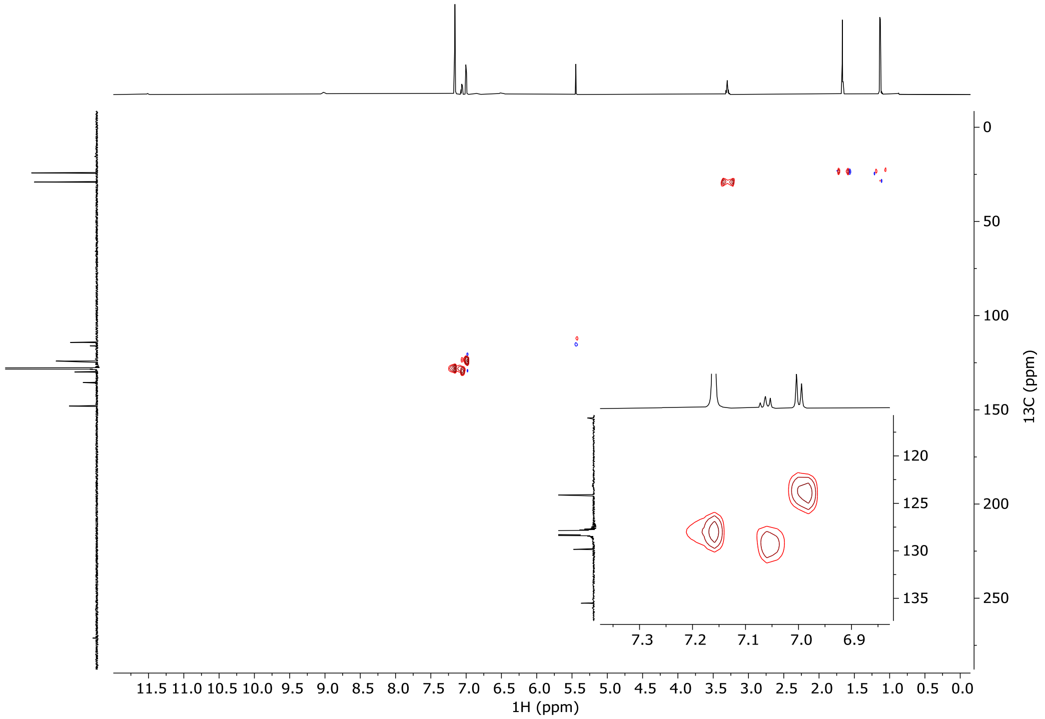

Figure S3. $^{1}H/^{13}C$ HSQC NMR (C_6D_6) spectrum of **2**.

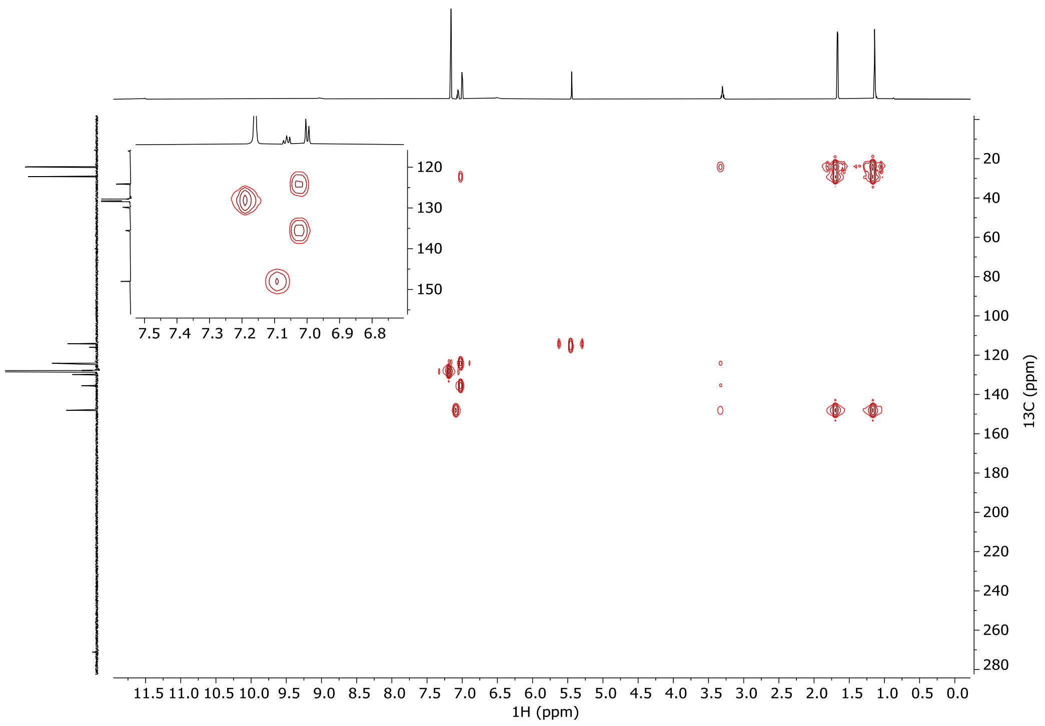

Figure S4. $^{1}H/^{13}C$ HMBC NMR (C_6D_6) spectrum of **2**.

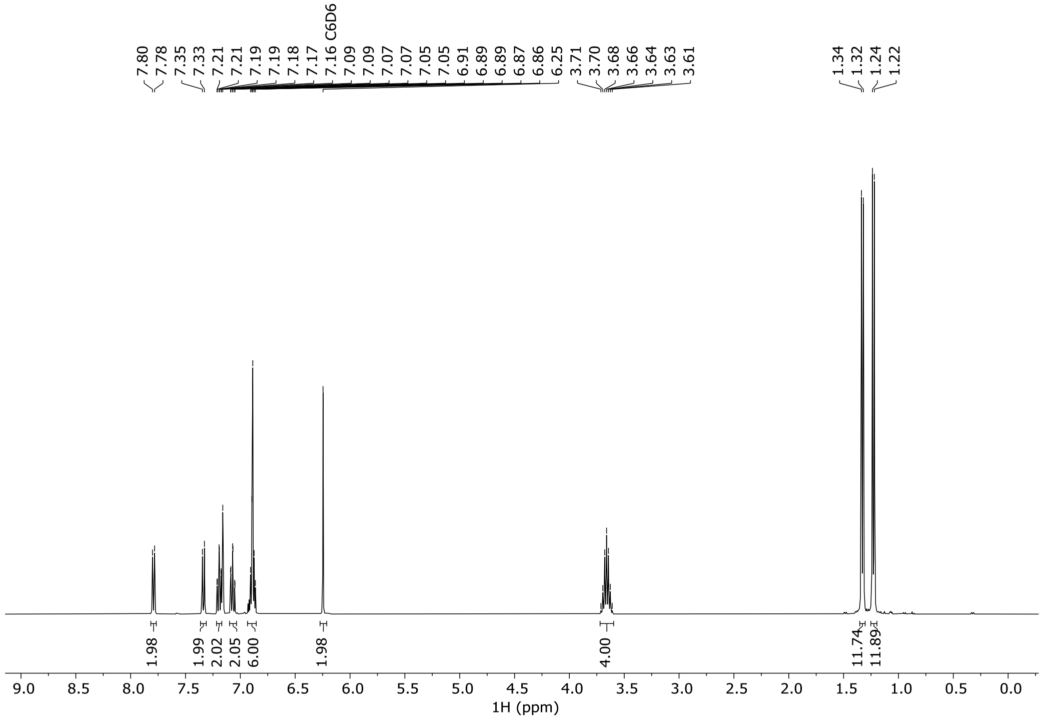

Figure S5. ^{1}H NMR (400 MHz, C_6D_6) spectrum of **3**.

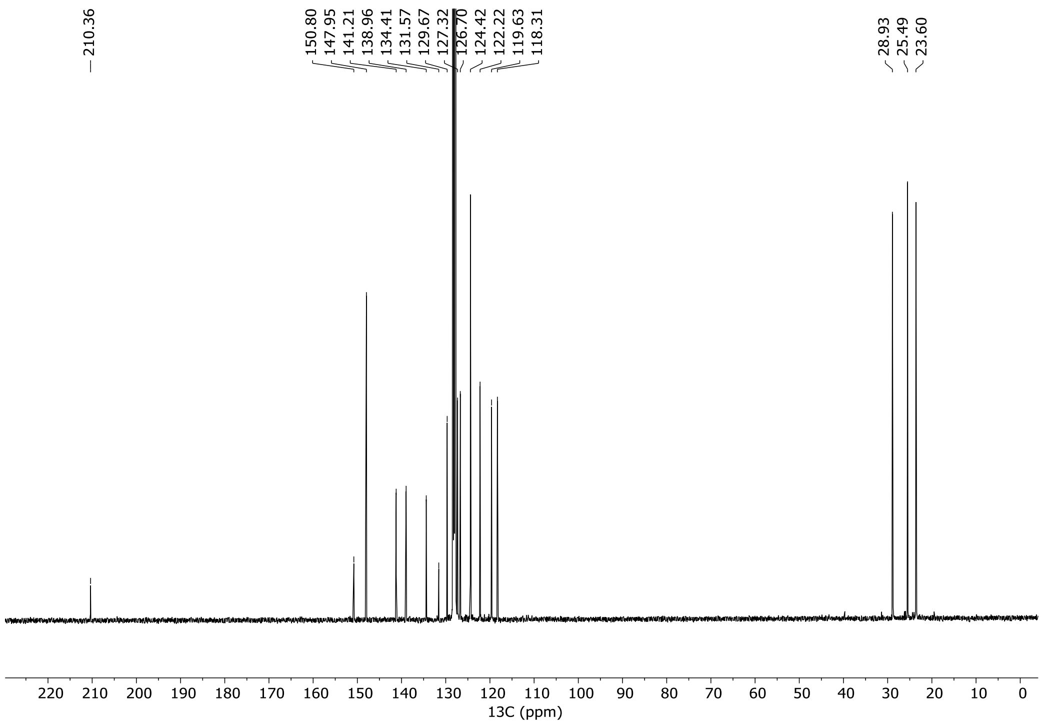

Figure S6. {^{1}H}^{13}C NMR (101 MHz, C_6D_6) spectrum of **3**.

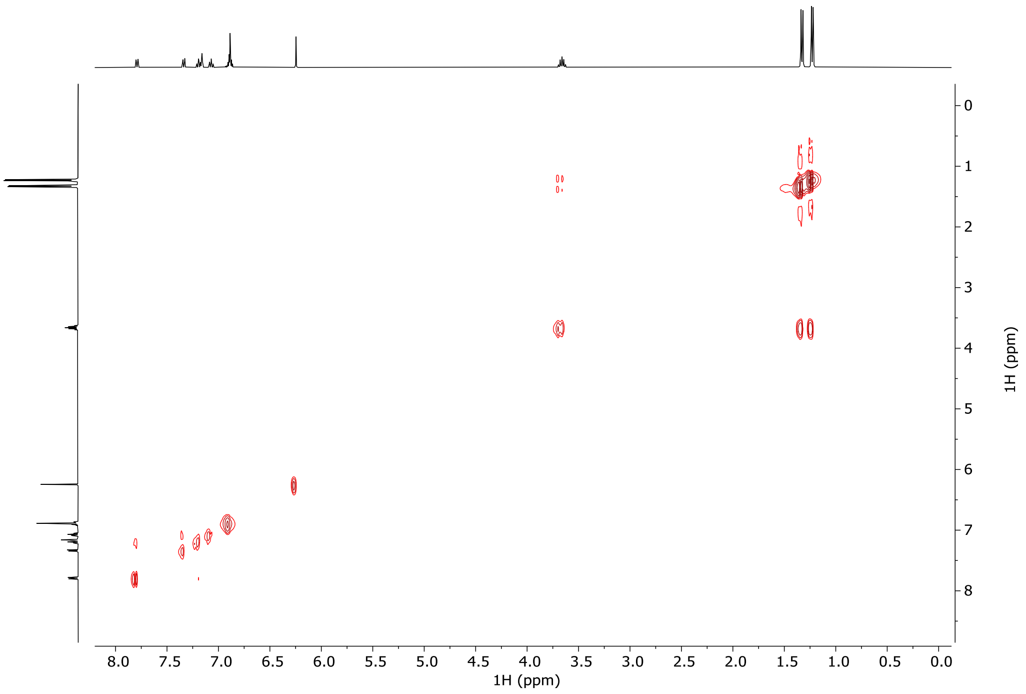

Figure S7. ^{1}H COSY NMR (C_6D_6) spectrum of **3**.

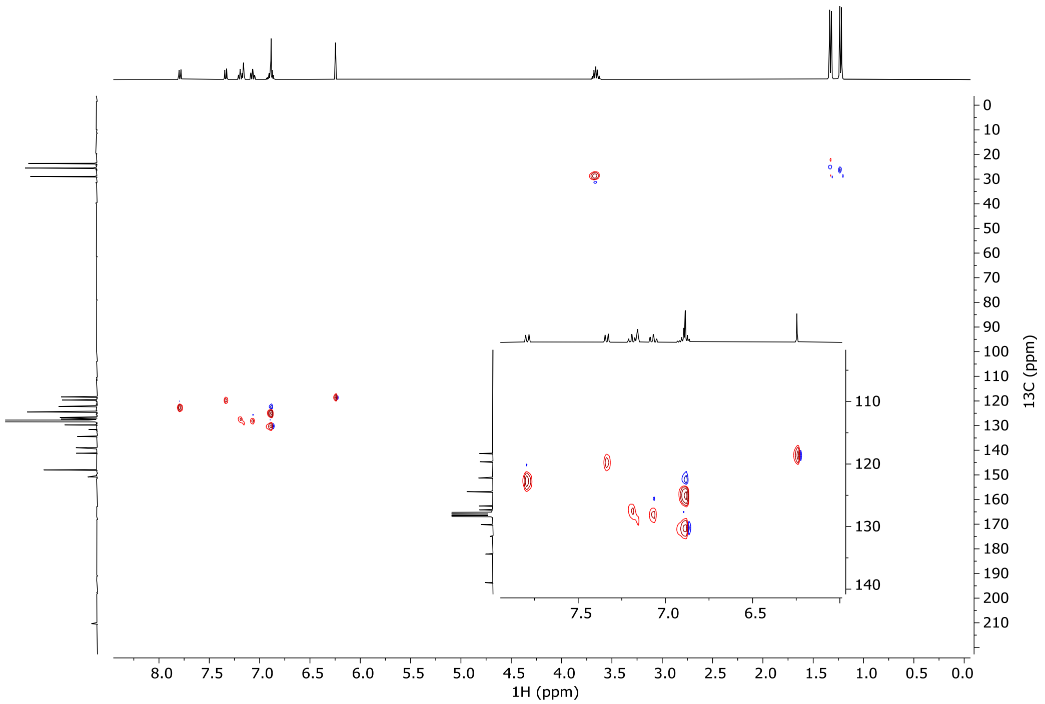

Figure S8. ^{1}H/^{13}C HSQC NMR (C_6D_6) spectrum of **3**.

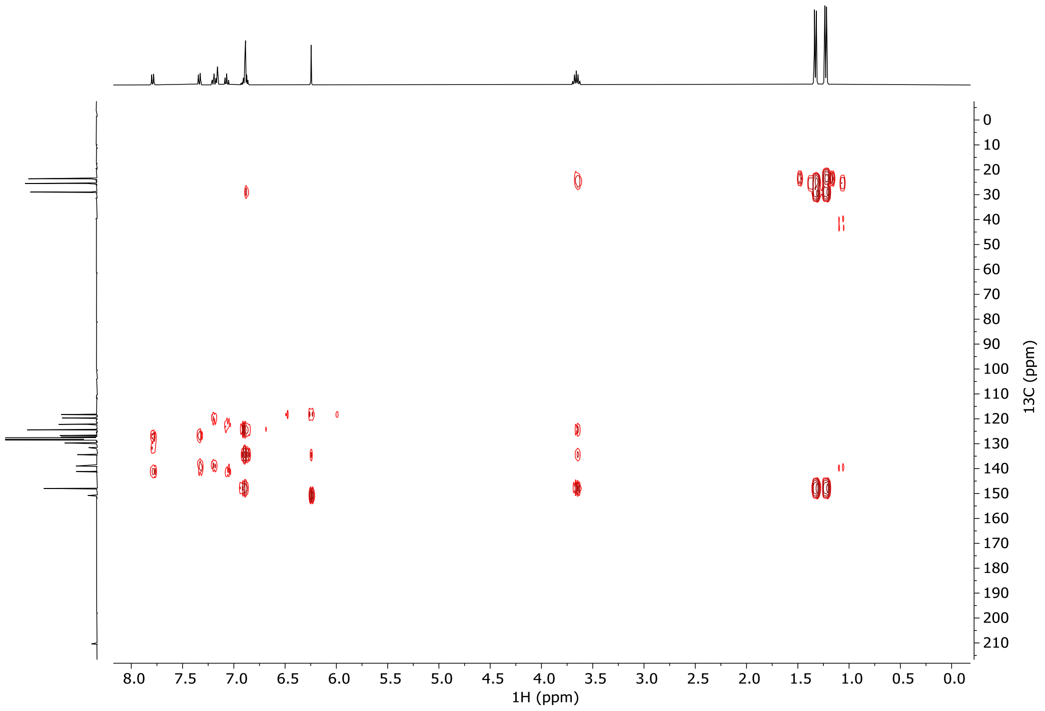

Figure S9. ^{1}H/^{13}C HMBC NMR (C_6D_6) spectrum of **3**.

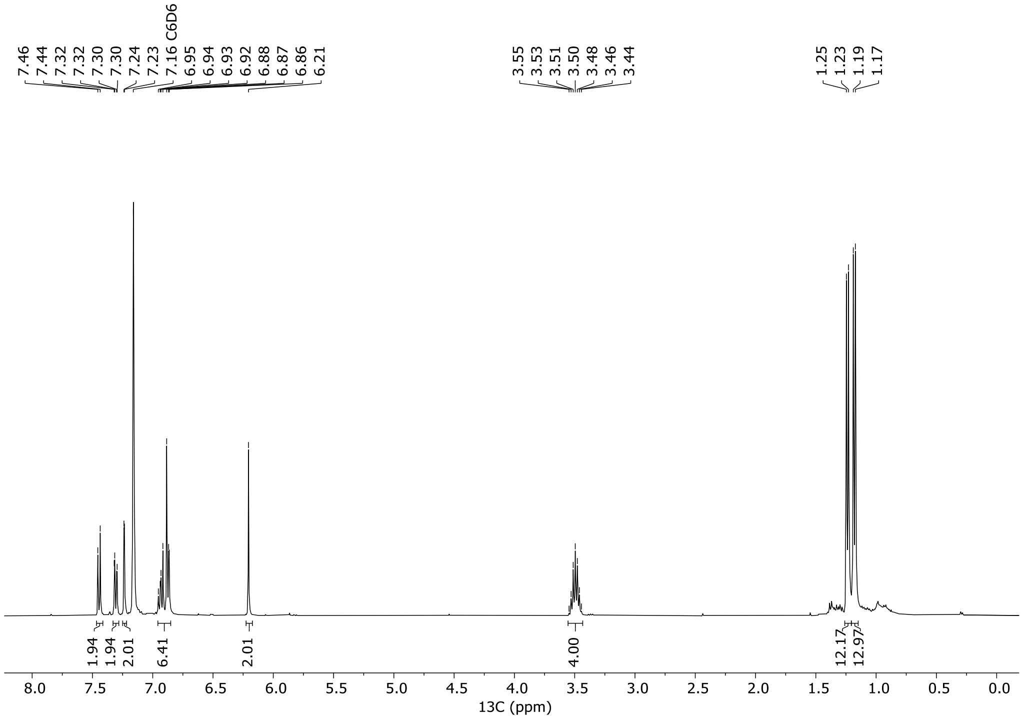

Figure S10. ^{1}H NMR (400 MHz, C_6D_6) spectrum of **4**.

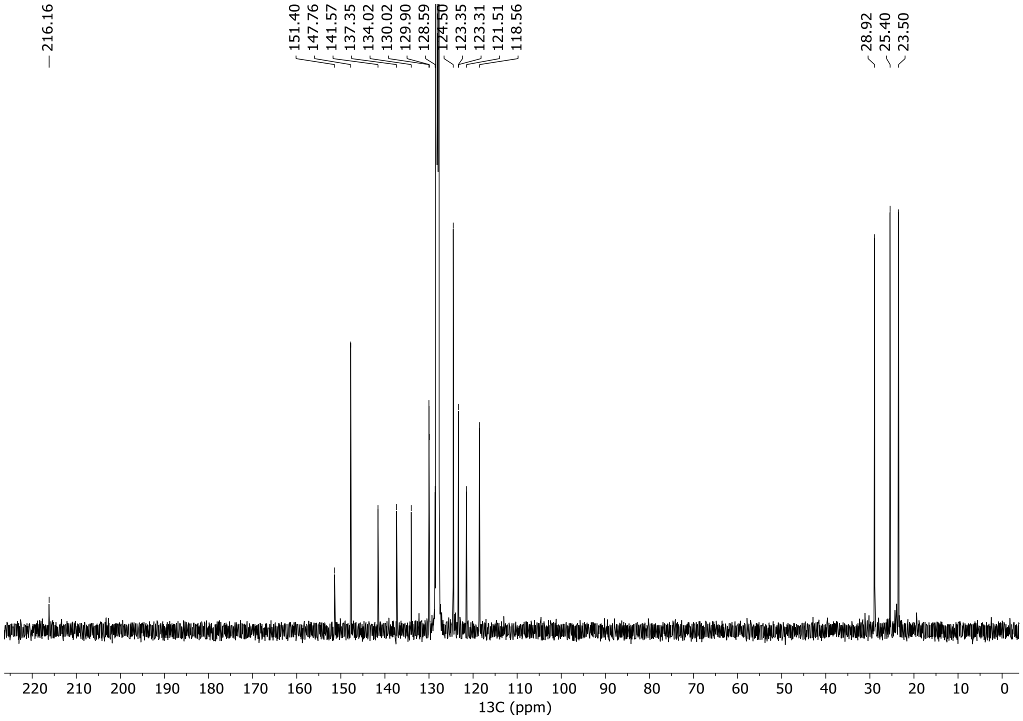

Figure S11. {^{1}H}^{13}C NMR (101 MHz, C_6D_6) spectrum of **4**.

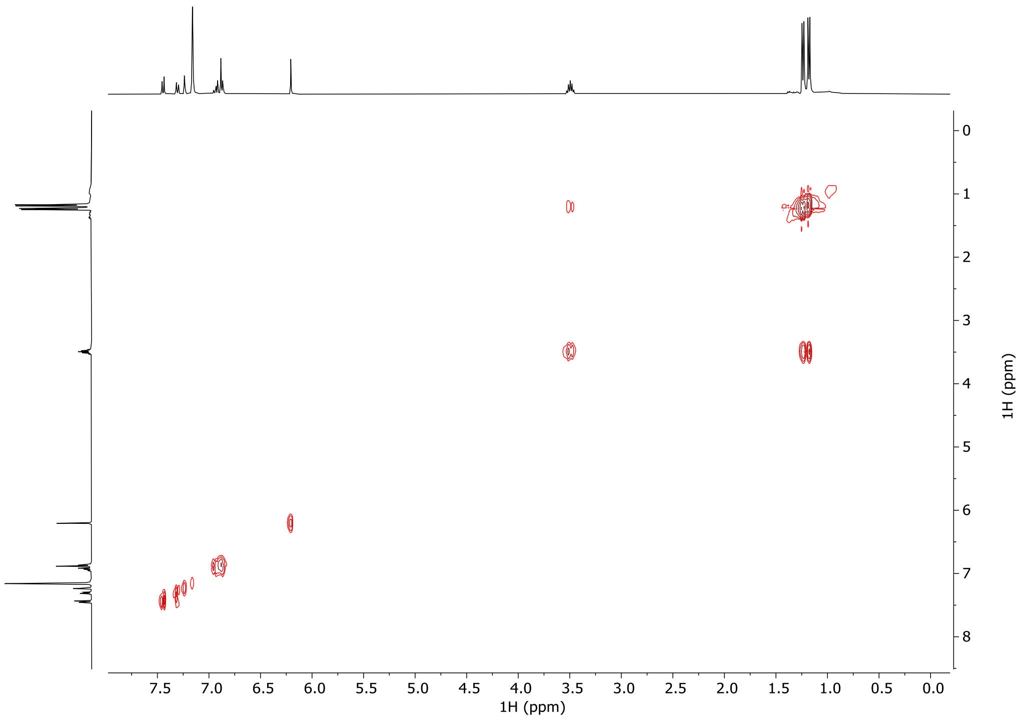

Figure S12. ^{1}H COSY NMR (C_6D_6) spectrum of **4**.

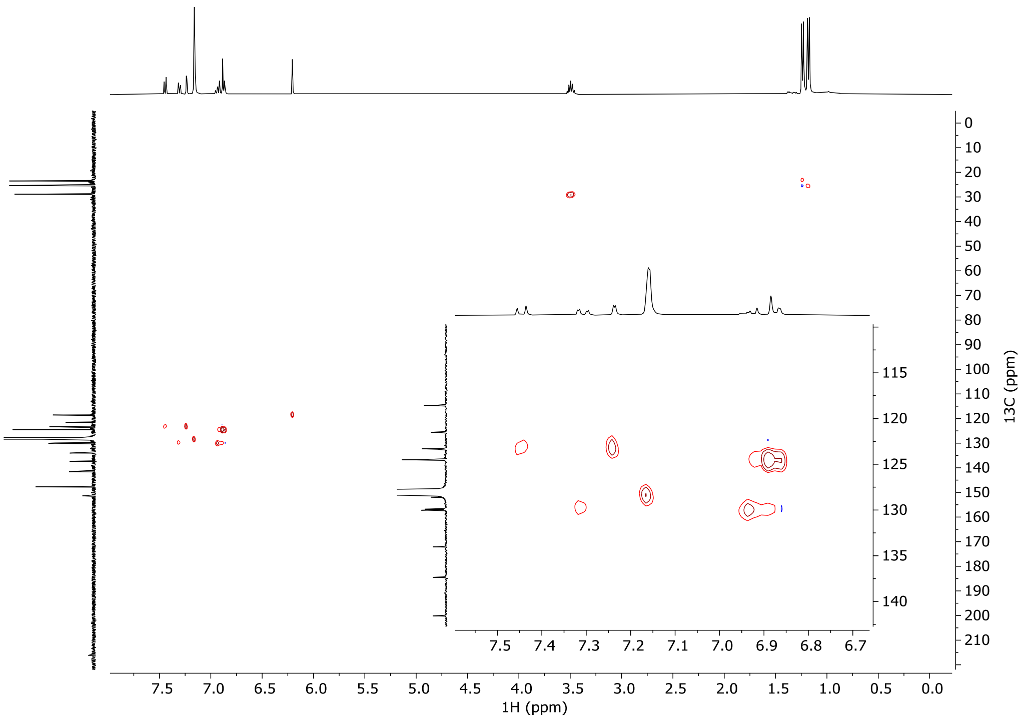

Figure S13. ^{1}H/^{13}C HSQC NMR (C_6D_6) spectrum of **4**.

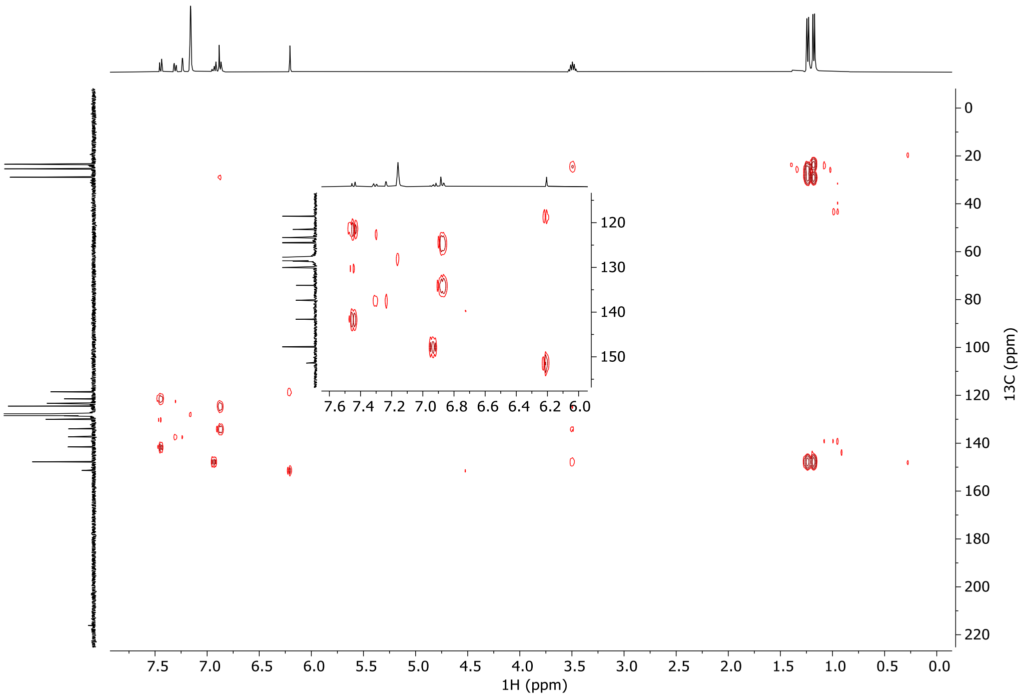

Figure S14. $^{1}H/^{13}C$ HMBC NMR (C_6D_6) spectrum of **4**.

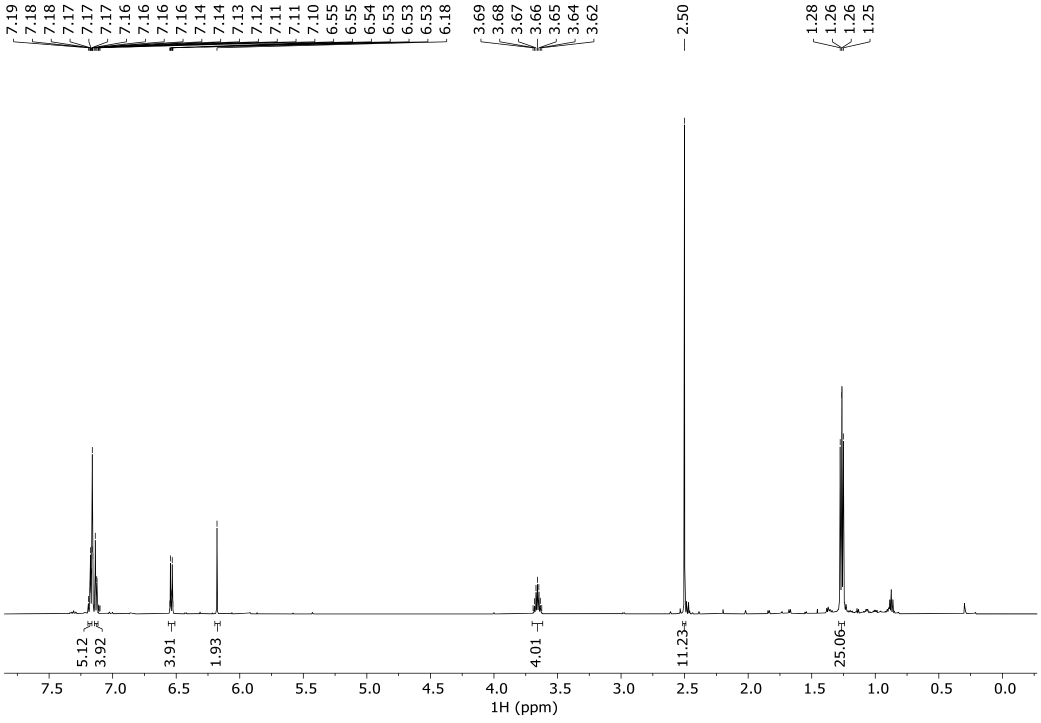

Figure S15. ^{1}H NMR (600 MHz, C_6D_6) spectrum of **5**.

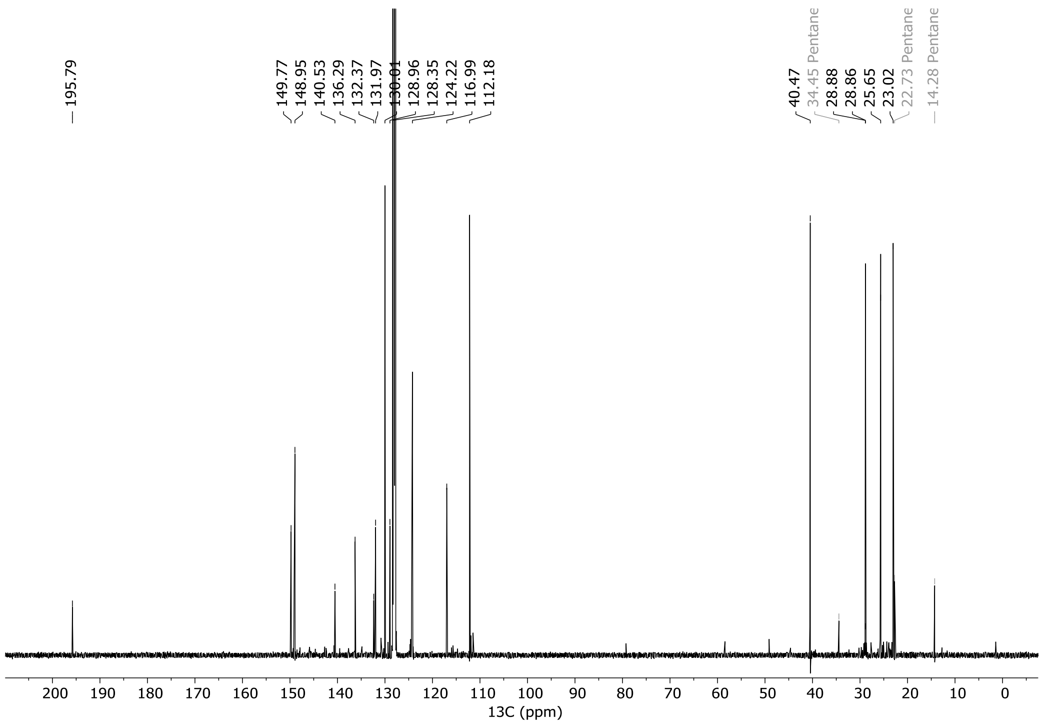

Figure S16. {^{1}H}^{13}C NMR (151 MHz, C_6D_6) spectrum of **5**.

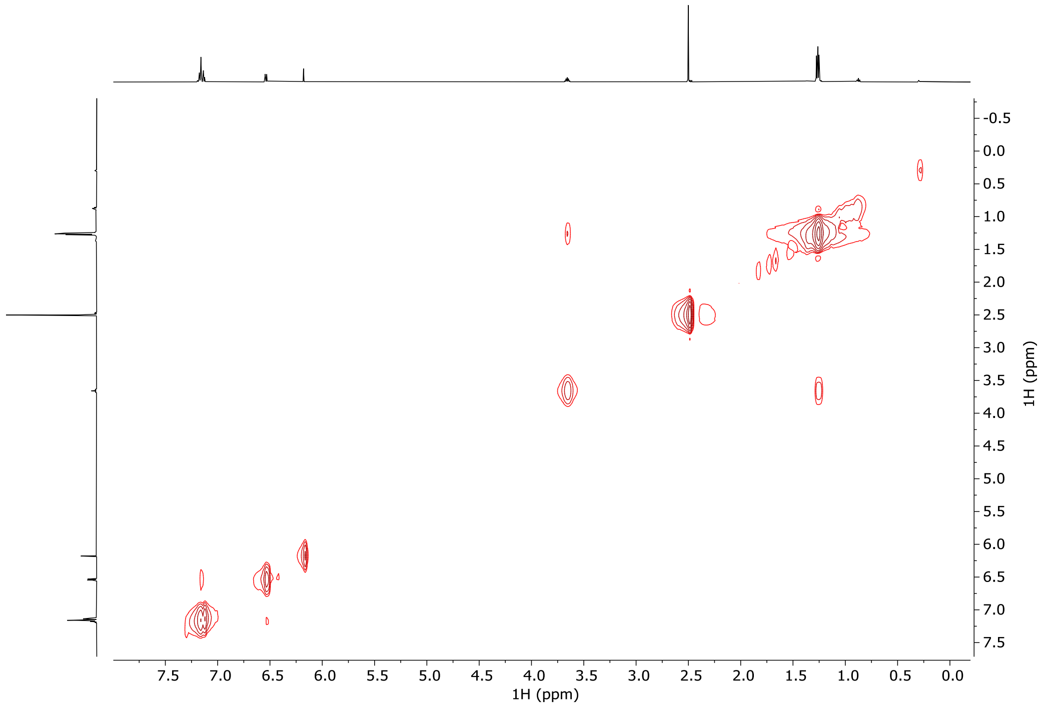

Figure S17. ^{1}H COSY NMR (C_6D_6) spectrum of **5**.

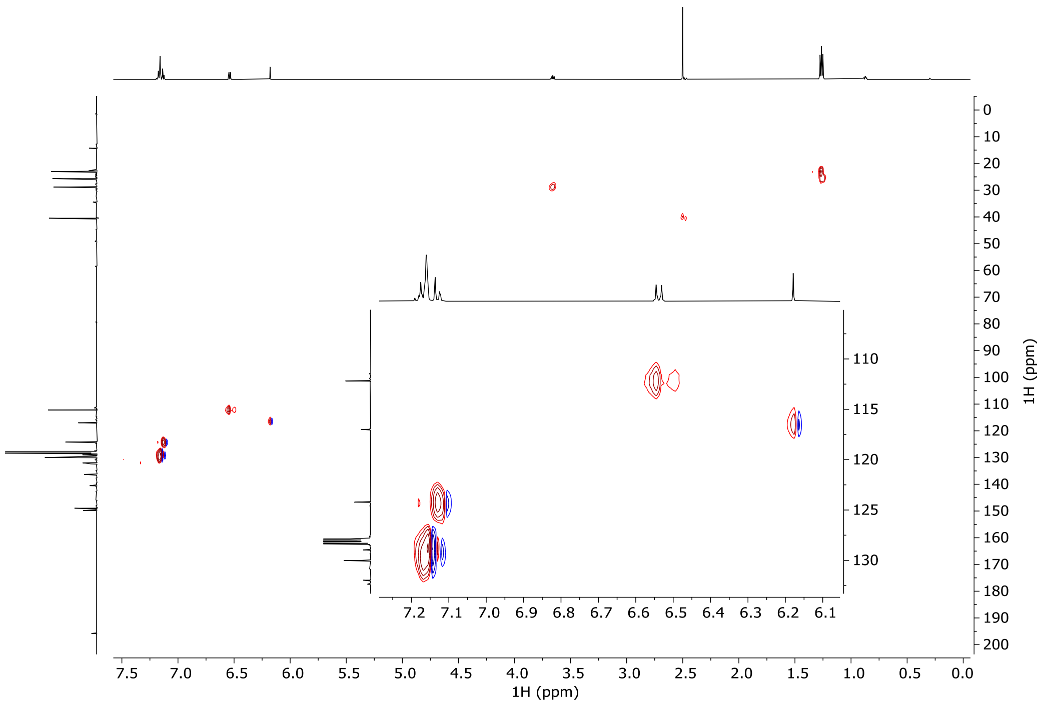

Figure S18. $^{1}H/^{13}C$ HSQC NMR (C_6D_6) spectrum of **5**.

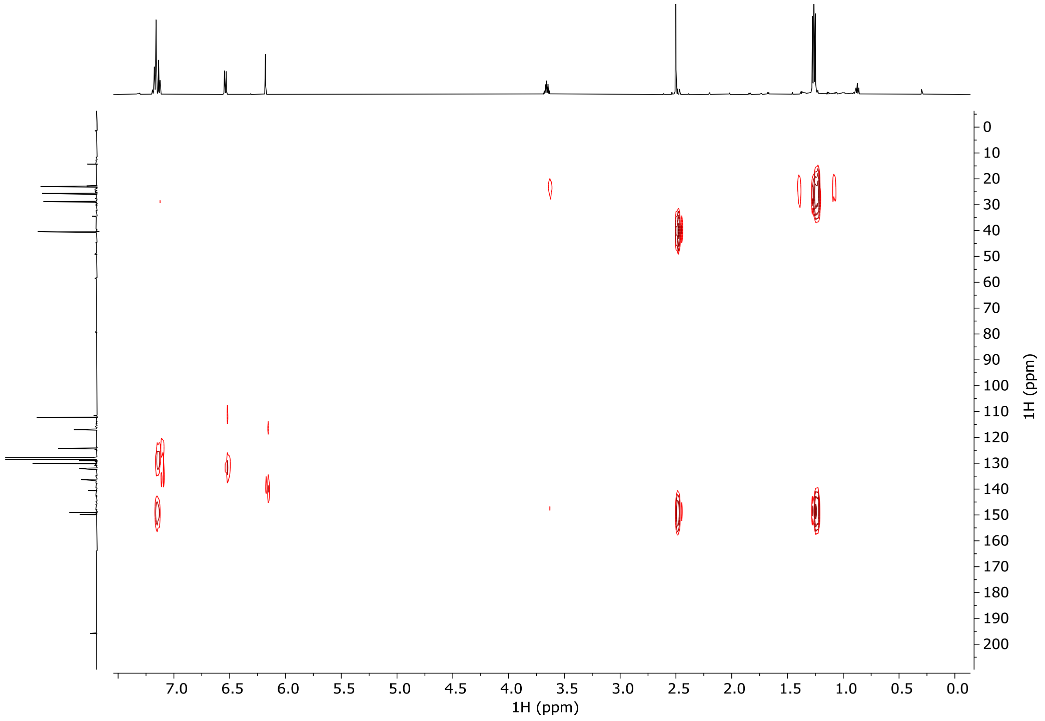

Figure S19. $^{1}H/^{13}C$ HMBC NMR (C_6D_6) spectrum of **5**.

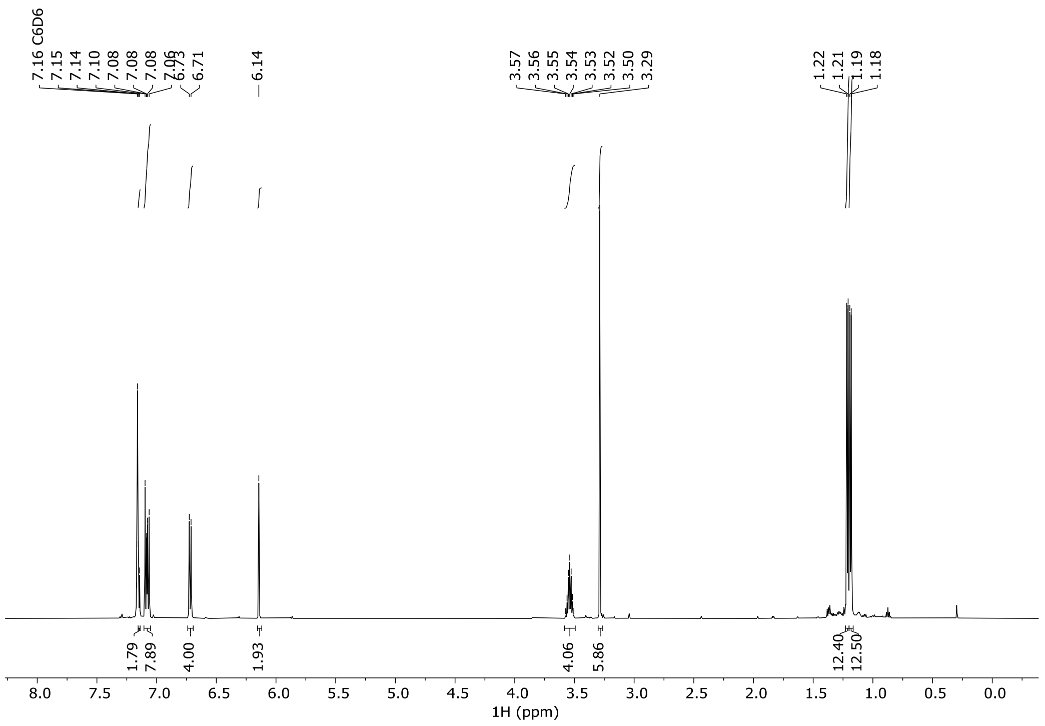

Figure S20. ^{1}H NMR (600 MHz, C_6D_6) spectrum of **6**.

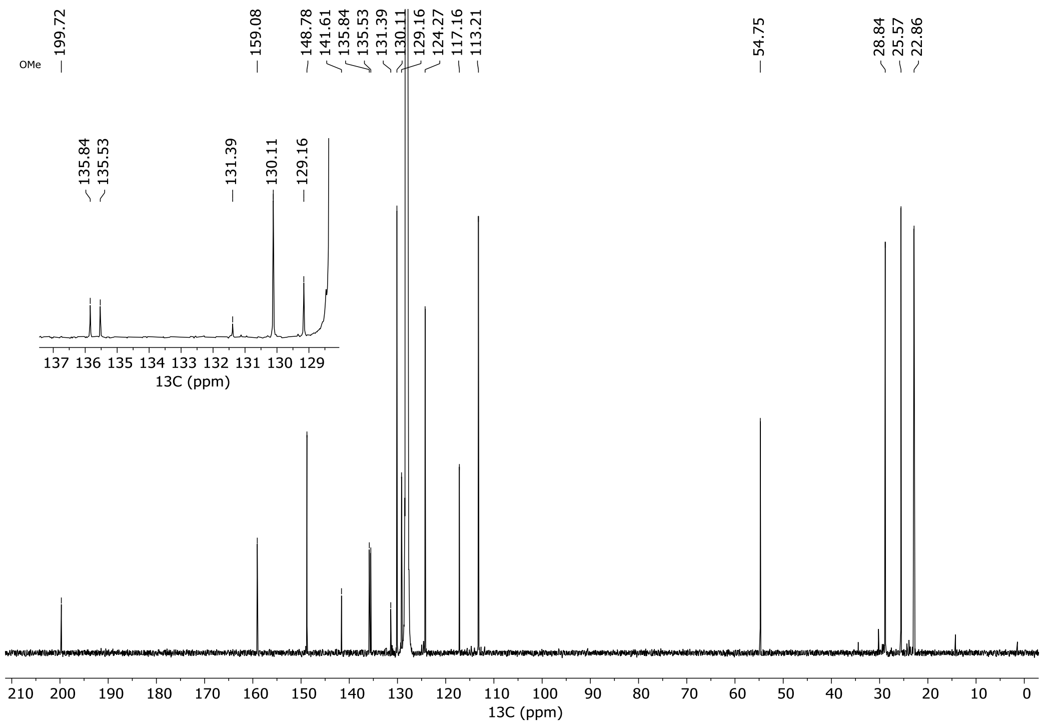

Figure S21. $\{^{1}H\}^{13}C$ NMR (151 MHz, C_6D_6) spectrum of **6**.

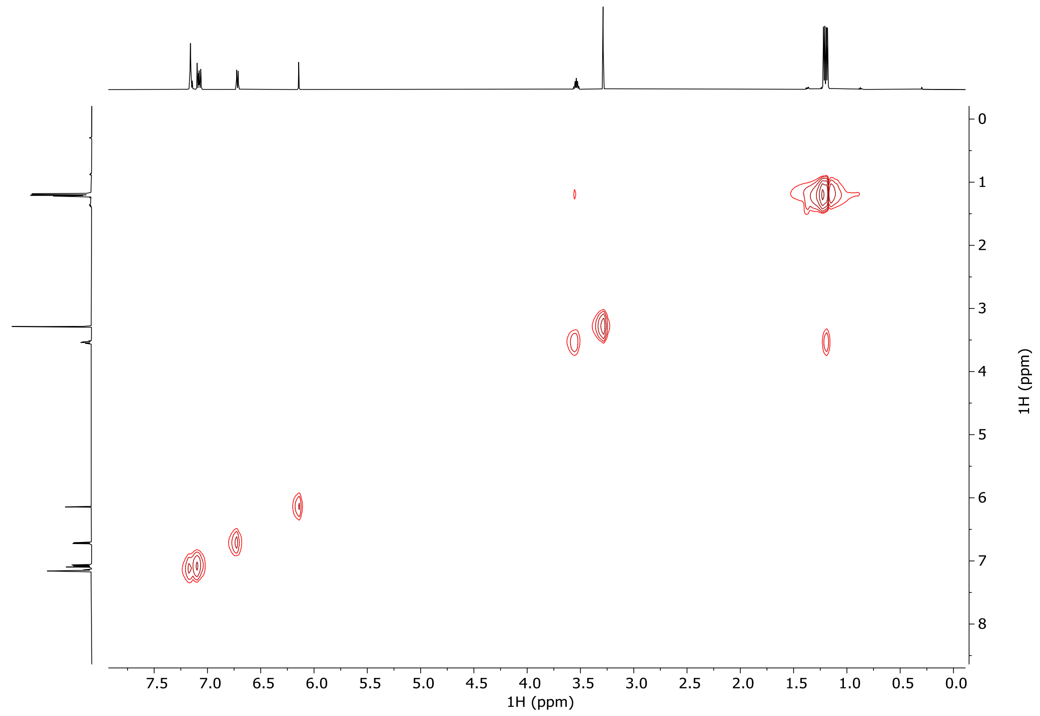

Figure S22. ^{1}H COSY NMR (C_6D_6) spectrum of **6**.

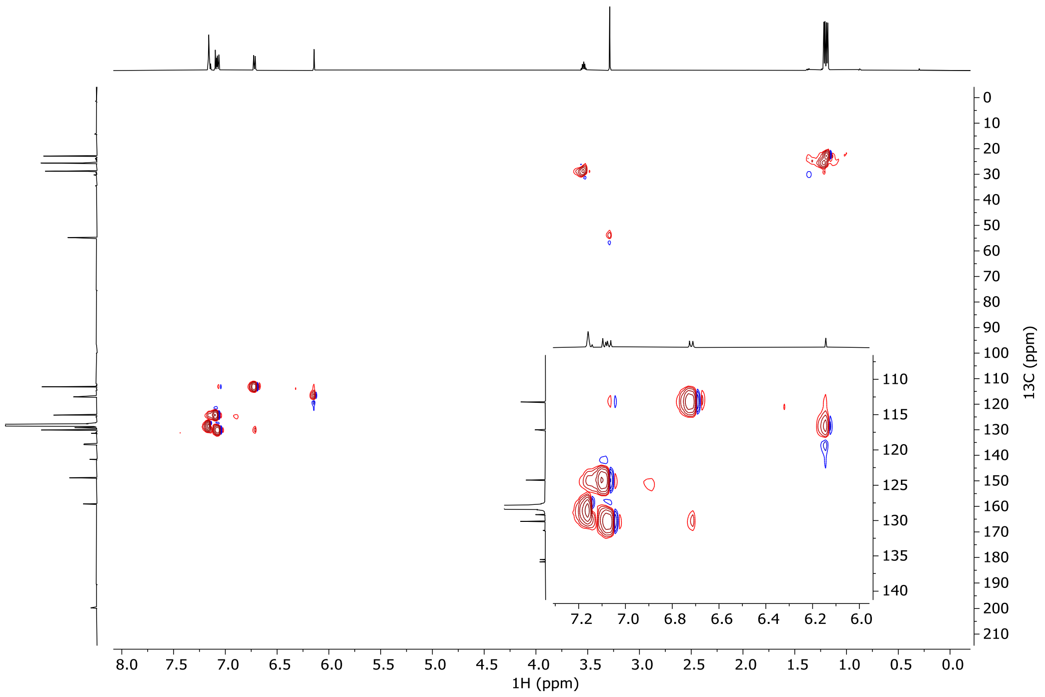

Figure S23. $^{1}H/^{13}C$ HSQC NMR (C_6D_6) spectrum of **6**.

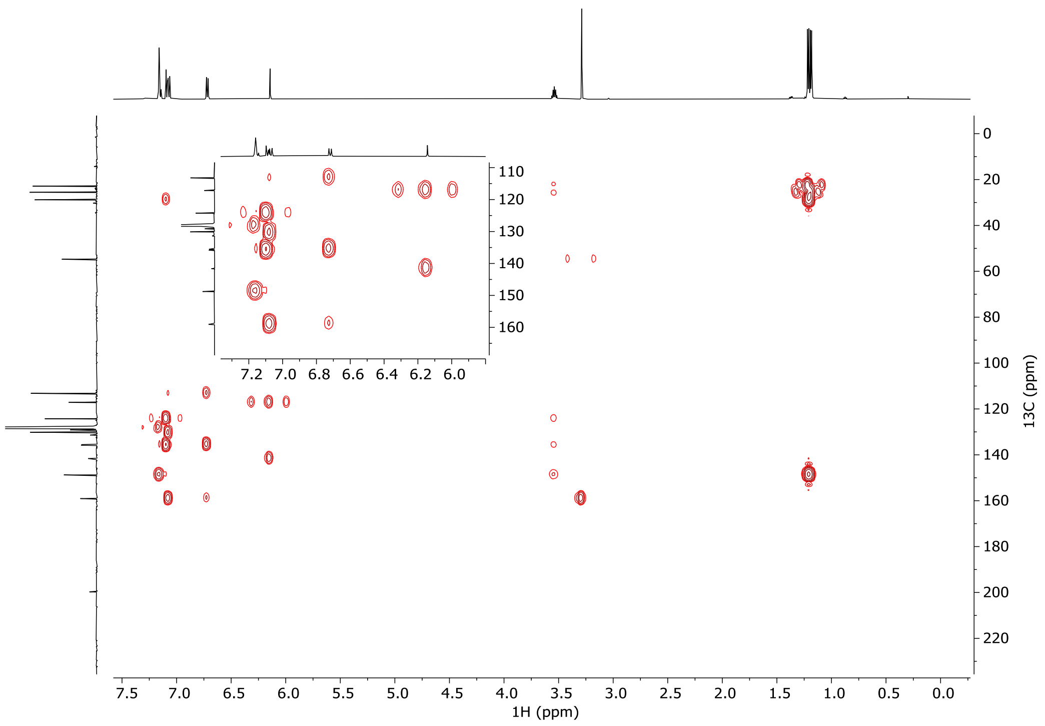

Figure S24. $^{1}H/^{13}C$ HMBC NMR (C_6D_6) spectrum of **6**.

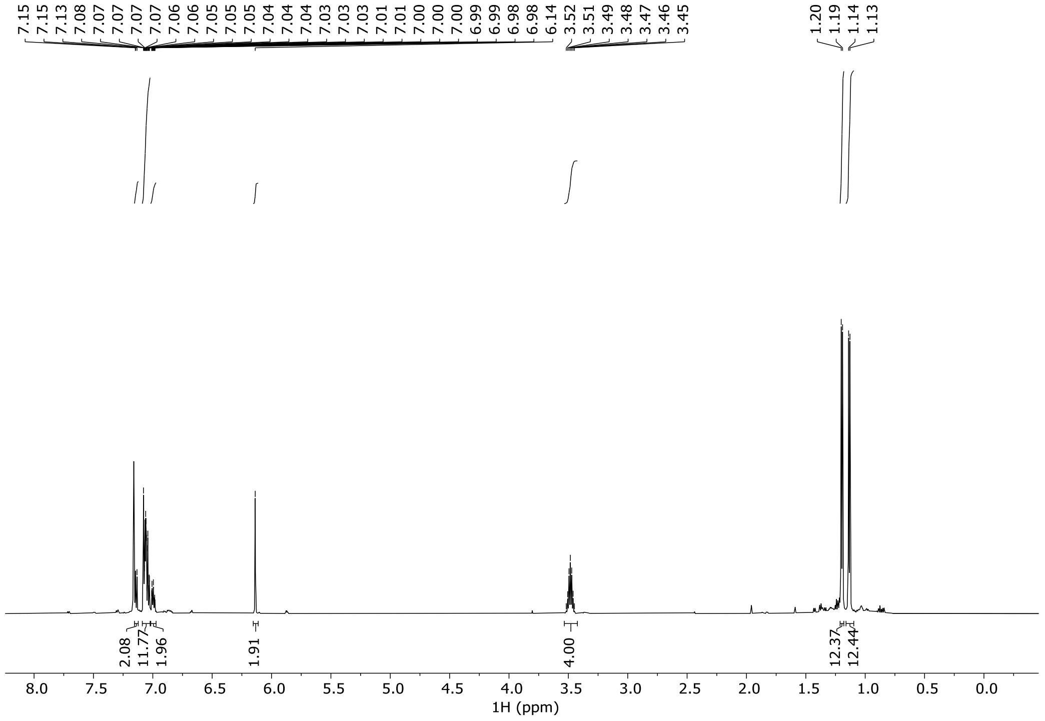

Figure S25. ^{1}H NMR (600 MHz, C_6D_6) spectrum of **7**.

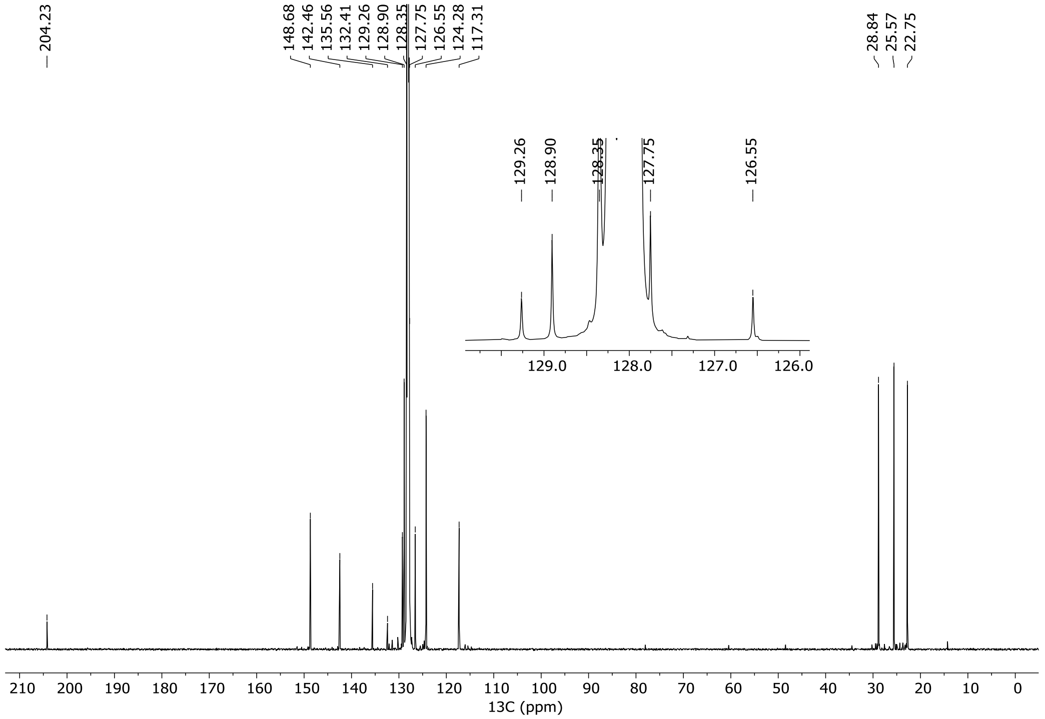

Figure S26. {^{1}H}^{13}C NMR (151 MHz, C_6D_6) spectrum of **7**.

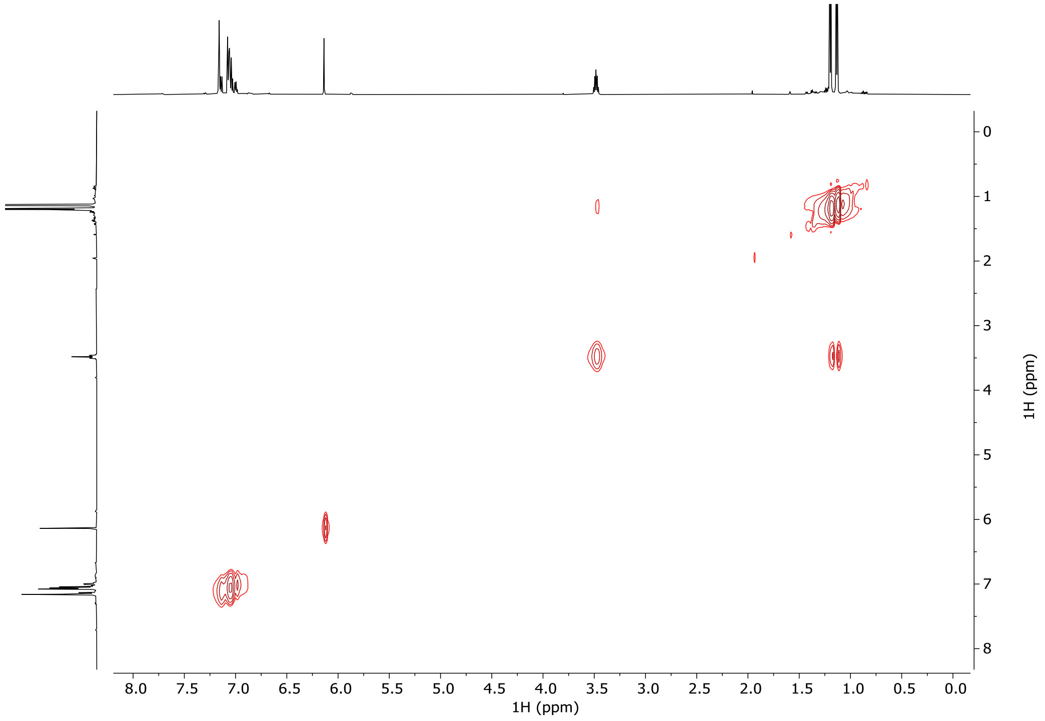

Figure S27. ^{1}H COSY NMR (C_6D_6) spectrum of **7**.

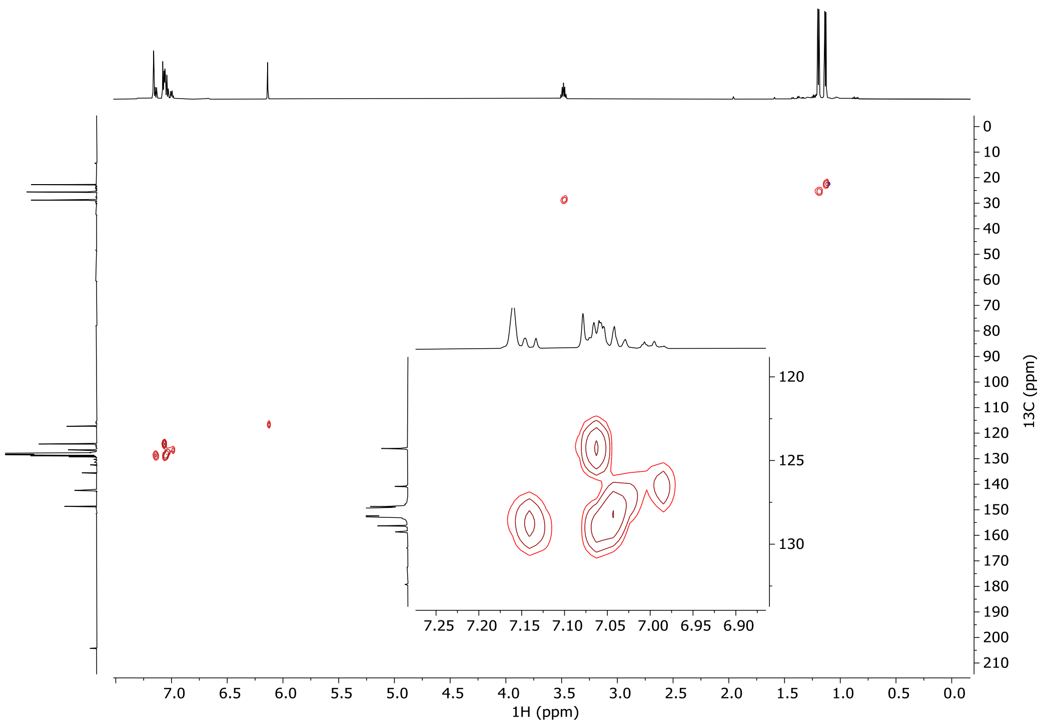

Figure S28. $^{1}H/^{13}C$ HSQC NMR (C_6D_6) spectrum of **7**.

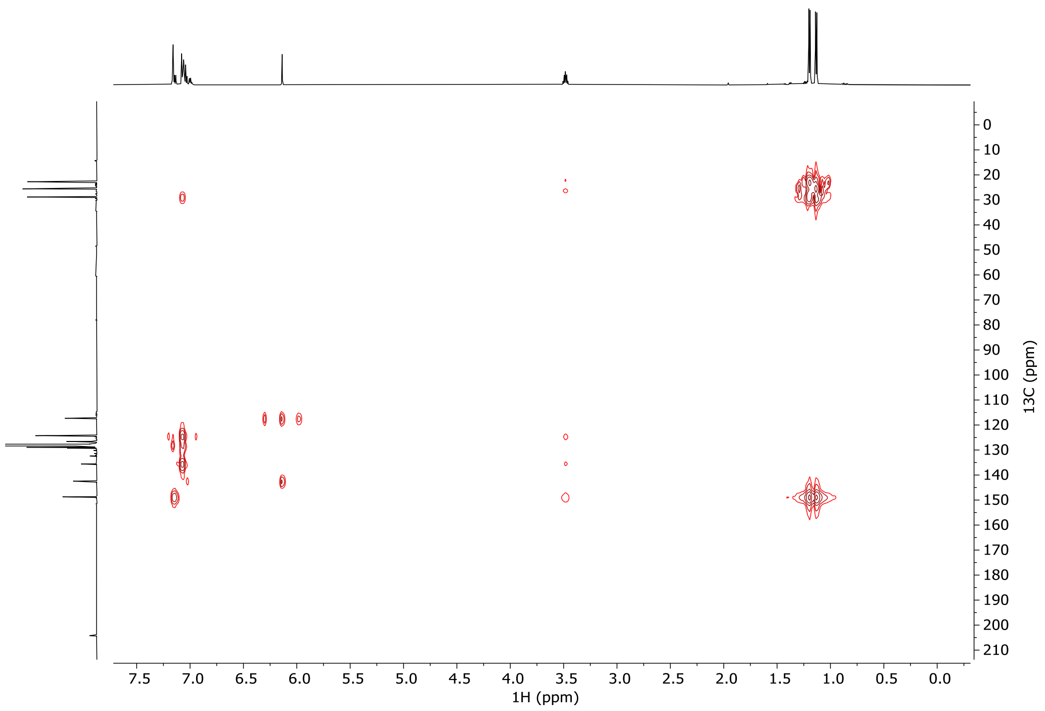

Figure S29. $^{1}H/^{13}C$ HMBC NMR (C_6D_6) spectrum of **7**.

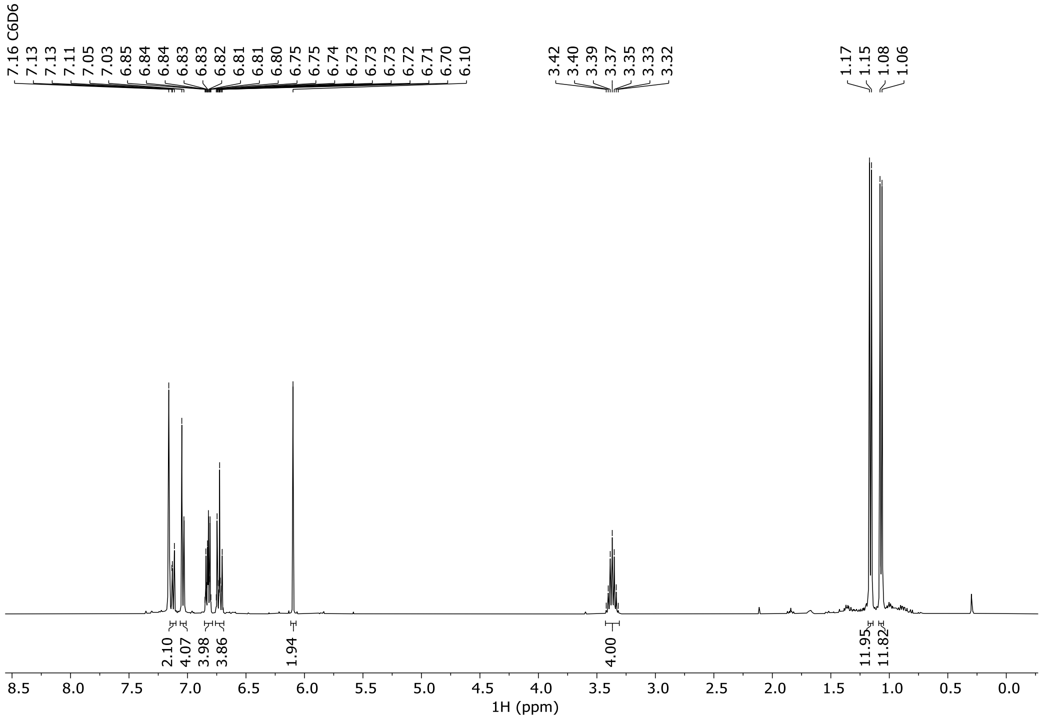

Figure S30. ^{1}H NMR (400 MHz, C_6D_6) spectrum of **8**.

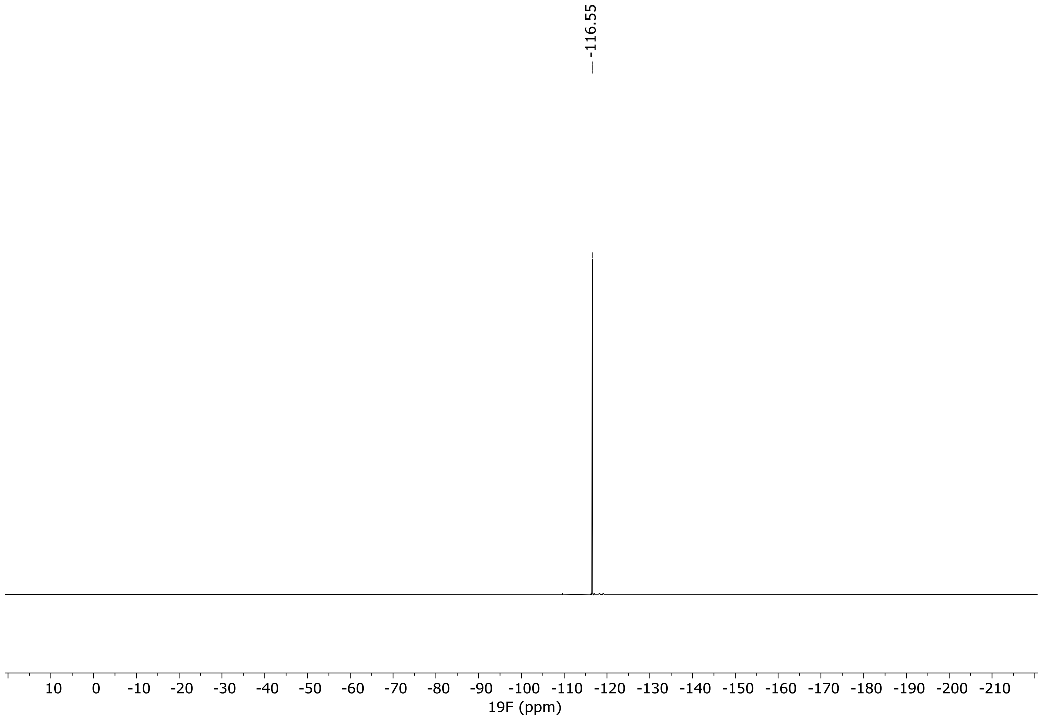

Figure S31. ^{19}F NMR (376 MHz, C_6D_6) spectrum of **8**.

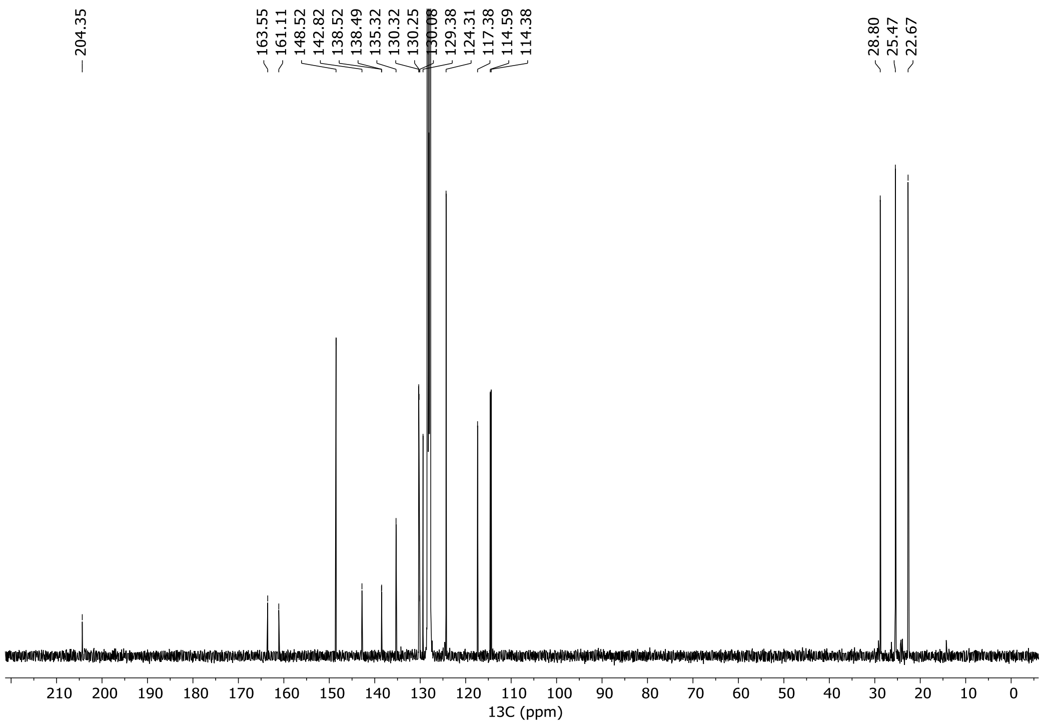

Figure S32. {^{1}H}^{13}C NMR (101 MHz, C_6D_6) spectrum of **8**.

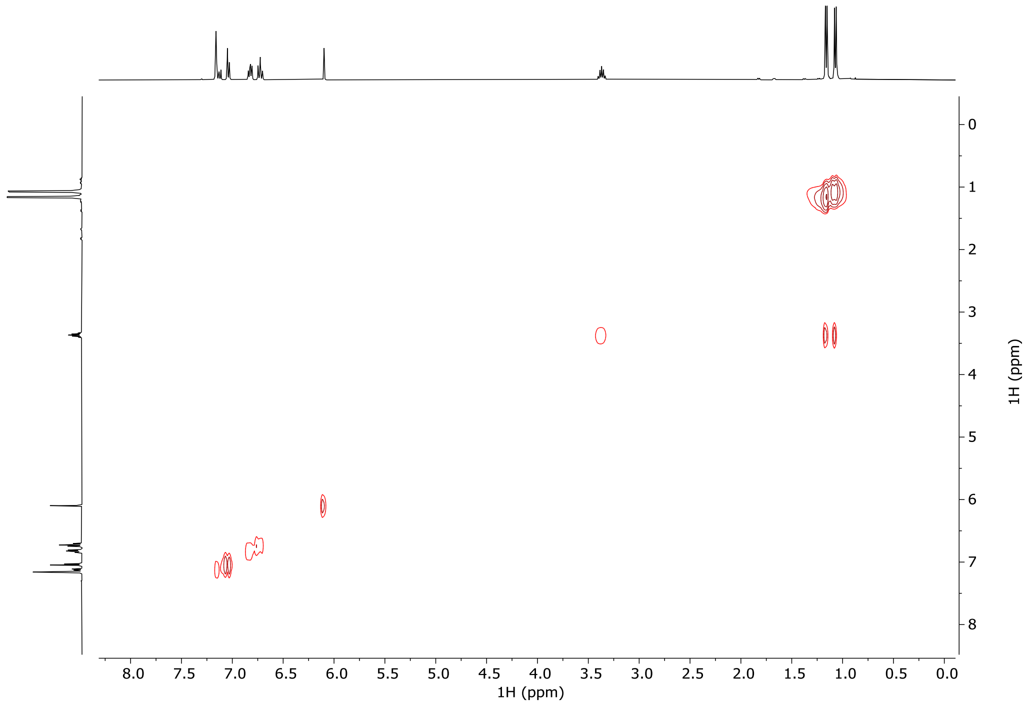

Figure S33. ^{1}H COSY NMR (C_6D_6) spectrum of **8**.

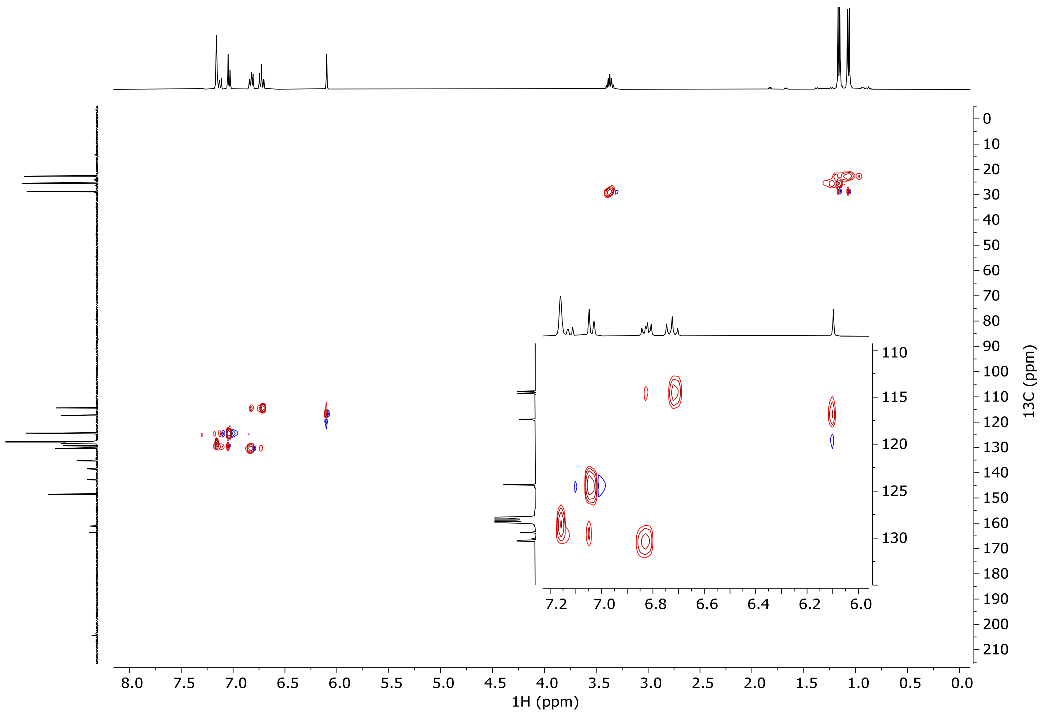

Figure S34. $^{1}H/^{13}C$ HSQC NMR (C_6D_6) spectrum of **8**.

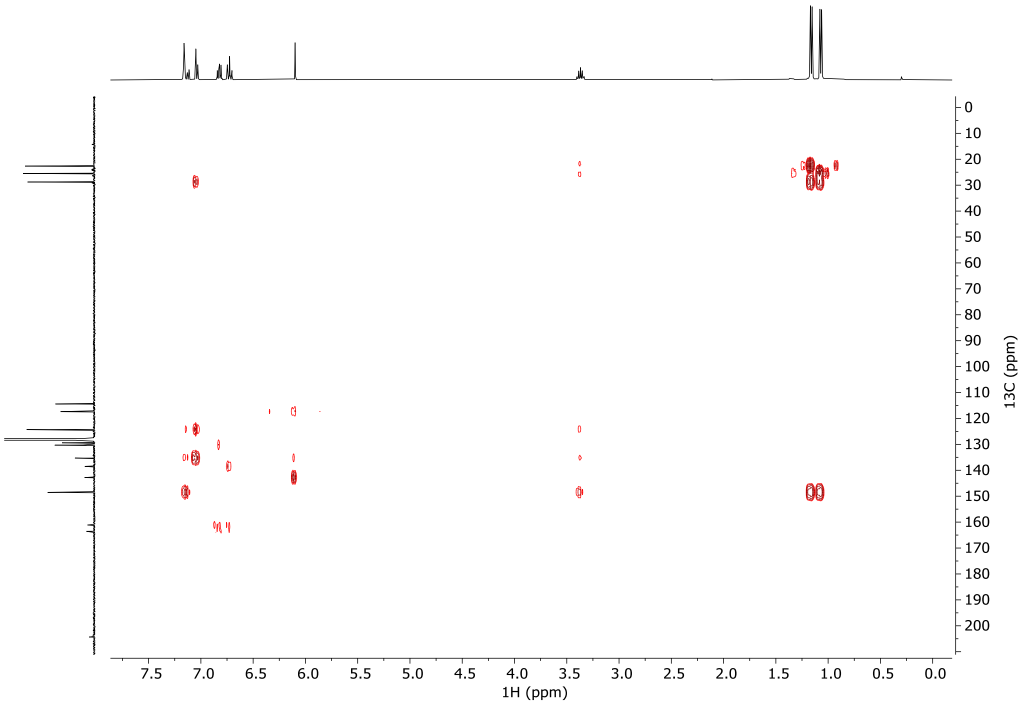

Figure S35. $^{1}H/^{13}C$ HMBC NMR (C_6D_6) spectrum of **8**.

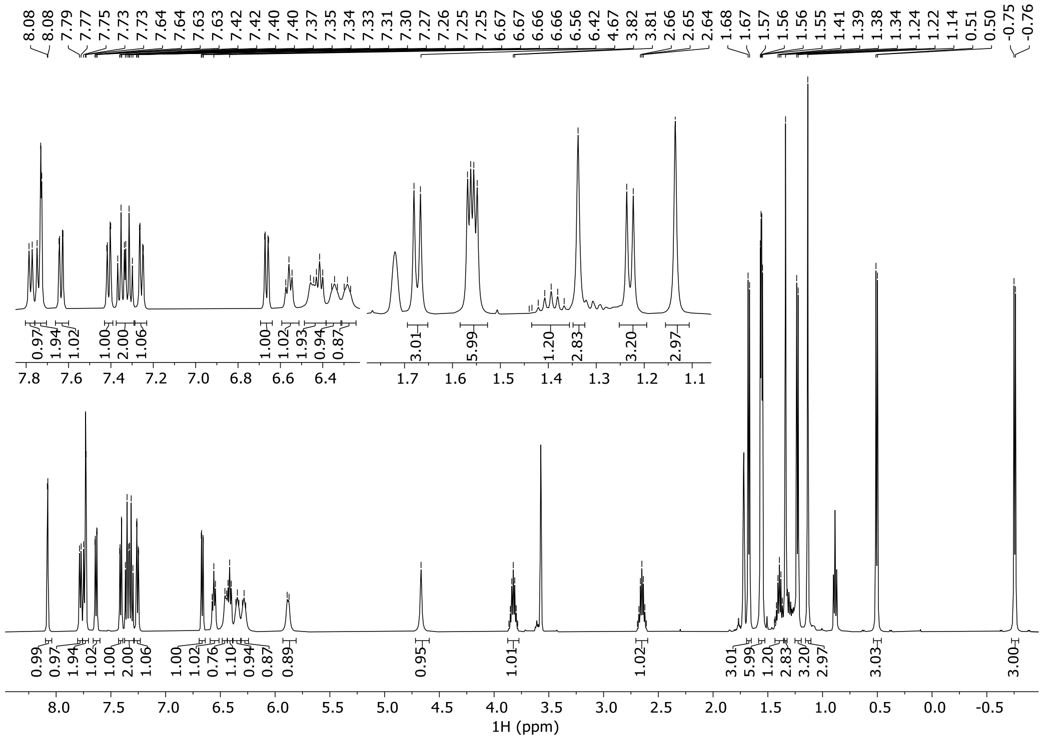

Figure S36. ^{1}H NMR (500 MHz, d_8-THF) spectrum of **9**.

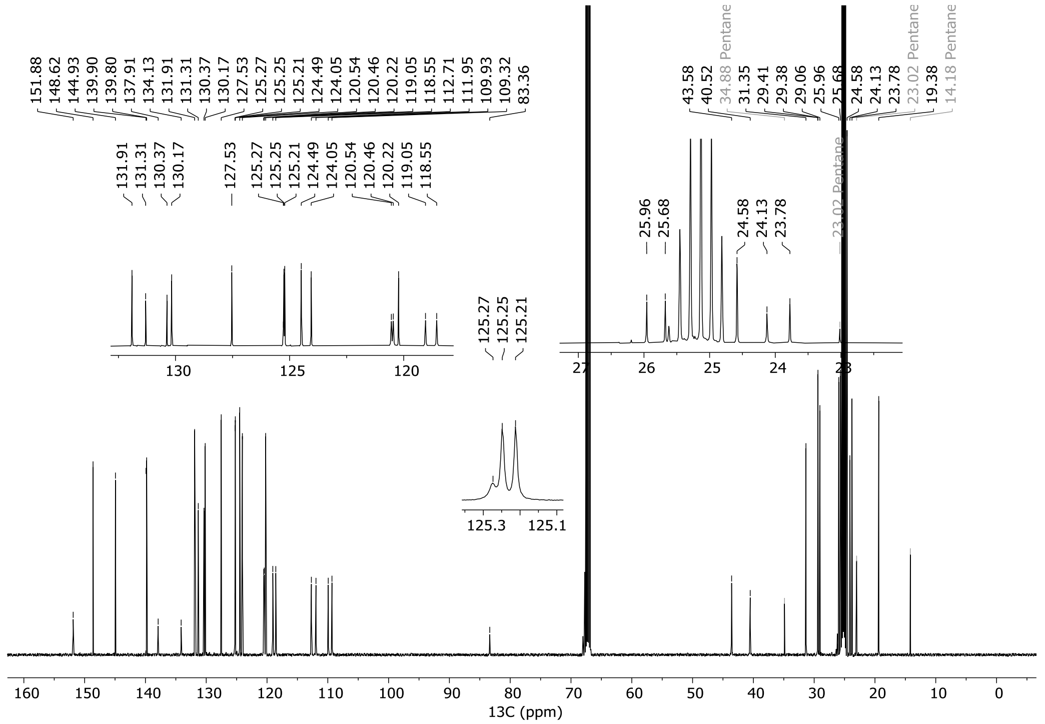

Figure S37. {^{1}H}^{13}C NMR (126 MHz, d_8-THF) spectrum of **9**.

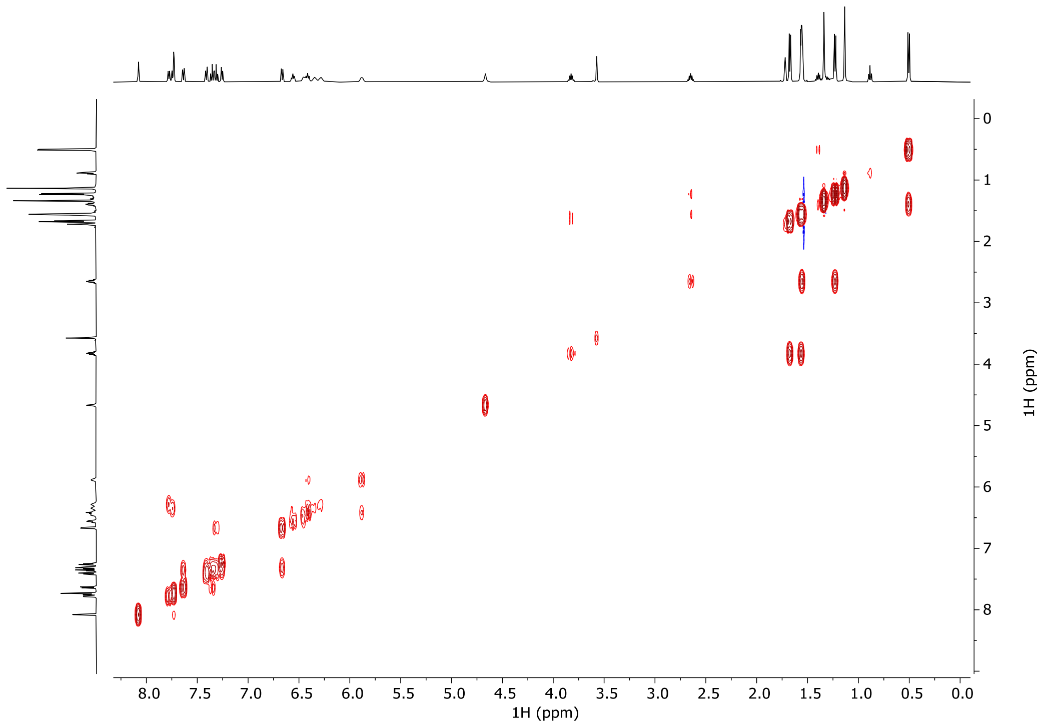

Figure S38. ^{1}H COSY NMR (d_8-THF) spectrum of **9**.

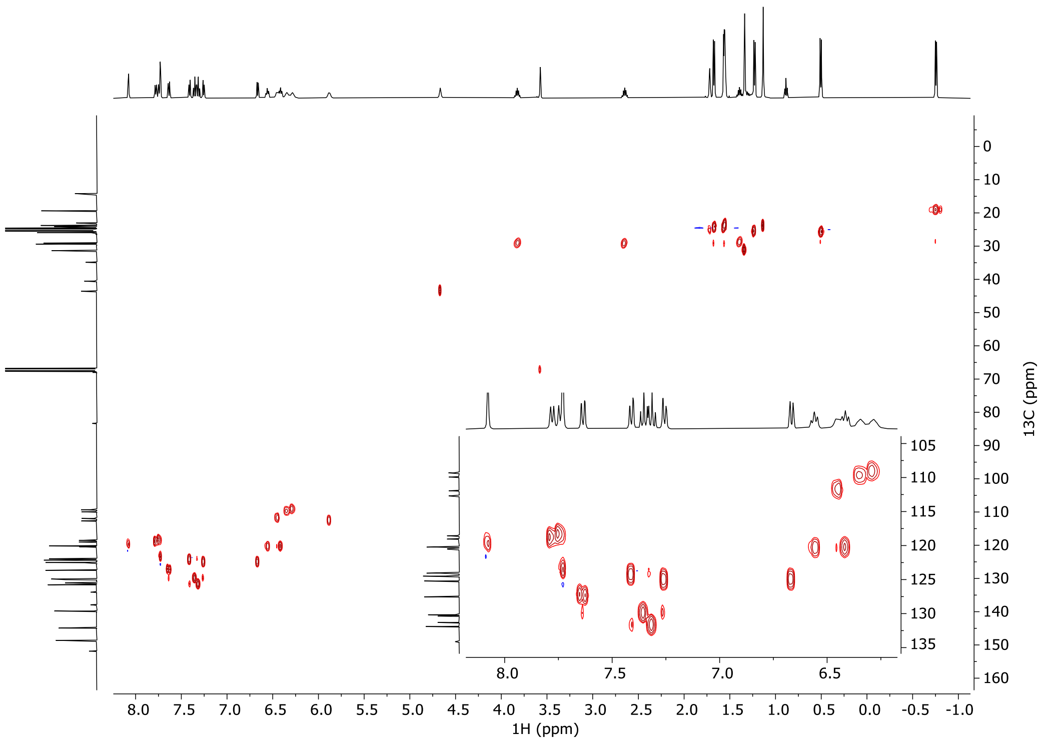

Figure S39. ^{1}H/^{13}C HSQC NMR (d_8-THF) spectrum of **9**.

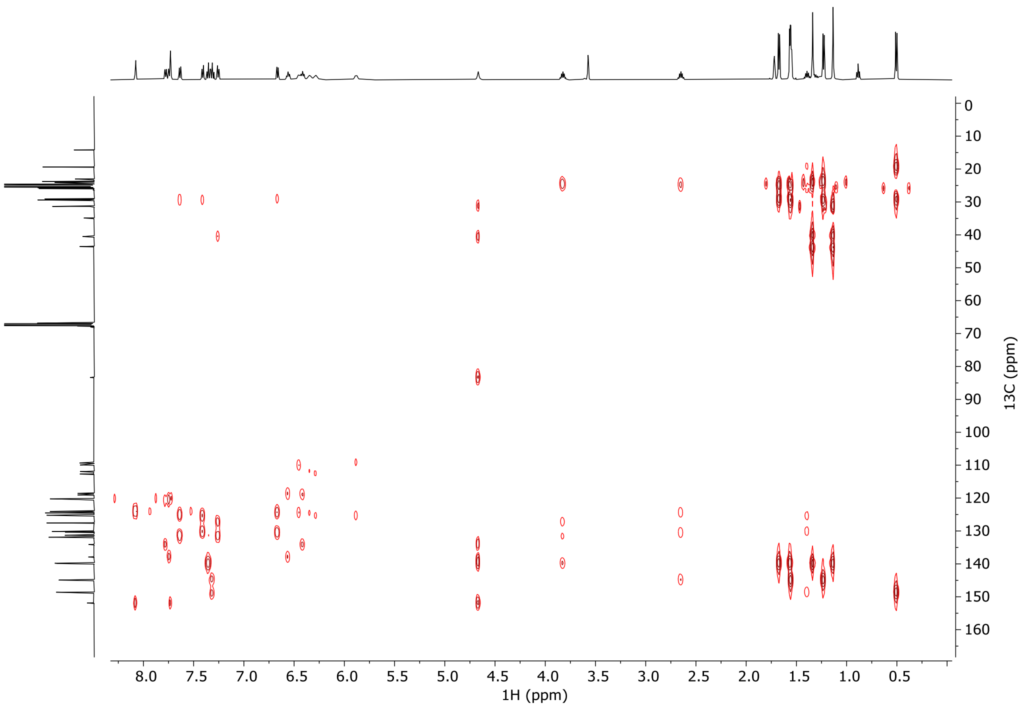

Figure S40. $^{1}H/^{13}C$ HMBC NMR (d_8-THF) spectrum of **9**.

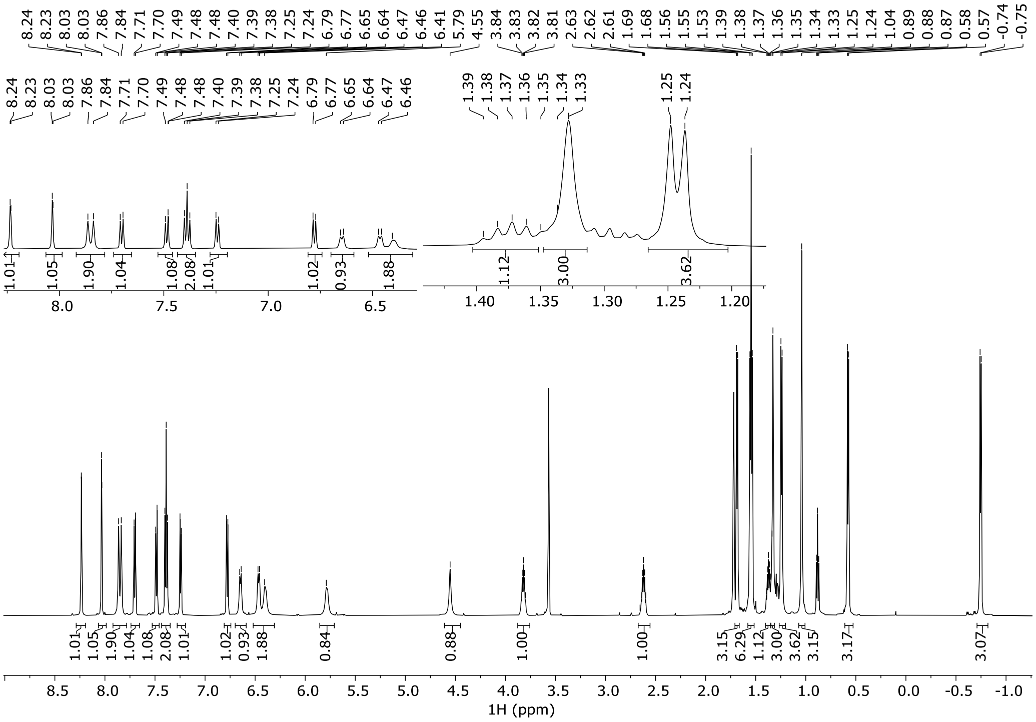

Figure S41. ^{1}H NMR (600 MHz, d_8-THF, 254 K) spectrum of **10**.

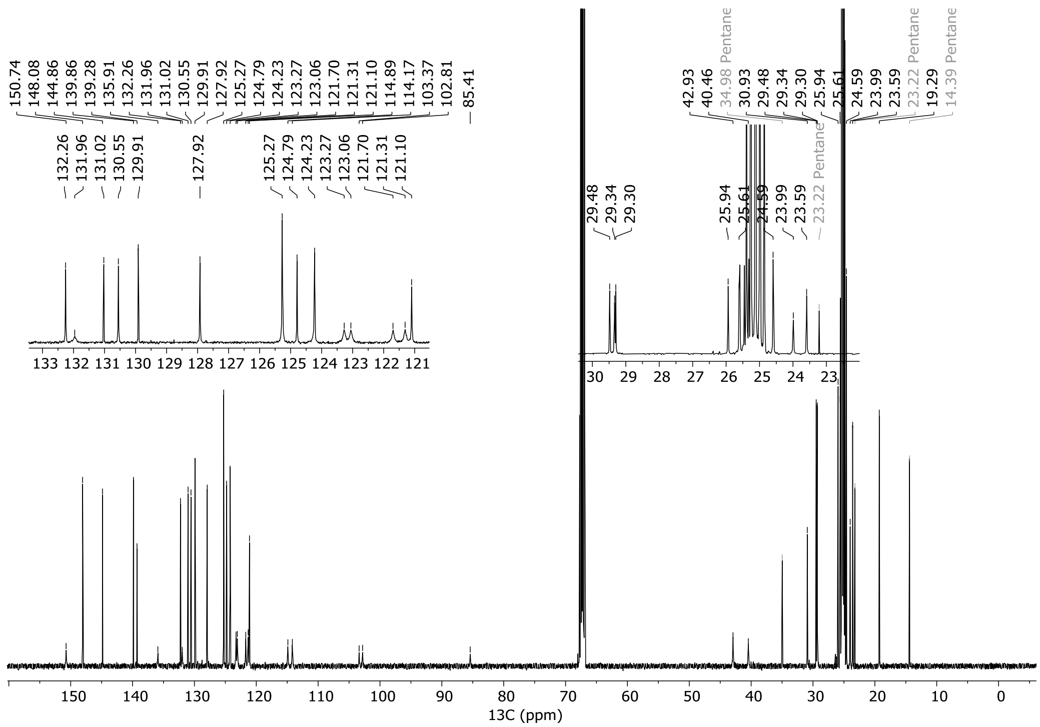

Figure S42. {^{1}H}^{13}C NMR (126 MHz, d_8-THF, 254 K) spectrum of **10**.

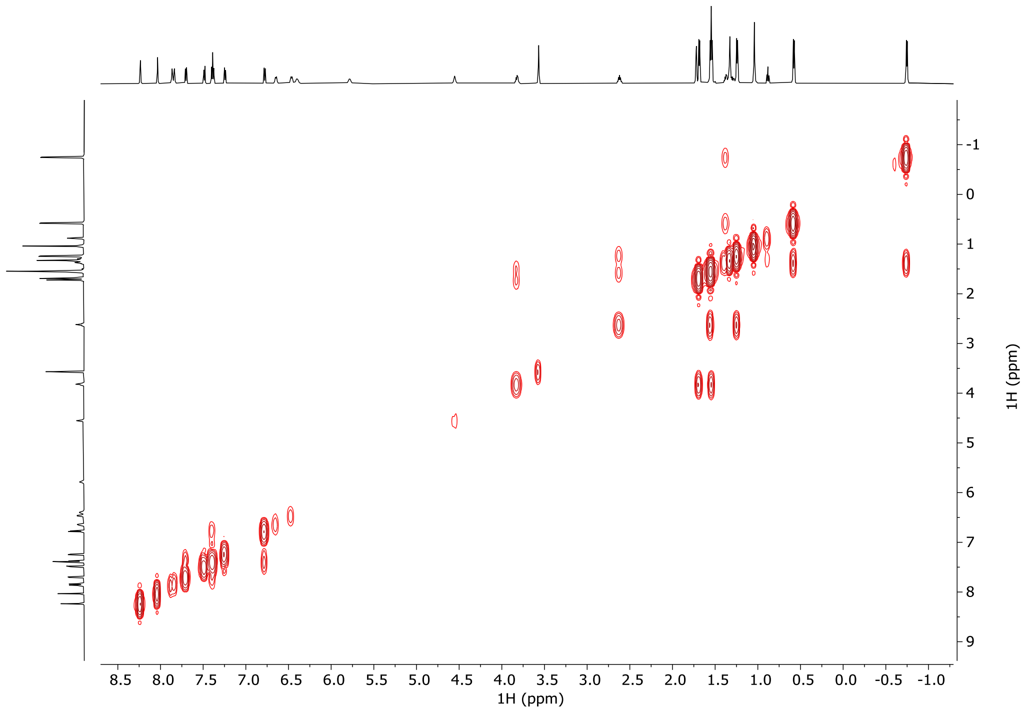

Figure S43. ^{1}H COSY NMR (d_8-THF, 254 K) spectrum of **10**.

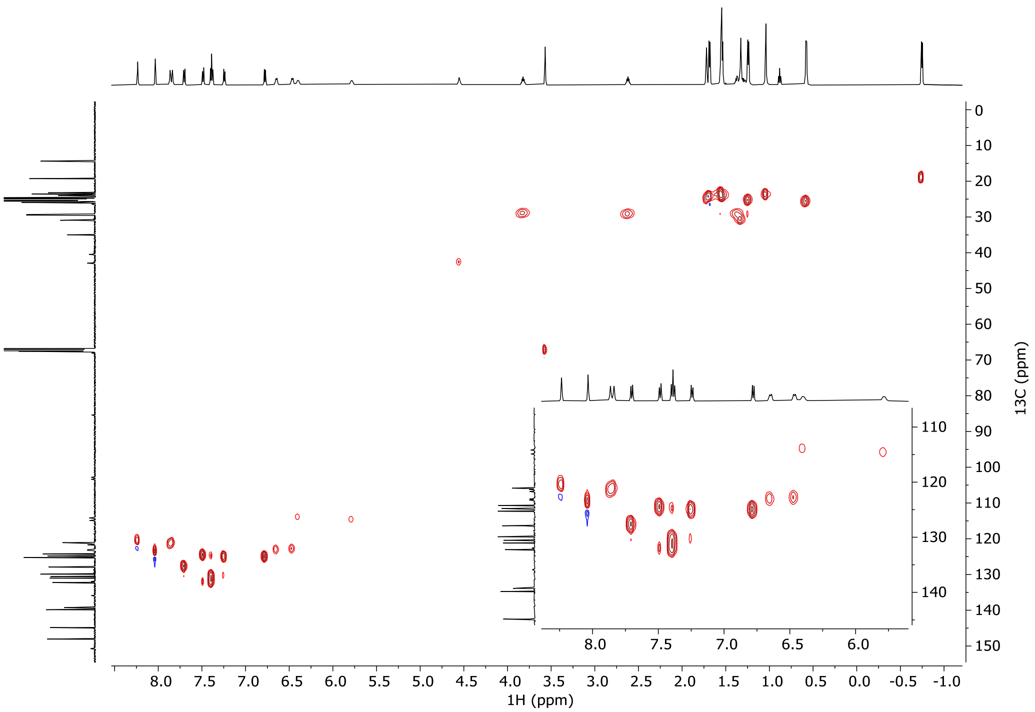

Figure S44. $^{1}H/^{13}C$ HSQC NMR (d_8-THF, 254 K) spectrum of **10**.

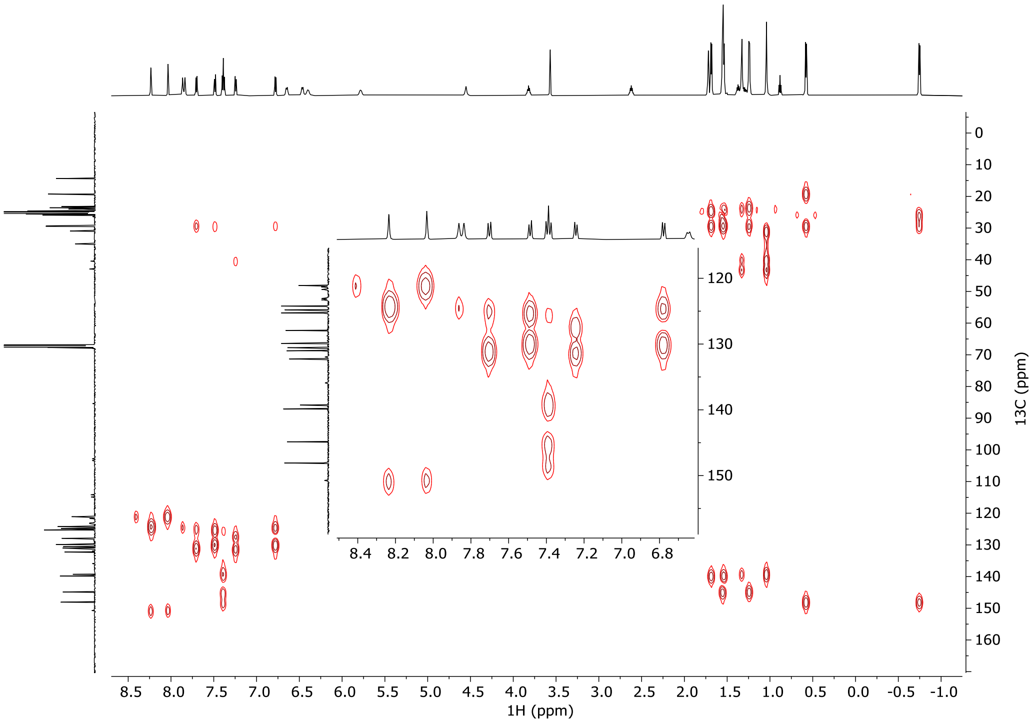

Figure S45. $^{1}H/^{13}C$ HMBC NMR (d_8-THF, 254 K) spectrum of **10**.

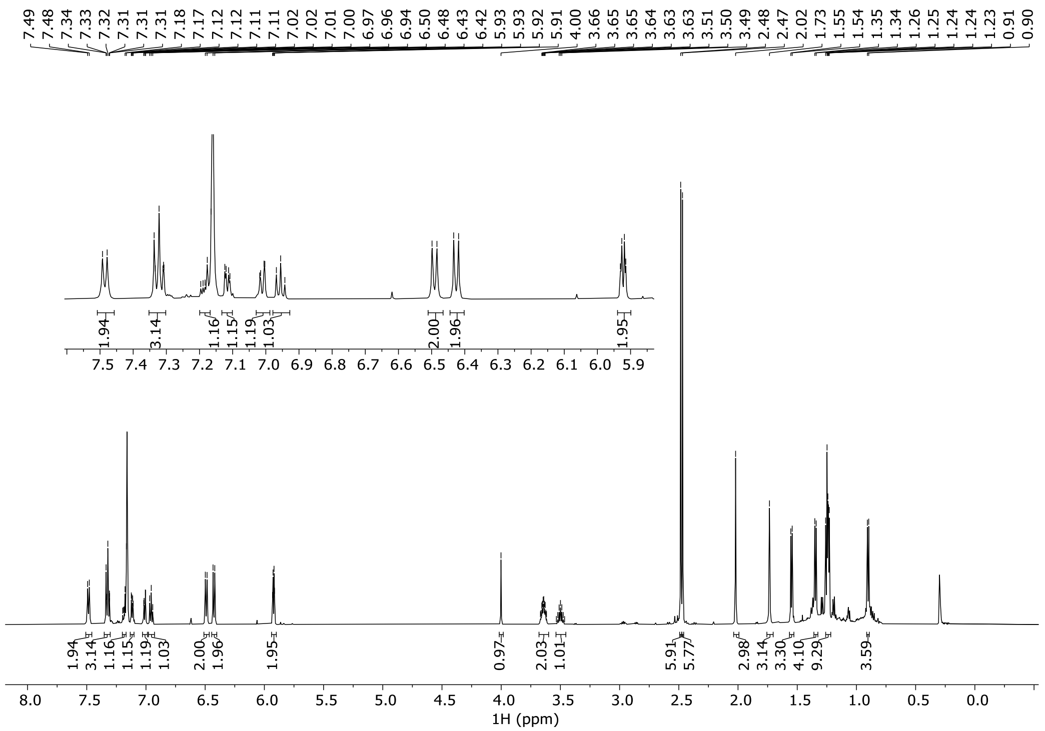

Figure S46. ^{1}H NMR (600 MHz, C_6D_6) spectrum of **11**.

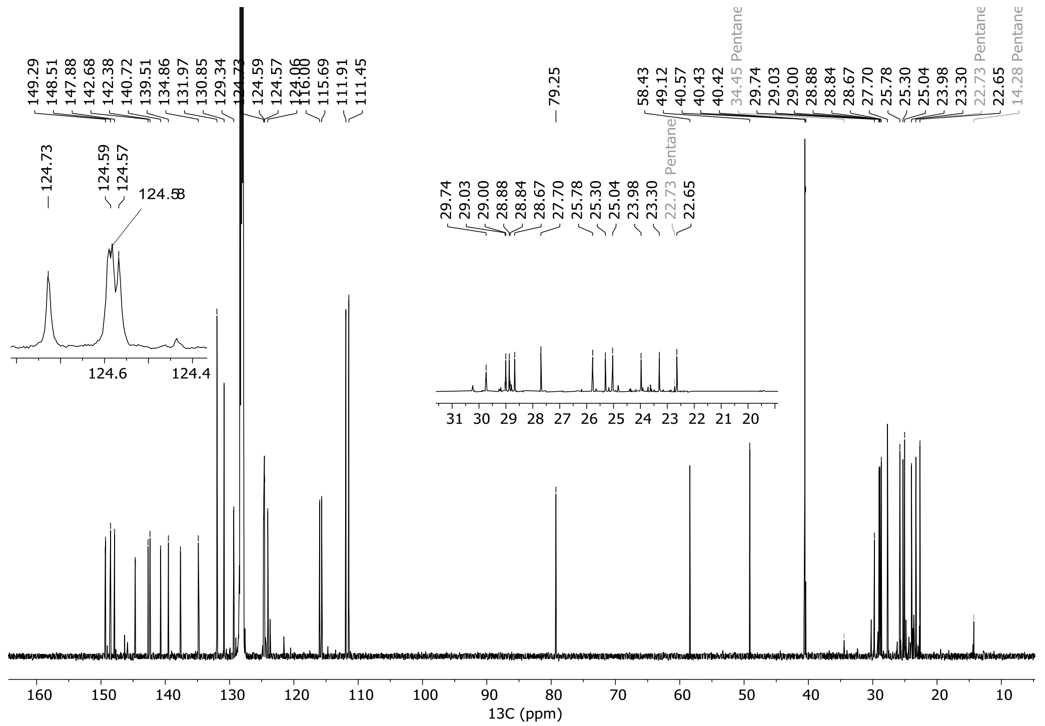

Figure S47. {^{1}H}^{13}C NMR (151 MHz, C_6D_6) spectrum of **11**.

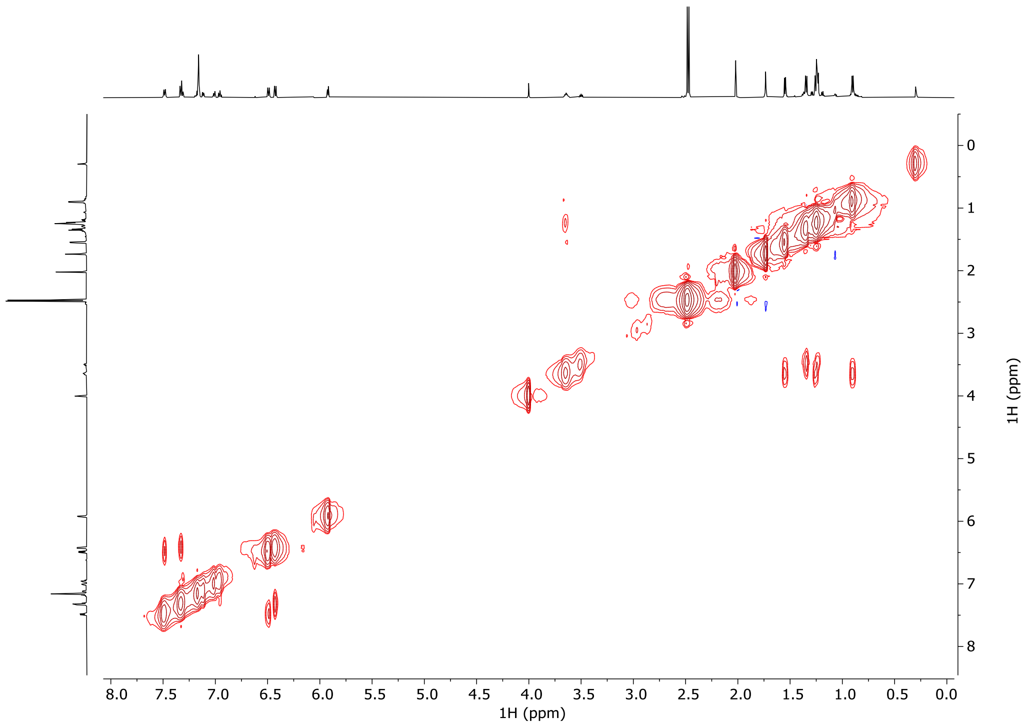

Figure S48. ${}^{1}H$ COSY NMR (C_6D_6) spectrum of **11**.

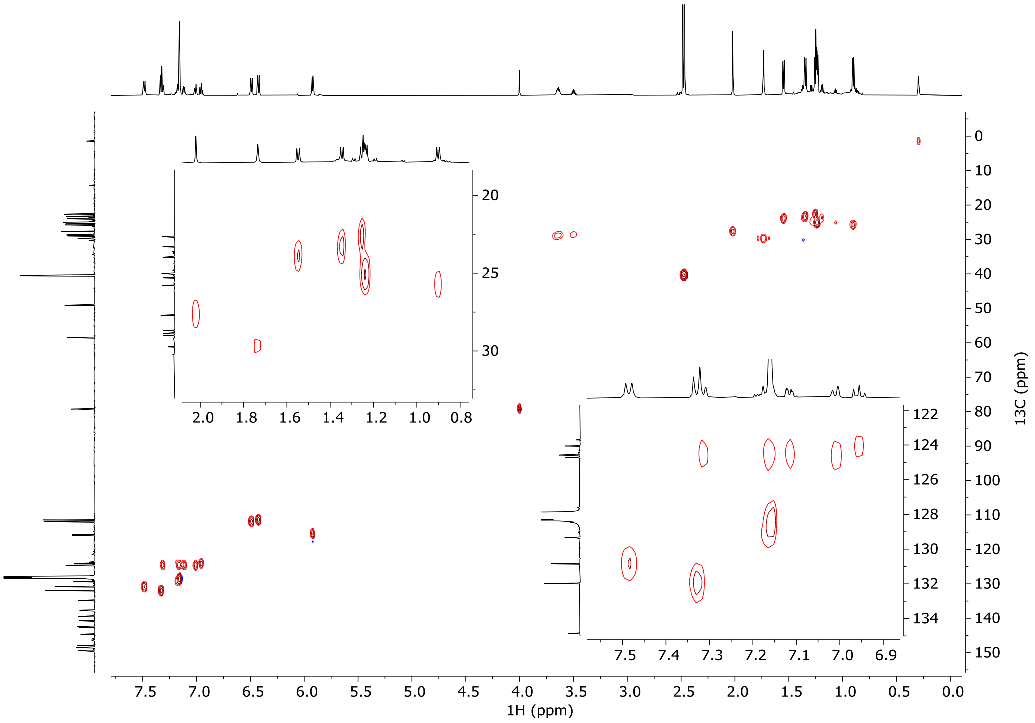

Figure S49. ${}^{1}H/{}^{13}C$ HSQC NMR (C_6D_6) spectrum of **11**.

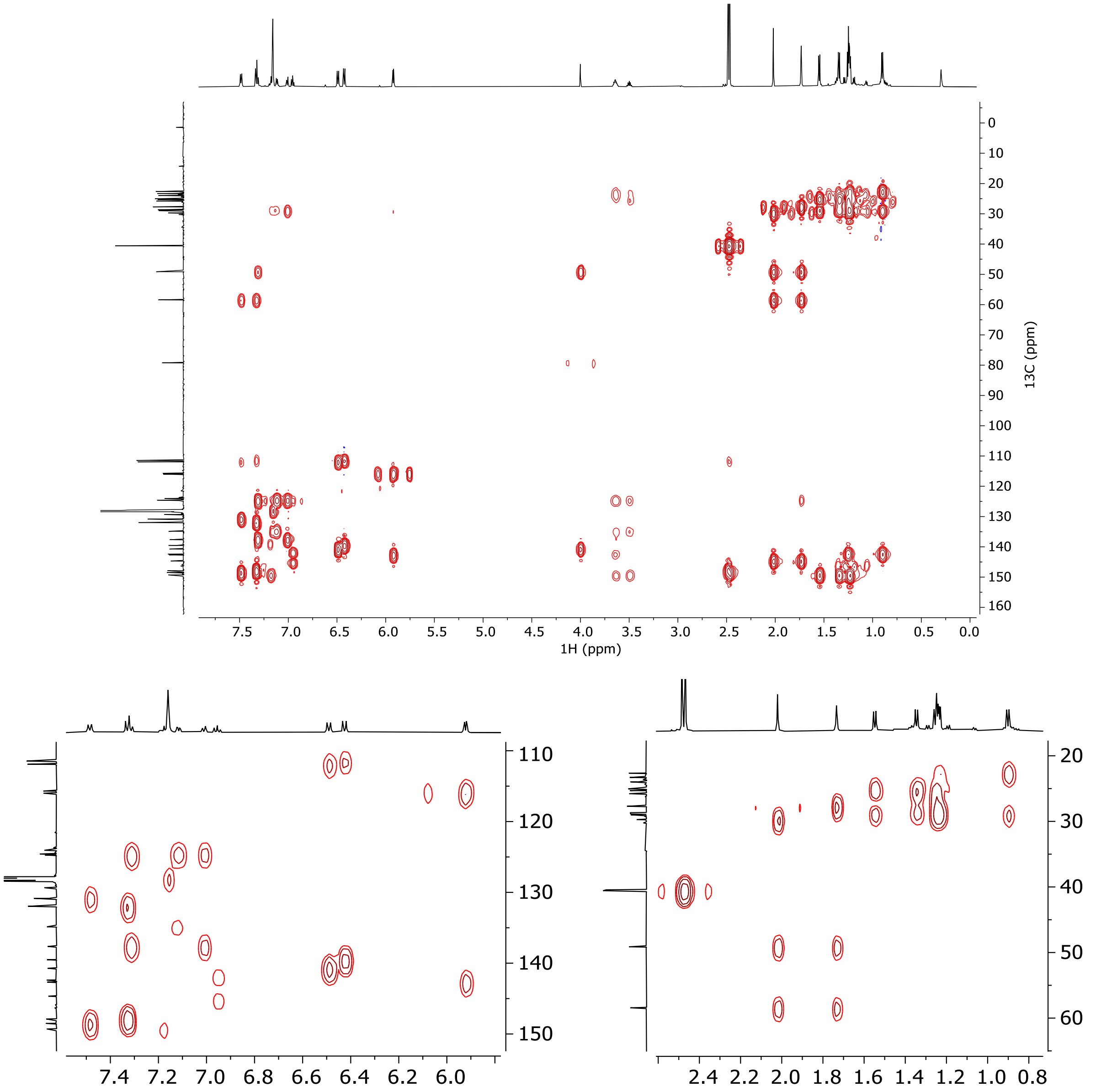

Figure S50. $^{1}H/^{13}C$ HMBC NMR (C_6D_6) spectrum of **11** (top), and two zoomed-in regions (below).

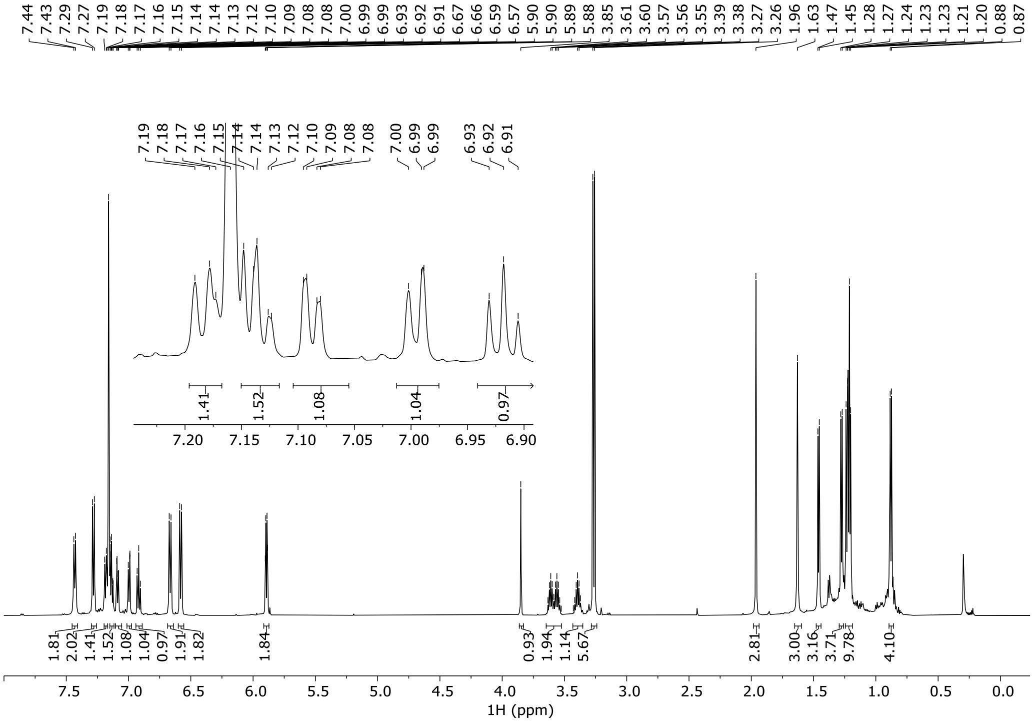

Figure S51. ^{1}H NMR (600 MHz, C_6D_6) spectrum of **12**.

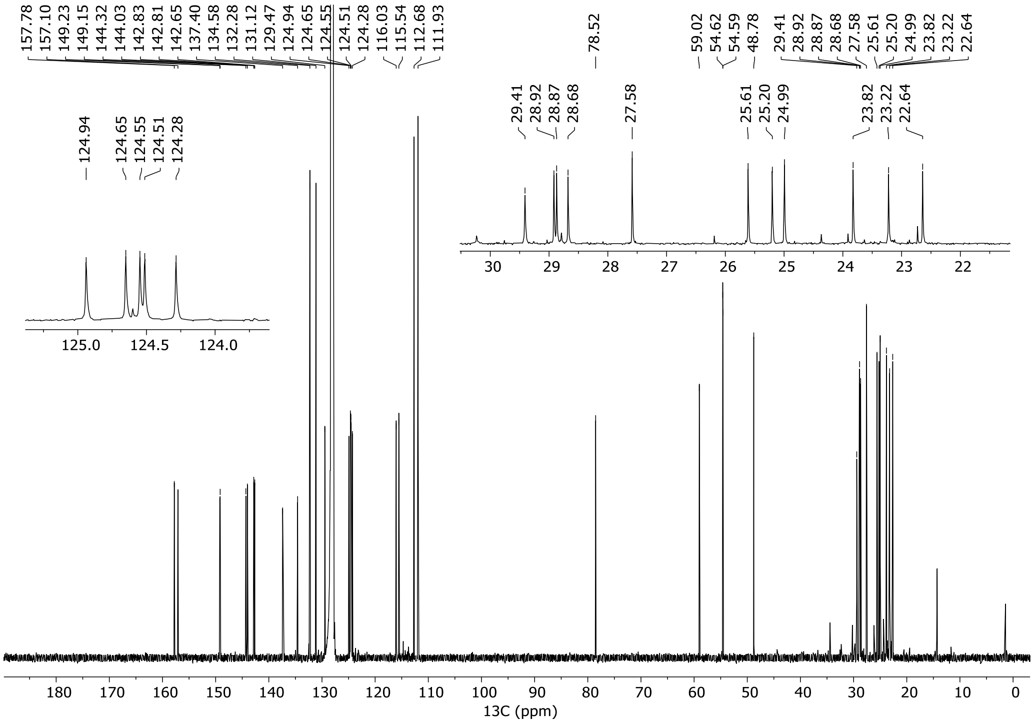

Figure S52. {^{1}H}^{13}C NMR (151 MHz, C_6D_6) spectrum of **12**.

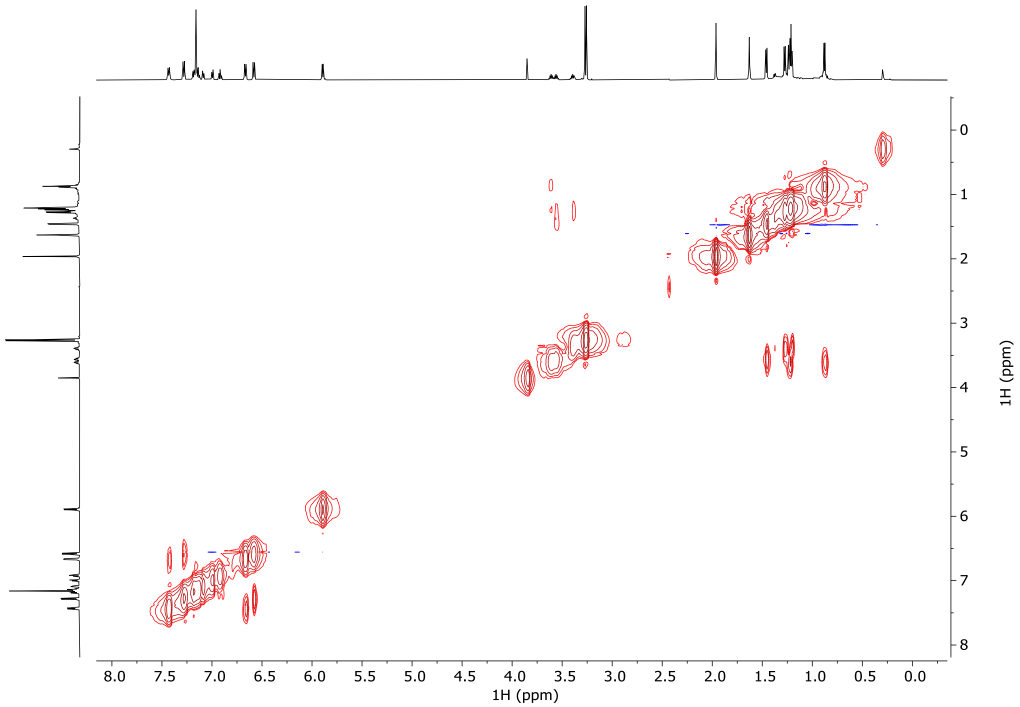

Figure S53. ^{1}H COSY NMR (C_6D_6) spectrum of **12**.

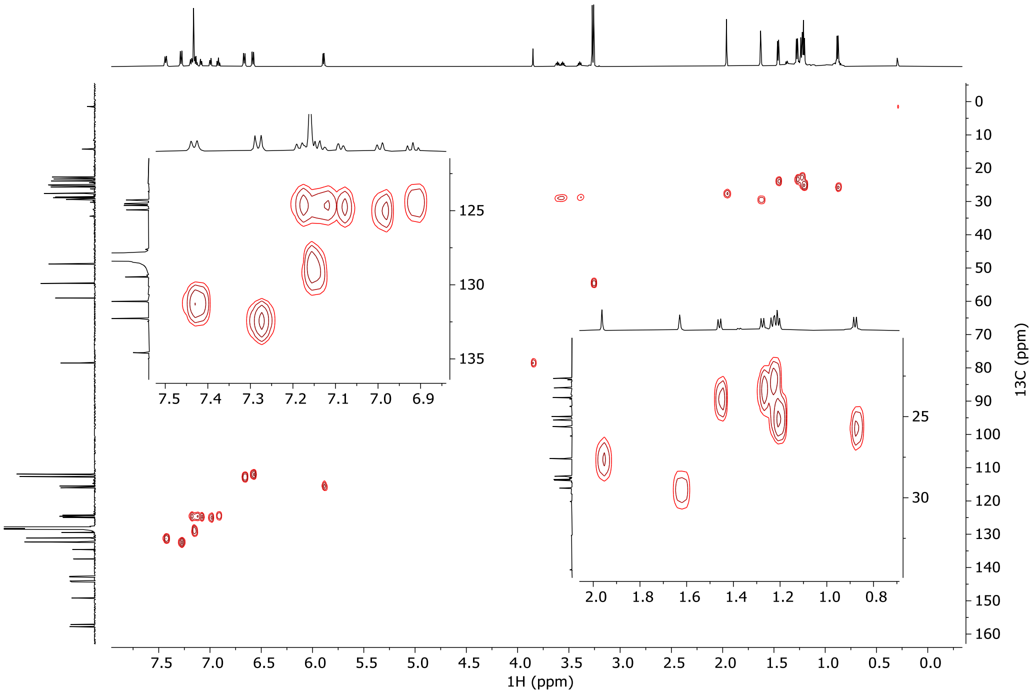

Figure S54. ^{1}H/^{13}C HSQC NMR (C_6D_6) spectrum of **12**.

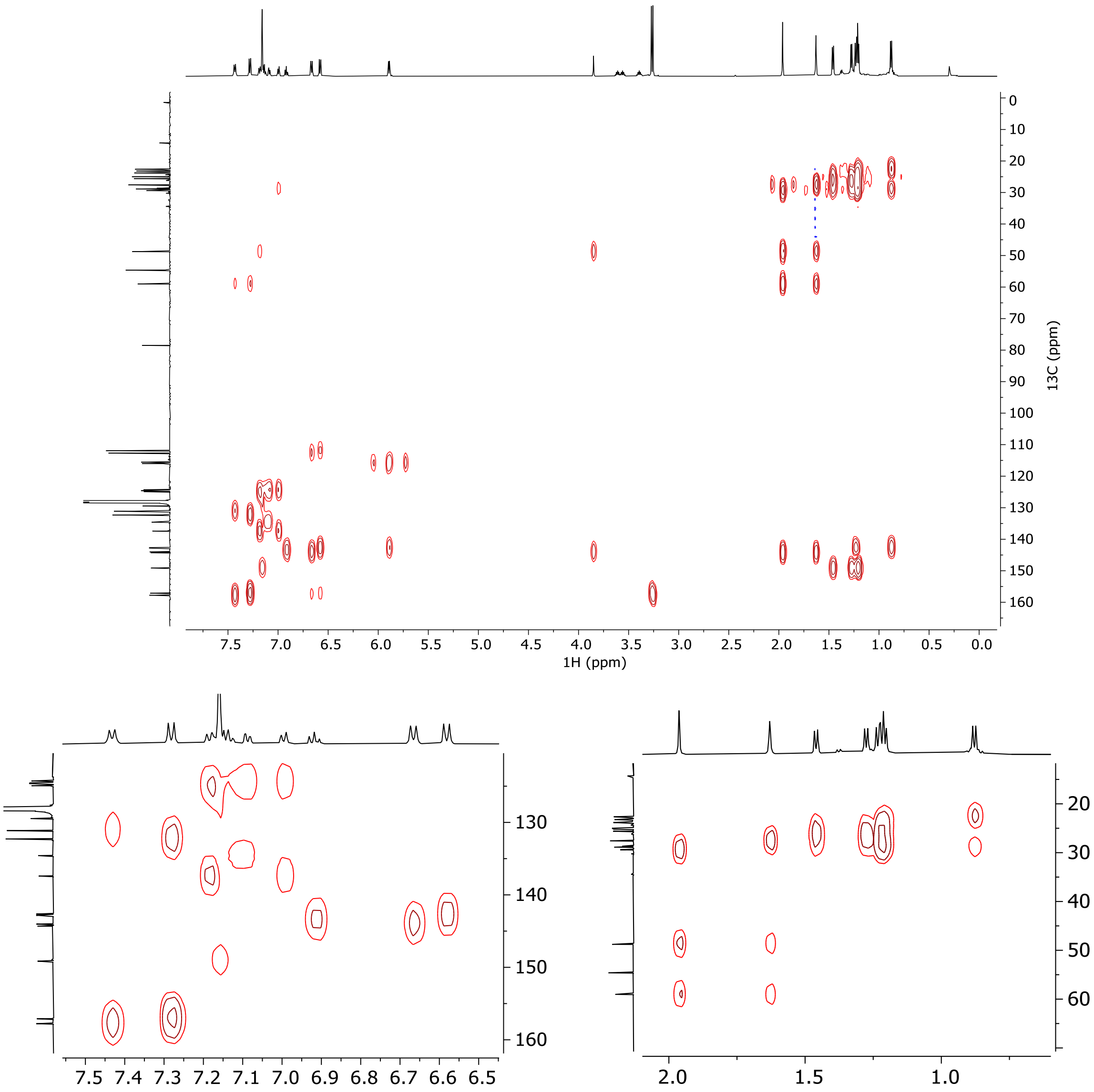

Figure S55. $^1H/^{13}C$ HMBC NMR (C_6D_6) spectrum of **12** (top), and two zoomed-in regions (below).

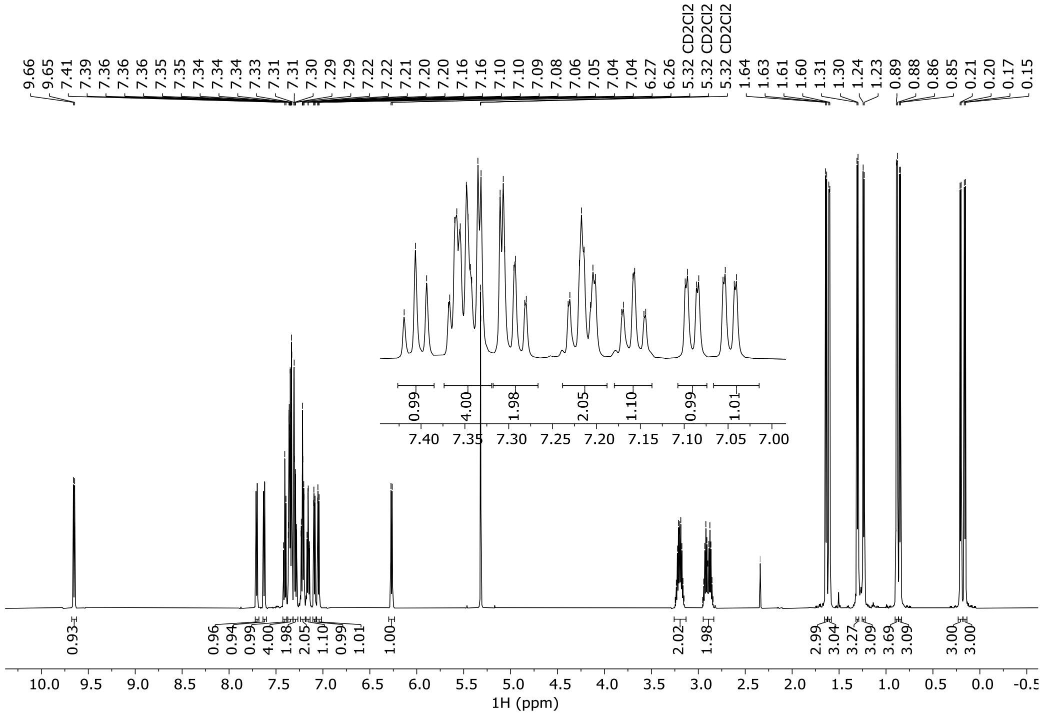

Figure S56. ^{1}H NMR (600 MHz, CD_2Cl_2) spectrum of **13**.

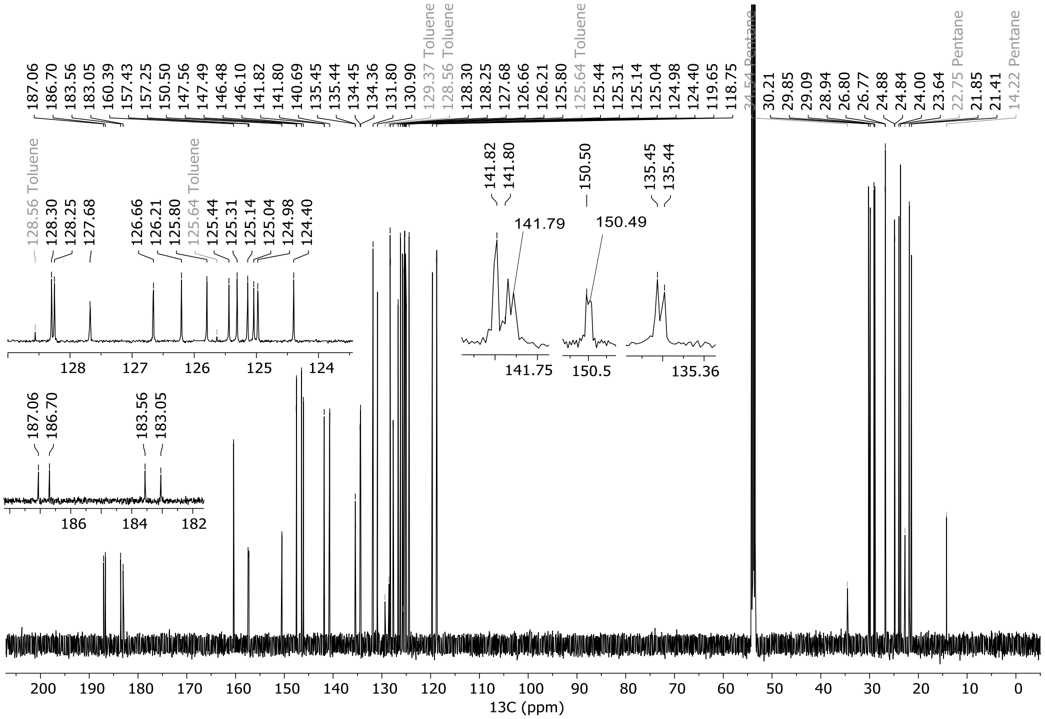

Figure S57. $\{^{1}H\}^{13}C$ NMR (151 MHz, CD_2Cl_2) spectrum of **13**.

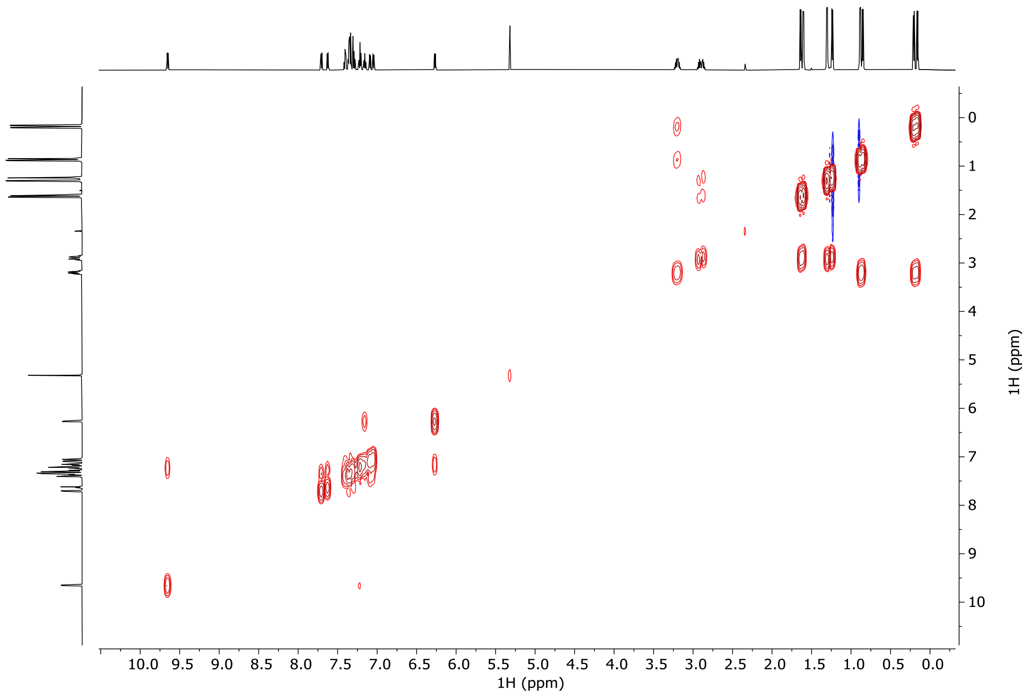

Figure S58. ^{1}H COSY NMR (CD_2Cl_2) spectrum of **13**.

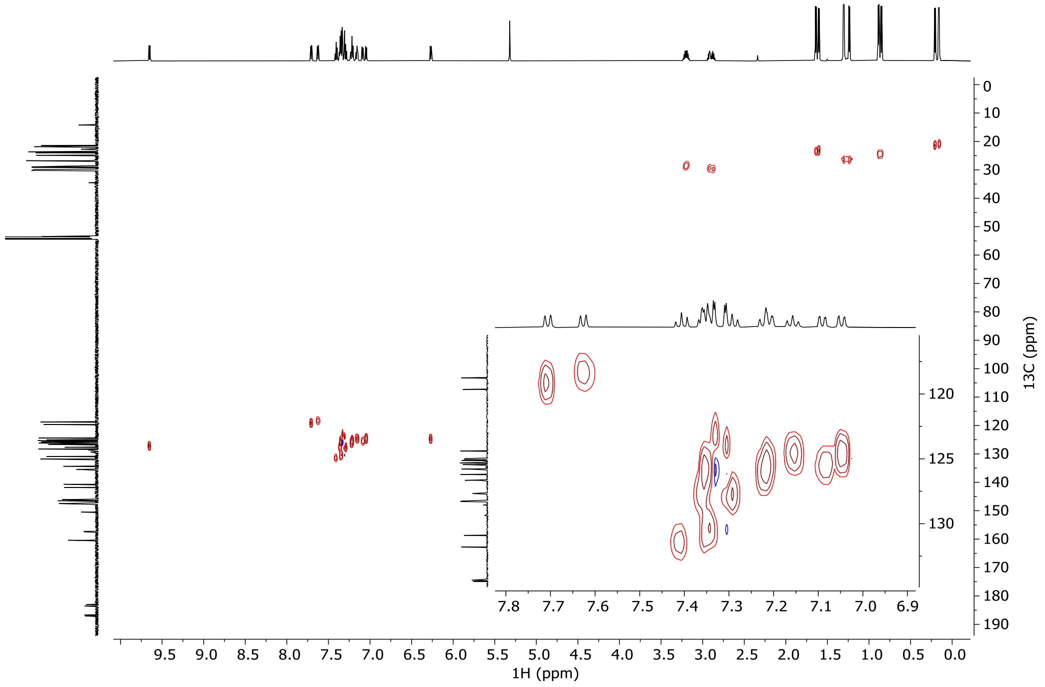

Figure S59. ^{1}H/^{13}C HSQC NMR (CD_2Cl_2) spectrum of **13**.

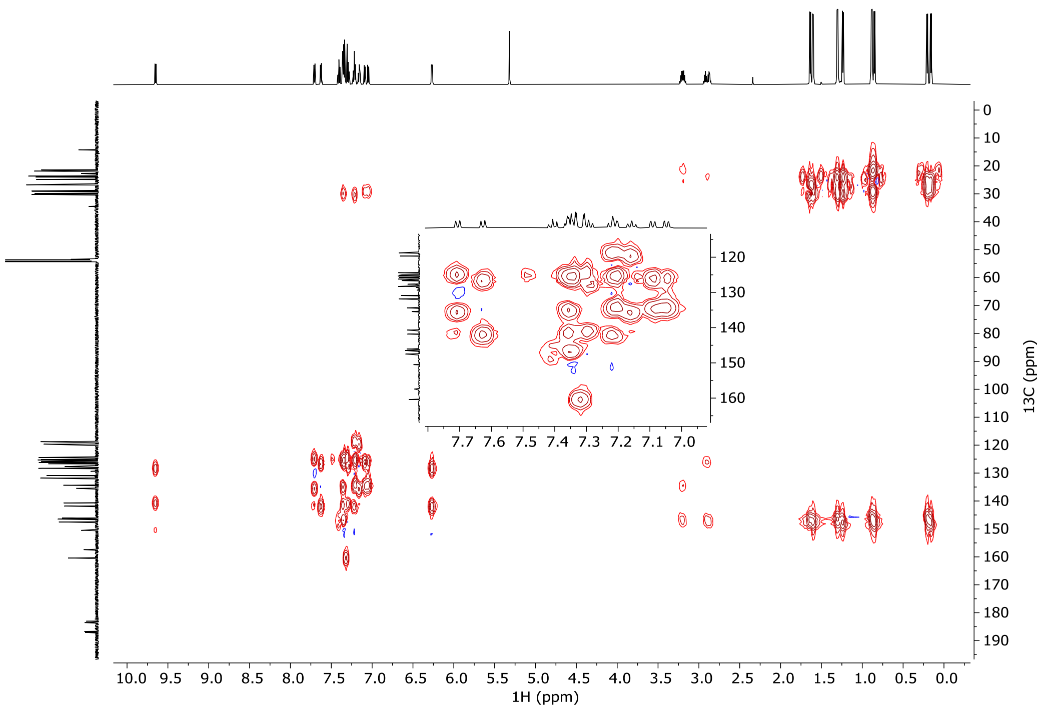

Figure S60. $^{1}H/^{13}C$ HMBC NMR (CD_2Cl_2) spectrum of **13**.

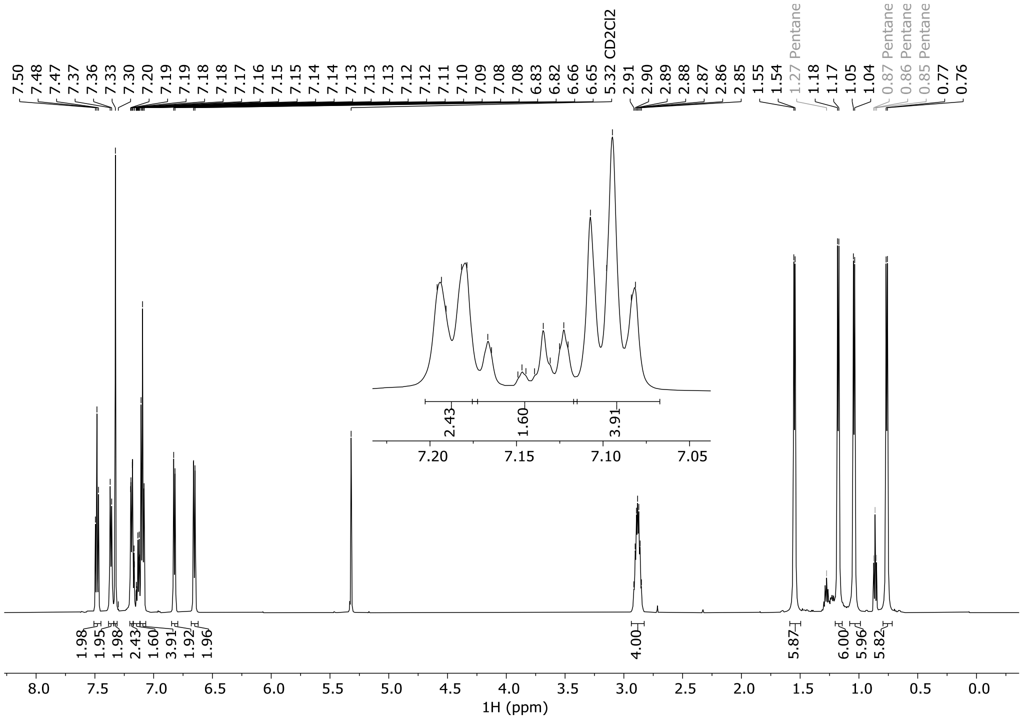

Figure S61. ^{1}H NMR (600 MHz, CD_2Cl_2, 270 K) spectrum of **14**.

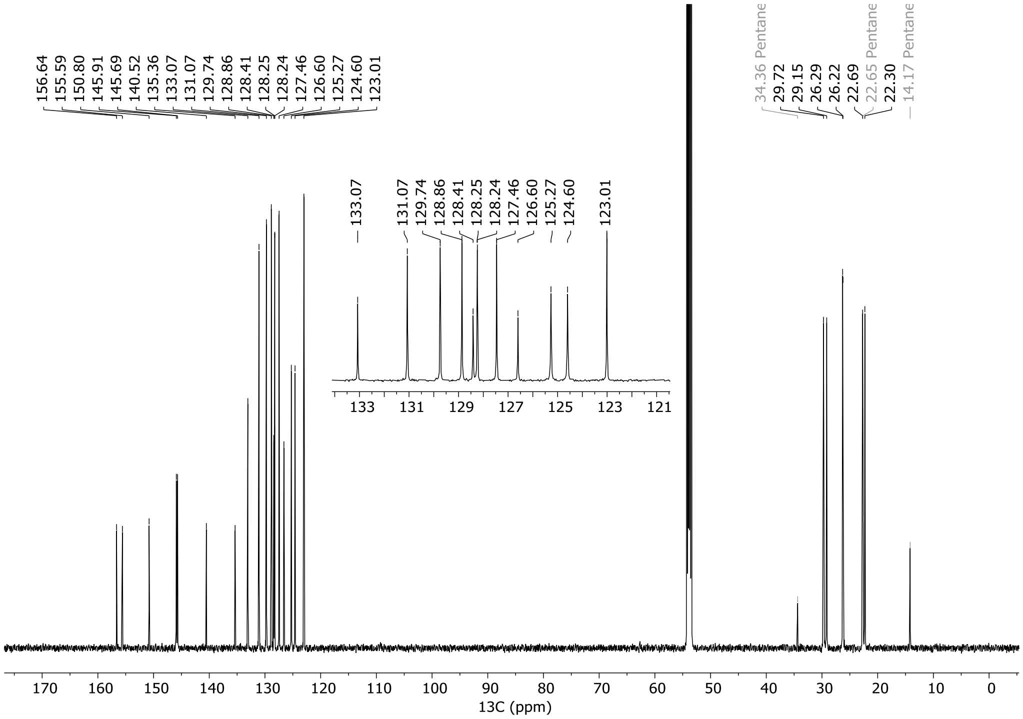

Figure S62. {^{1}H}^{13}C NMR (151 MHz, CD_2Cl_2, 270 K) spectrum of **14**.

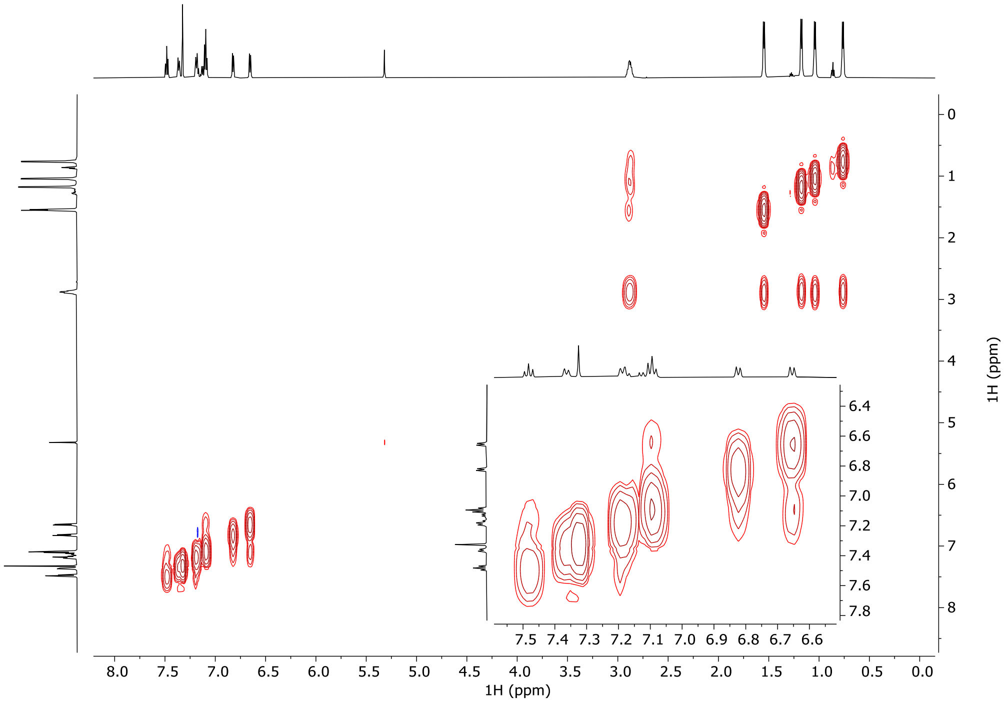

Figure S63. ^{1}H COSY NMR (CD_2Cl_2, 270 K) spectrum of **14**.

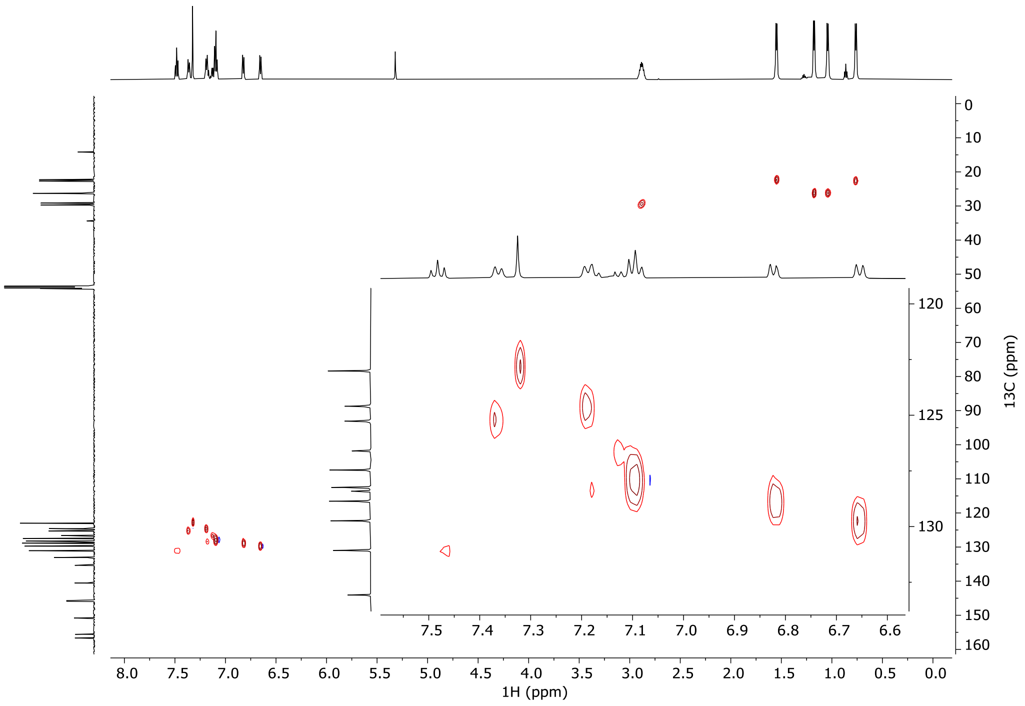

Figure S64. $^{1}H/^{13}C$ HSQC NMR (CD_2Cl_2, 270 K) spectrum of **14**.

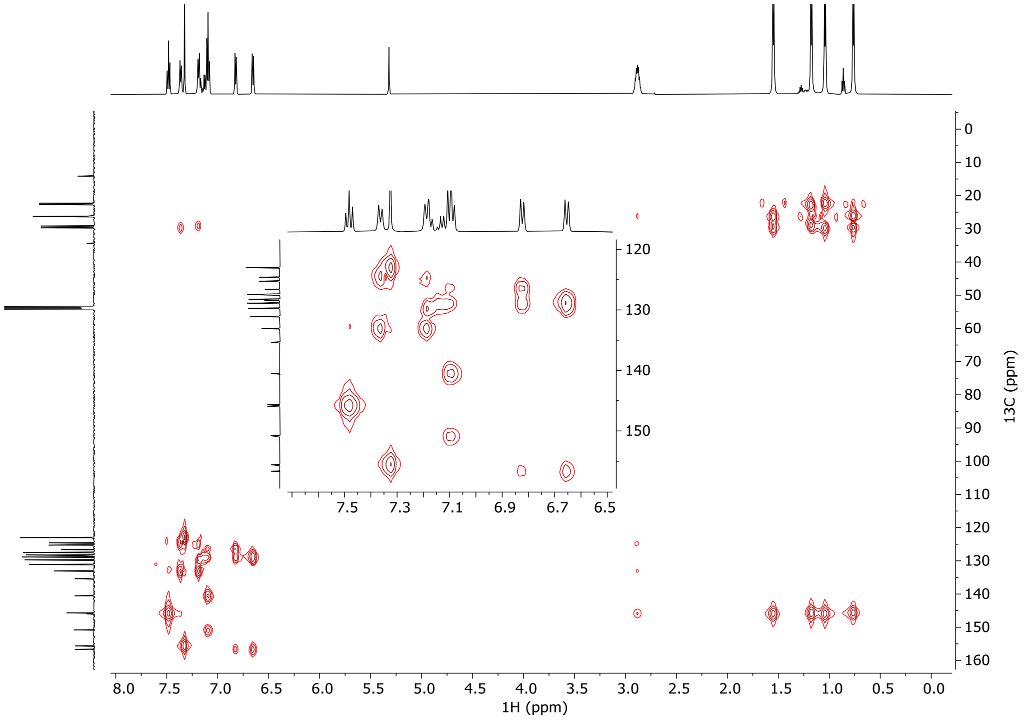

Figure S65. $^{1}H/^{13}C$ HMBC NMR (CD_2Cl_2, 270 K) spectrum of **14**.

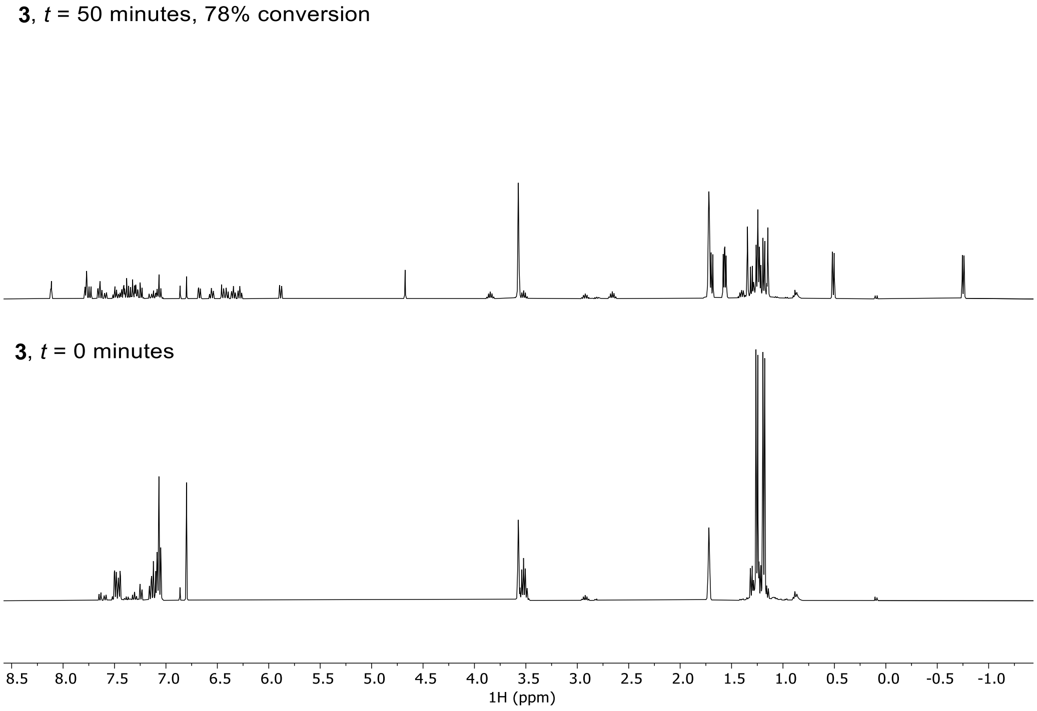

Figure S66. Stacked ^{1}H NMR (400 MHz, d_8-THF) spectra of **3** (~17 μmol/mL) at *t* = 0 (bottom) and after heating at 60 °C for 50 min (top).

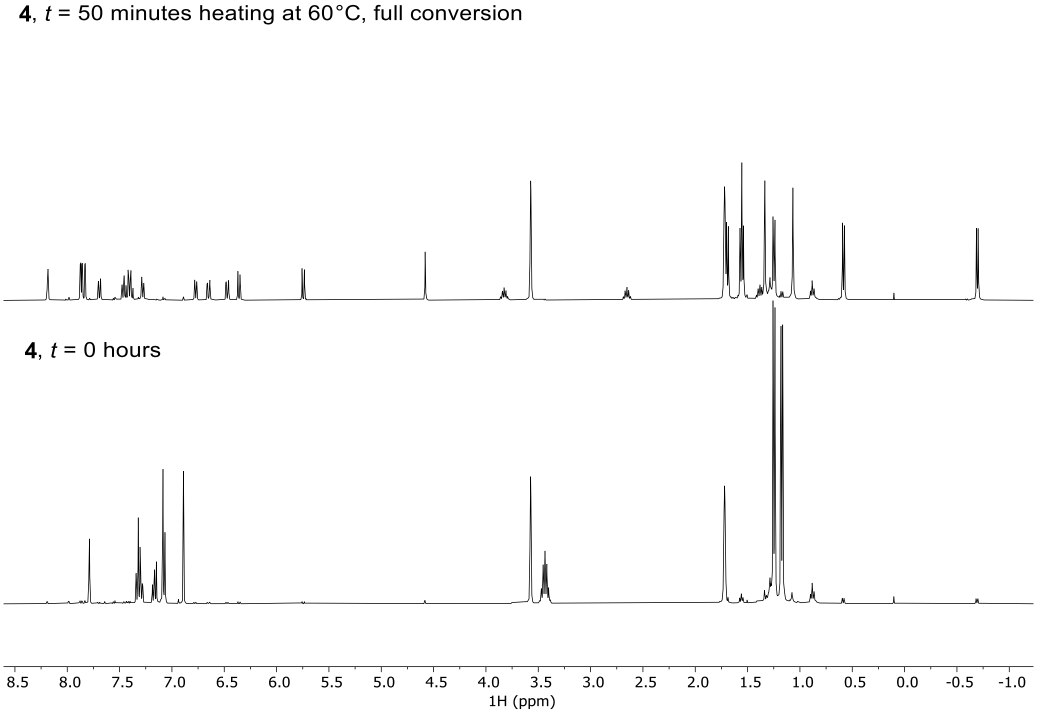

Figure S67. Stacked ^{1}H NMR (400 MHz, d_8-THF) spectra of **4** (~17 μmol/mL) at *t* = 0 (bottom) and after heating at 60 °C for 50 min (top).

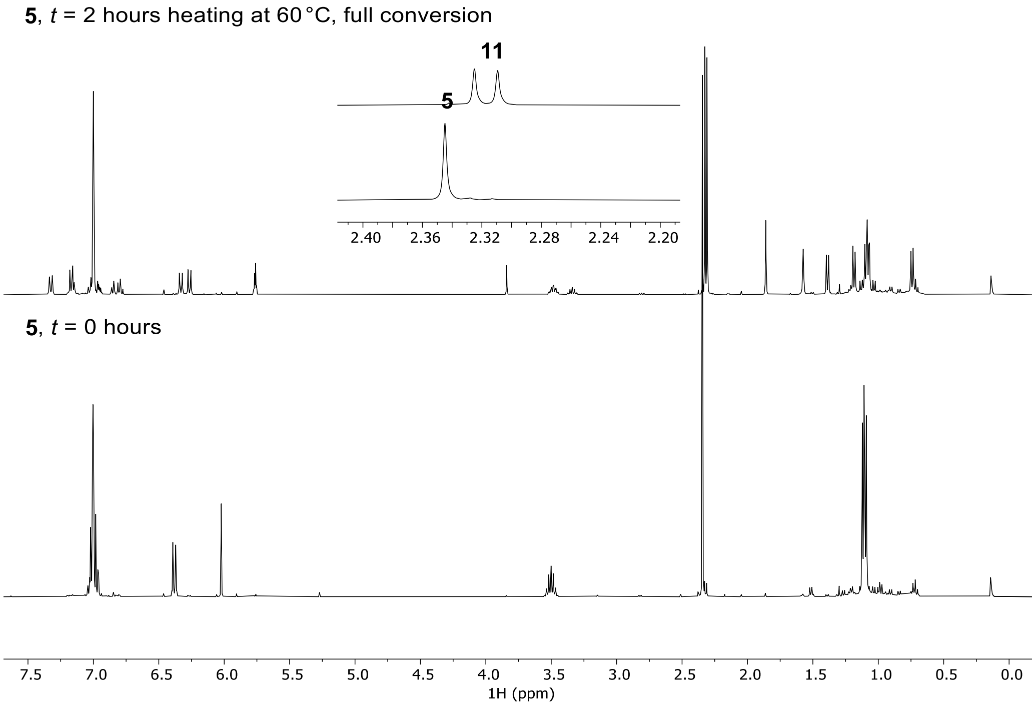

Figure S68. Stacked ^{1}H NMR (400 MHz, C_6D_6) spectra of **5** (~22 μmol/mL) at *t* = 0 (bottom) and after heating at 60 °C for 2 h (top).

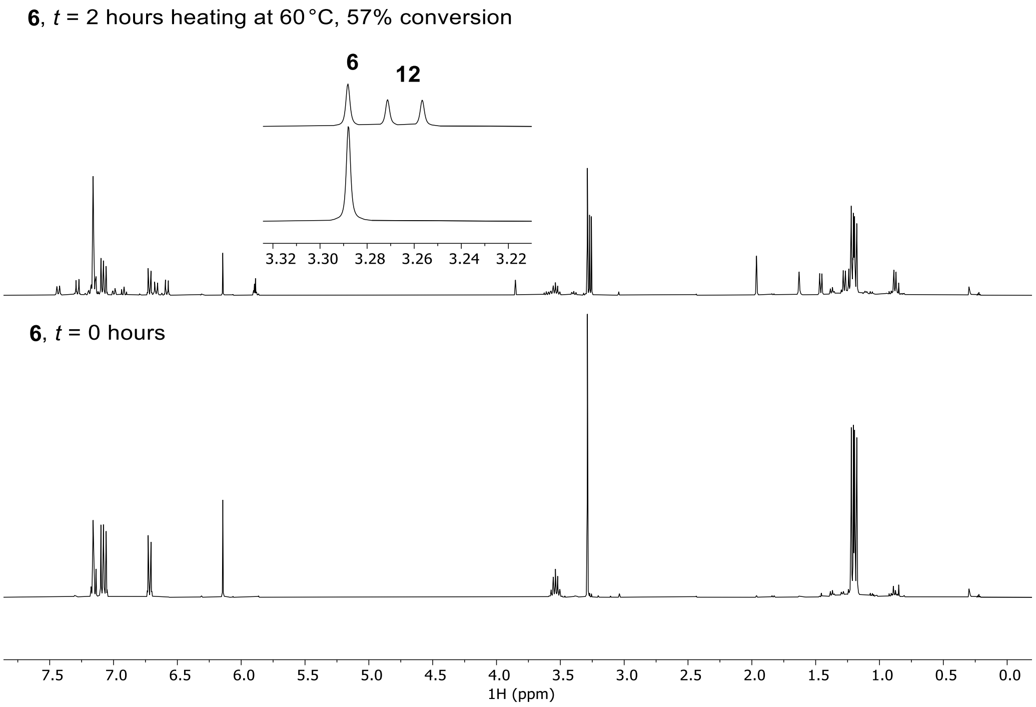

Figure S69. Stacked ^{1}H NMR (400 MHz, C_6D_6) spectra of **6** (~22 μmol/mL) at *t* = 0 (bottom) and after heating at 60 °C for 2 h (top).

4. IR Data

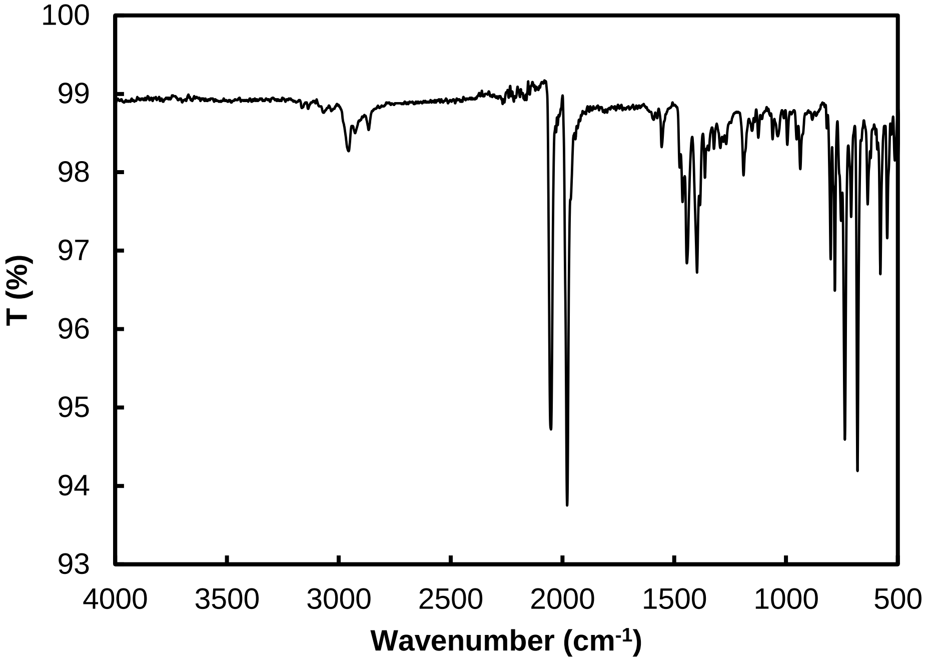

Figure S70. Solid-state IR spectrum of complex **13**.

5. UV/Vis Data

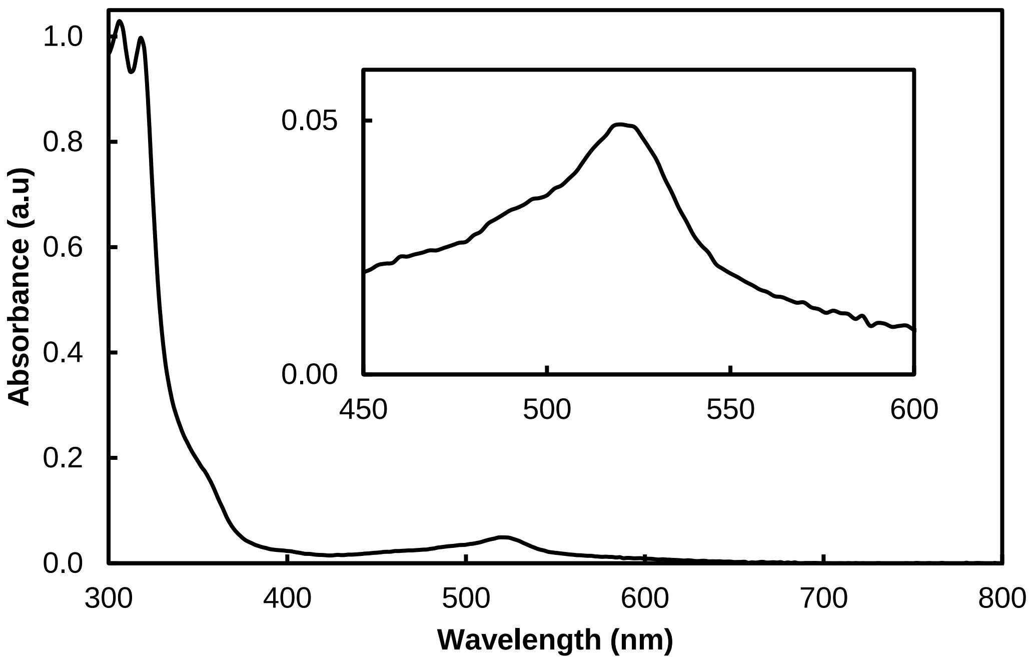

Figure S71. UV/Vis spectrum of **3** (5.9 x 10^{-5} M) in THF.

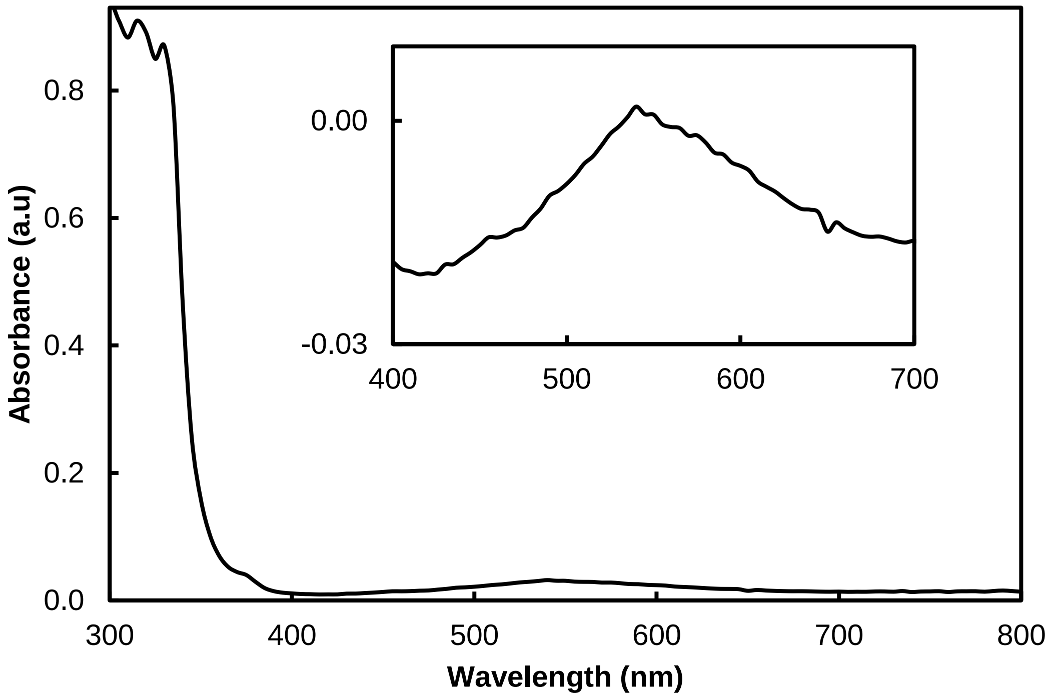

Figure S72. UV/Vis spectrum of **4** (2.8 x 10^{-5} M) in THF.

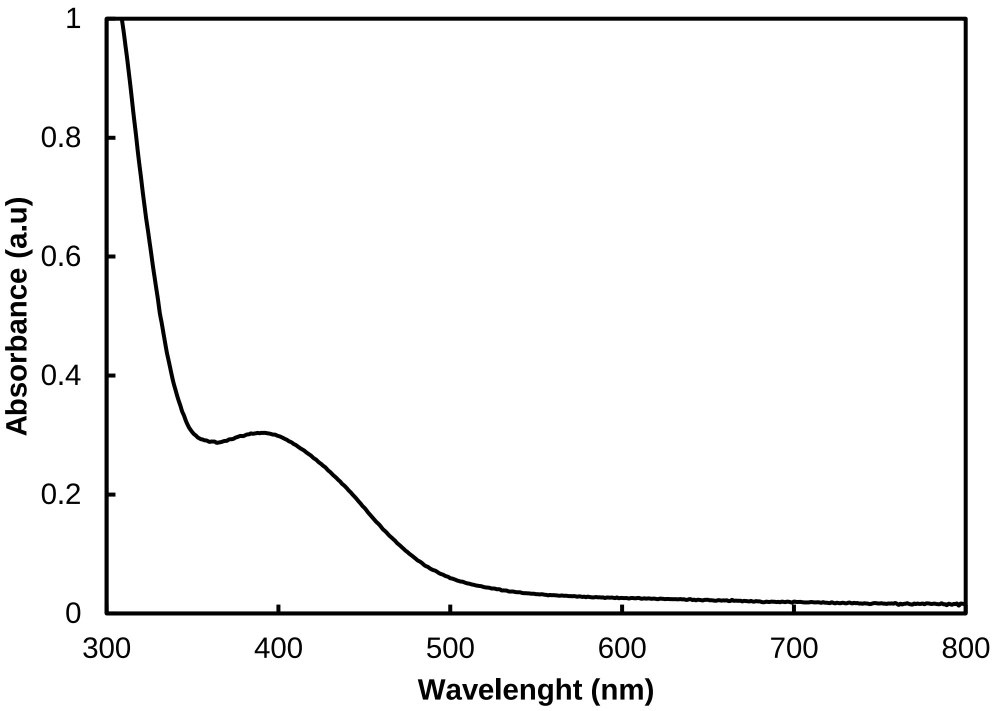

Figure S73. UV/Vis spectrum of **5** (6.4 x 10^{-5} M) in THF.

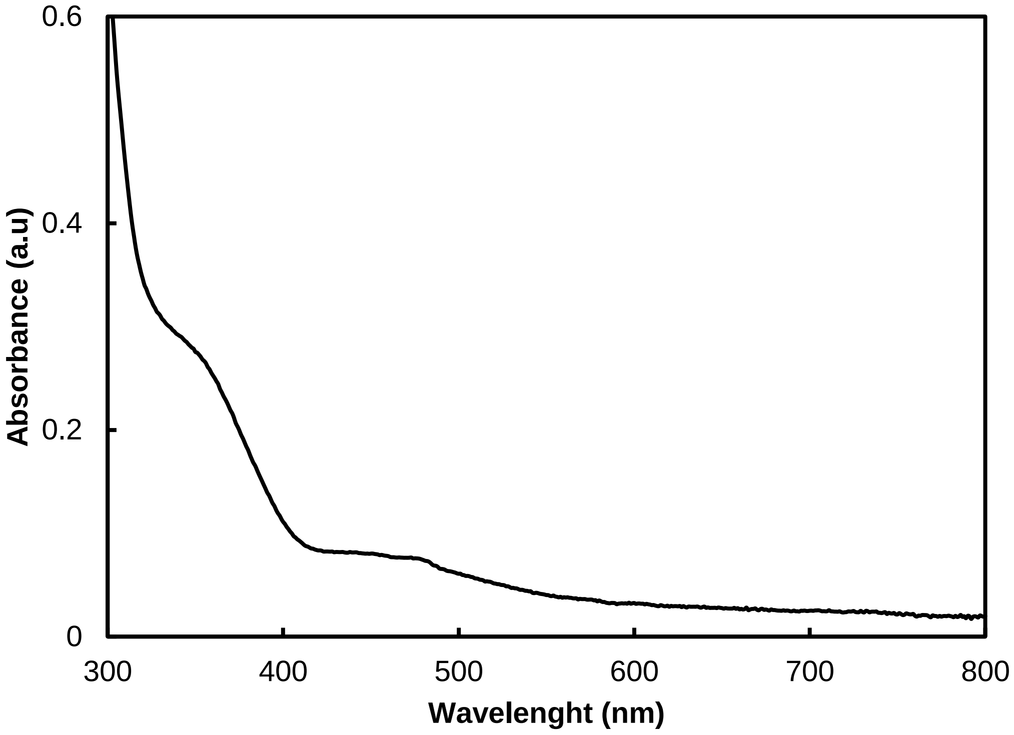

Figure S74. UV/Vis spectrum of **6** (3.8 x 10^{-5} M) in THF.

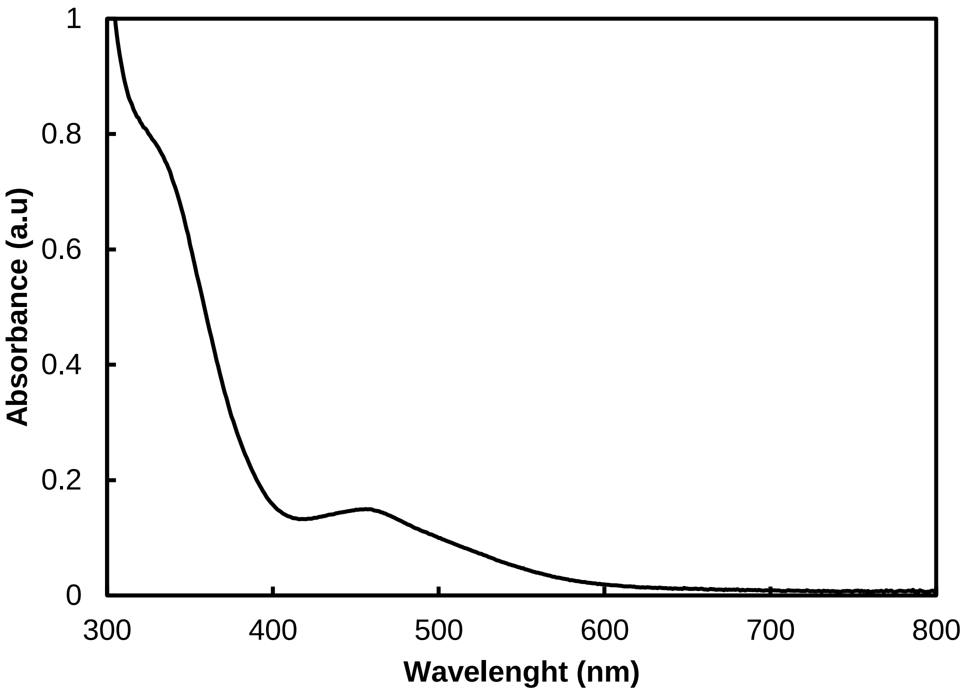

Figure S75. UV/Vis spectrum of **7** (1.6 x 10^{-4} M) in THF.

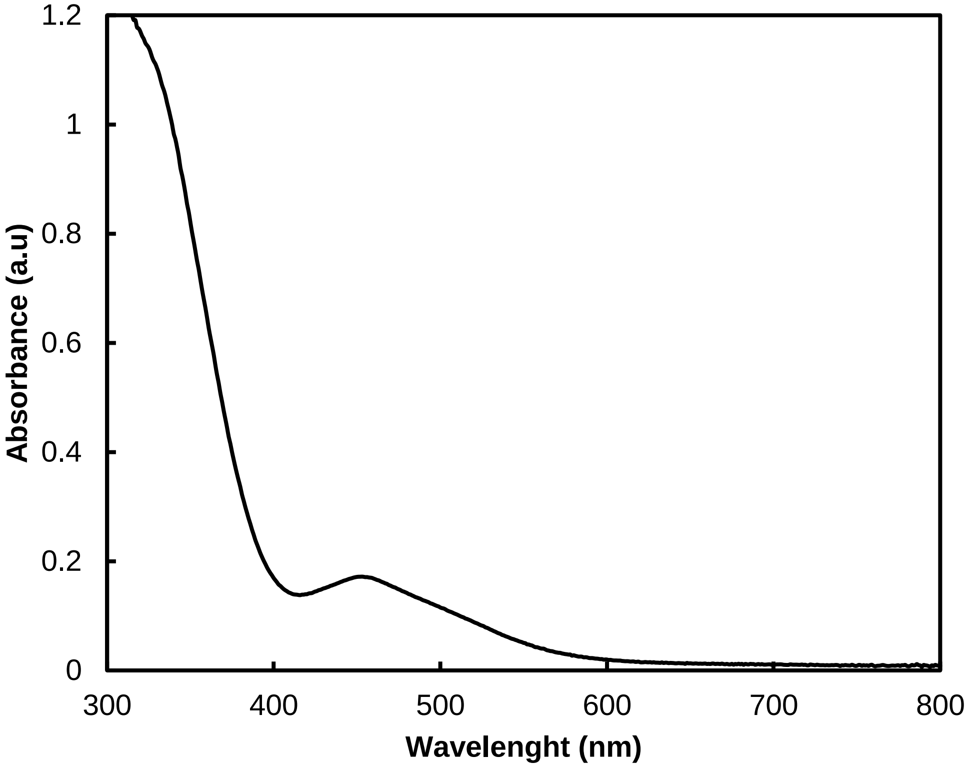

Figure S76. UV/Vis spectrum of **8** (1.9 x 10^{-4} M) in THF.

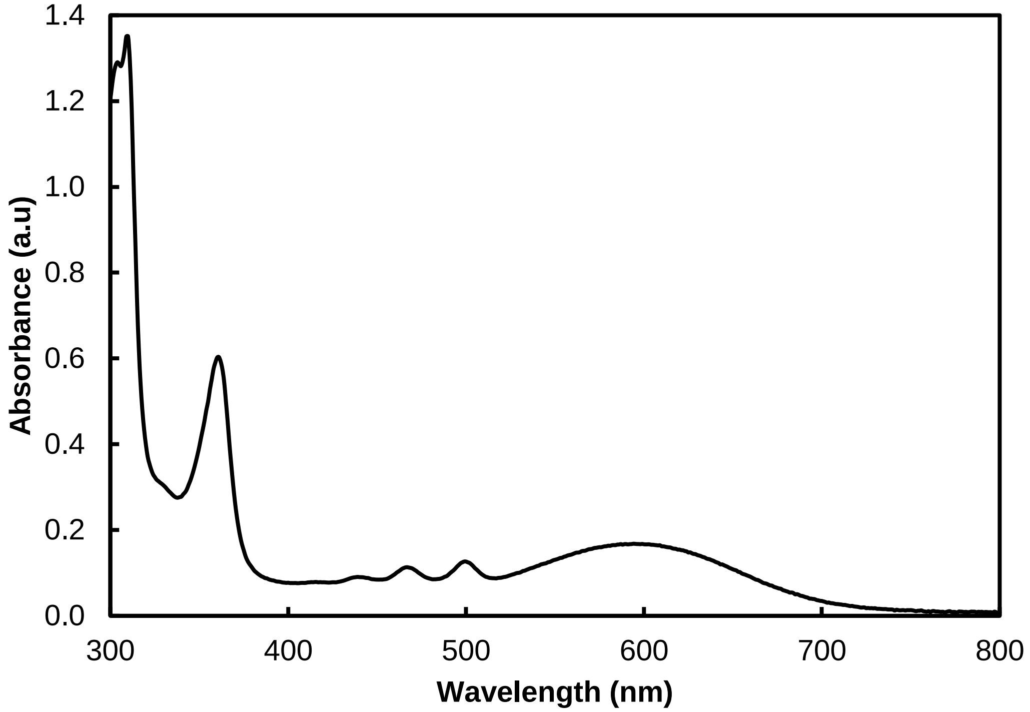

Figure S77. UV/Vis spectrum of **9** (12 x 10^{-5} M) in THF.

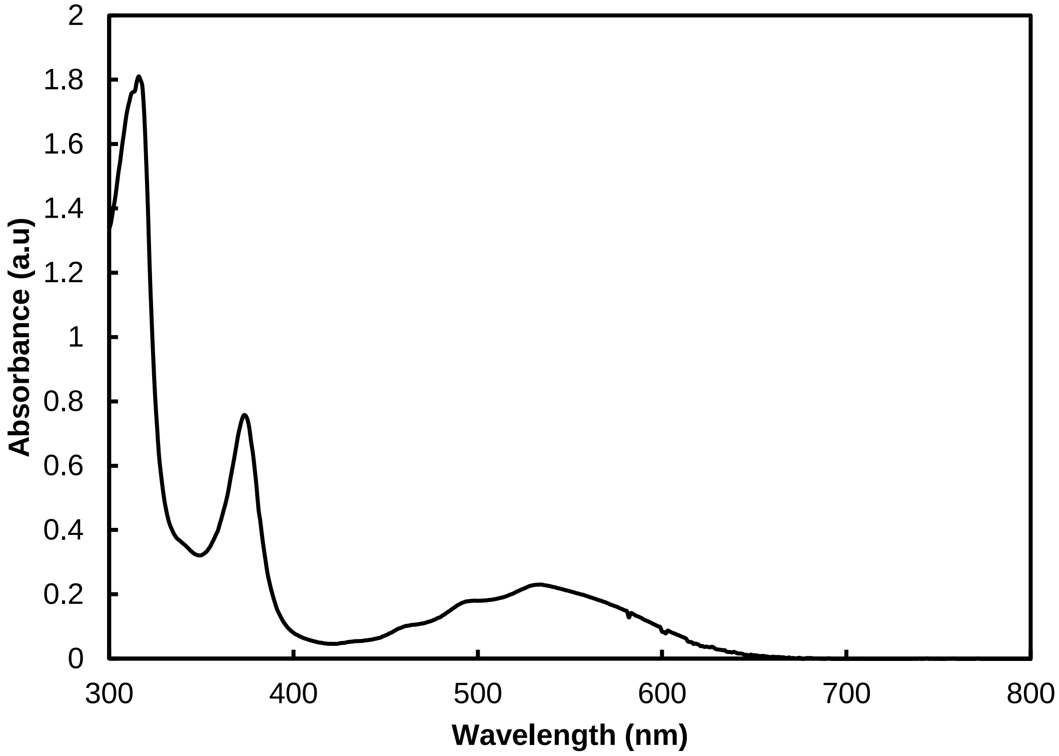

Figure S78. UV/Vis spectrum of **10** (4.3 x 10^{-5} M) in THF.

4. XRD Analyses

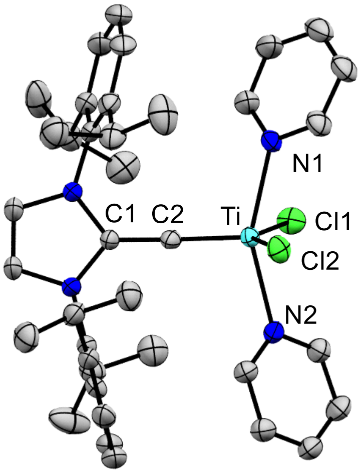

Figure S79. Molecular structure of compound **2** in the crystal. Thermal ellipsoids are depicted at 50% probability. Hydrogen atoms and solvent molecules are not shown. There are two independent molecules in the asymmetric unit, of which only one structure is shown. Selected bond lengths (Å) and angles (°) of both structures: C1–C2 1.388(4) and 1.393(4), C2–Ti 1.799(3) and 1.786(3), N1–Ti 2.256(3) and 2.266(4), N2–Ti 2.258(3) and 2.265(3), Cl1–Ti 2.379(1) and 2.372(1), Cl1–Ti 2.359(1) and 2.377(1), C1–C2–Ti 178.3(3) and 177.3(3).

Experimental

Single clear intense yellow plate-shaped crystals of **2** were used as supplied. A suitable crystal with dimensions 0.16 × 0.12 × 0.01 mm was selected and mounted on a SuperNova, Dual, Cu at home/near, AtlasS2 diffractometer. The crystal was kept at a steady *T* = 140.00(10) K during data collection. The structure was solved with the ShelXT 2018/2 (Sheldrick, 2015) solution program[4] using dual methods and by using Olex2 1.5 (Dolomanov et al., 2009)[5] as the graphical interface. The model was refined with ShelXL 2019/3 (Sheldrick, 2015)[6] using full matrix least squares minimisation on $|F|^2$.

Structure Quality Indicators

**Reflections:**	d min (CuKα) 2Θ=146.5° 0.81	I/σ(I) 12.6	Rint m=2.55 7.08%	Full 135.4° 97% to 146.5° 99.9
**Refinement:**	Shift 0.001	Max Peak 0.9	Min Peak -0.9	GooF 1.021

A clear intense yellow plate-shaped crystal with dimensions 0.16 × 0.12 × 0.01 mm was mounted. Data were collected using a SuperNova, Dual, Cu at home/near, AtlasS2 diffractometer operating

at T = 140.00(10) K.

Data were measured using w scans with Cu K_{α} radiation. The diffraction pattern was indexed and the total number of runs and images was based on the strategy calculation from the program CrysAlis^{Pro} system (CCD 44.112a 64-bit (release 21-05-2025)).[7] The maximum resolution achieved was Θ = 73.266°.

The unit cell was refined using CrysAlis^{Pro} on 6515 reflections, 19% of the observed reflections.

Data reduction, scaling and absorption corrections were performed using CrysAlis^{Pro}. The final completeness is 99.90% out to 73.266° in Θ. A Gaussian absorption correction was performed using CrysAlis^{Pro} 1.171.44.111a (Rigaku Oxford Diffraction, 2025) Numerical absorption correction based on Gaussian integration over a multifaceted crystal model. Empirical absorption correction using spherical harmonics as implemented in SCALE3 ABSPACK scaling algorithm. The absorption coefficient μ of this material is 3.581 mm^{-1} at this wavelength (λ = 1.54184Å) and the minimum and maximum transmissions are 0.617 and 1.000.

The structure was solved in the space group $P2_1/c$ (# 14) by ShelXT 2018/2 (Sheldrick, 2015) using dual methods. It was refined by full matrix least squares minimisation on $\mathbf{|F|^2}$ using version 2019/3 of ShelXL 2019/3 (Sheldrick, 2015). All non-hydrogen atoms were refined anisotropically.

Hydrogen atom positions were calculated geometrically and refined using the riding model.

The value of Z' is 2. This means that there are two independent molecules in the asymmetric unit. The moiety formula is $C_{38}H_{46}Cl_2N_4Ti$.

Table S1. Crystal data and structure refinement for compound **2**.

**Compound**	**2**
Formula	$C_{38}H_{46}Cl_2N_4Ti$
$D_{calc.}$/ g cm^{-3}	1.239
m/mm^{-1}	3.581
Formula Weight	677.59
Colour	clear intense yellow
Shape	plate-shaped
Size/mm	0.16×0.12×0.01
T/K	140.00(10)
Crystal System	monoclinic
Space Group	$P2_1/c$
a/Å	31.2839(11)
b/Å	12.2128(4)
c/Å	19.4011(7)
α/°	90
β/°	101.445(4)
γ/°	90
V/Å^3	7265.1(4)
Z	8
Z'	2
Wavelength/Å	1.54184
Radiation type	Cu K_α
Θ_{min}/°	3.896
Θ_{max}/°	73.266
Index range h	-38 ≤ h ≤ 38
Index range k	-5 ≤ k ≤ 14
Index range l	-24 ≤ l ≤ 23
Measured Refl's.	35097
Indep't Refl's	14126
Refl's I≥2σ(I)	9962
R_{int}	0.0708
Parameters	827
Restraints	0
Largest Peak/eÅ^3	0.893
Deepest Hole/eÅ^3	-0.913
GooF	1.021
R_1 (I≥2σ(I) / all)	0.0634 / 0.0937
wR_2 (I≥2σ(I) / all)	0.1478 / 0.1677
CCDC number	2515158

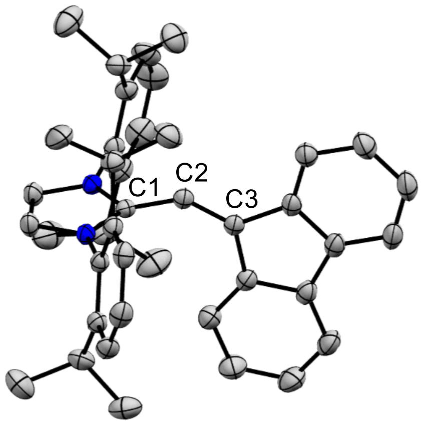

Figure S80. Molecular structure of compound **3** in the crystal. Thermal ellipsoids are depicted at 50% probability. Hydrogen atoms and solvent molecules are not shown. Selected bond lengths (Å) and angles (°): C1–C2 1.371(2), C2–C3 1.331(1), C1–C2–C3 138.0(1).

Experimental

Single clear dark purple prism-shaped crystals of **3** were used as supplied. A suitable crystal with dimensions 0.30 × 0.15 × 0.12 mm was selected and mounted on a SuperNova, Dual, Cu at home/near, AtlasS2 diffractometer. The crystal was kept at a steady T = 140.00(10) K during data collection. The structure was solved with the ShelXT (Sheldrick, 2015) solution program using dual methods and by using Olex2 1.5 (Dolomanov et al., 2009) as the graphical interface. The model was refined with ShelXL 2019/3 (Sheldrick, 2015) using full matrix least squares minimisation on $|F|^2$.

Structure Quality Indicators

**Reflections:**	d min (CuKα) 2Θ=145.5° 0.81	I/σ(I) 32.3	Rint m=2.05 2.33%	Full 135.4° 97% to 145.5° 99.8
**Refinement:**	Shift 0.000	Max Peak 0.3	Min Peak -0.2	GooF 1.025

A clear dark purple prism-shaped crystal with dimensions 0.30 × 0.15 × 0.12 mm was mounted. Data were collected using a SuperNova, Dual, Cu at home/near, AtlasS2 diffractometer operating at T = 140.00(10) K.

Data were measured using ω scans with Cu K_α radiation. The diffraction pattern was indexed and the total number of runs and images was based on the strategy calculation from the program CrysAlis^{Pro} system (CCD 44.120a 64-bit (release 22-08-2025)). The maximum resolution achieved was Θ = 72.741° (0.81 Å).

The unit cell was refined using CrysAlis^{Pro} on 9944 reflections, 60% of the observed reflections.

Data reduction, scaling and absorption corrections were performed using CrysAlis^{Pro}. The final completeness is 99.80 % out to 72.741° in Θ. An analytical absorption correction was performed using CrysAlis^{Pro} 1.171.44.120a (Rigaku Oxford Diffraction, 2025). The analytical numeric absorption correction using a multifaceted crystal model based on expressions derived by R.C. Clark & J.S. Reid. (Clark, R. C. & Reid, J. S. (1995). Acta Cryst. A51, 887-897). The empirical absorption correction was done using spherical harmonics, implemented in SCALE3 ABSPACK scaling algorithm. The absorption coefficient μ of this crystal is 0.455 mm^{-1} at this wavelength (λ = 1.54184Å) and the minimum and maximum transmissions are 0.901 and 0.962.

The structure was solved in the space group *P*-1 (# 2) by ShelXT (Sheldrick, 2015) using dual methods. It was refined by full matrix least squares minimisation on $|F|^2$ using version 2019/3 of ShelXL 2019/3 (Sheldrick, 2015). All non-hydrogen atoms were refined anisotropically.

Most hydrogen atom positions were calculated geometrically and refined using the riding model, but some hydrogen atoms were refined freely.

The value of Z' is 0.5. This means that only half of the formula unit is present in the asymmetric unit, with the other half consisting of symmetry equivalent atoms. The moiety formula is $2(C_{41}H_{44}N_2)$, $3(C_5H_{12})$.

Table S2. Crystal data and structure refinement for compound **3**.

**Compound**	**3**
Formula	$C_{97}H_{124}N_4$
$D_{calc.}$/ g cm^{-3}	1.072
μ/mm^{-1}	0.455
Formula Weight	1345.99
Colour	clear dark purple
Shape	prism-shaped
Size/mm	0.30×0.15×0.12
T/K	140.00(10)
Crystal System	triclinic
Space Group	*P*-1
a/Å	11.1069(3)
b/Å	12.6780(3)
c/Å	16.9186(5)
α/°	88.575(2)
β/°	70.940(3)
γ/°	68.662(2)
V/Å^3	2085.42(11)
Z	1
Z'	0.5
Wavelength/Å	1.54184
Radiation type	Cu K_{α}
Θ_{min}/°	3.765
Θ_{max}/°	72.741
Index range h	-13 ≤ h ≤ 13
Index range k	-15 ≤ k ≤ 10
Index range l	-20 ≤ l ≤ 20
Measured Refl's.	16538
Indep't Refl's	8080
Refl's I≥2σ(I)	7210
R_{int}	0.0233
Parameters	707
Restraints	239
Largest Peak/eÅ^3	0.298
Deepest Hole/eÅ^3	-0.251
GooF	1.025
R_1 (I≥2σ(I) / all)	0.0428 / 0.0478
wR_2 (I≥2σ(I) / all)	0.1108 / 0.1160
CCDC number	2516492

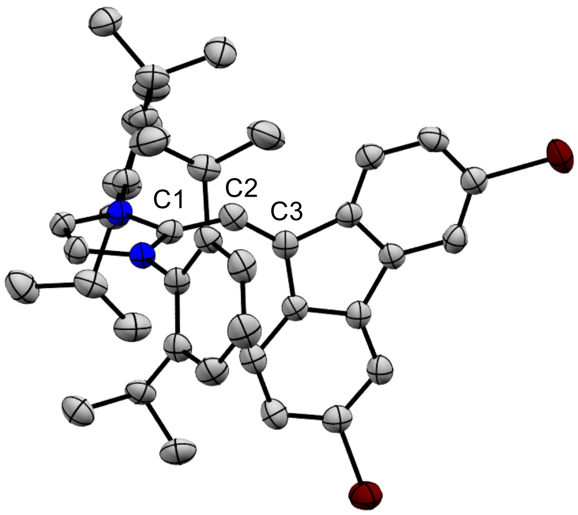

Figure S81. Molecular structure of compound **4** in the crystal. Thermal ellipsoids are depicted at 50% probability. Hydrogen atoms and solvent molecules are not shown. Selected bond lengths (Å) and angles (°): C1–C2 1.384(5), C2–C3 1.323(5), C1–C2–C3 138.7(4).

Experimental

Single clear light-purple plate-shaped crystals of **4** were used as supplied. A suitable crystal with dimensions 0.19 × 0.11 × 0.01 mm was selected and mounted on a XtaLAB Synergy R, DW system, HyPix-Arc 150 diffractometer. The crystal was kept at a steady T = 140.00(10) K during data collection. The structure was solved with the ShelXT 2018/2 (Sheldrick, 2015) solution program using dual methods and by using Olex2 1.5 (Dolomanov et al., 2009) as the graphical interface. The model was refined with ShelXL 2019/3 (Sheldrick, 2015) using full matrix least squares minimisation on $|F|^2$.

Structure Quality Indicators

**Reflections:**	d min (CuKα) 2Θ=151.5° .cif 0.80	I/σ(I) .cif 17.2	Rint m=4.57 .cif 6.37%	Full 135.4° 96% to 151.5° .cif 99.7
**Refinement:**	Shift .cif 0.001	Max Peak .cif 0.7	Min Peak .cif -0.8	GooF .cif 1.023

A clear light-purple plate-shaped crystal with dimensions 0.19 × 0.11 × 0.01 mm was mounted. Data were collected using a XtaLAB Synergy R, DW system, HyPix-Arc 150 diffractometer operating at T = 140.00(10) K.

Data were measured using ω scans with Cu K_α radiation. The diffraction pattern was indexed and

the total number of runs and images was based on the strategy calculation from the program CrysAlis^{Pro} system (CCD 44.122a 64-bit (release 14-09-2025)). The maximum resolution achieved was $\Theta = 75.767^{\circ}$.

The unit cell was refined using CrysAlis^{Pro} on 8251 reflections, 26% of the observed reflections.

Data reduction, scaling and absorption corrections were performed using CrysAlis^{Pro}. The final completeness is 99.70 % out to 75.767° in Θ. A Gaussian absorption correction was performed using CrysAlis^{Pro} 1.171.44.120a (Rigaku Oxford Diffraction, 2025) Numerical absorption correction based on Gaussian integration over a multifaceted crystal model. Empirical absorption correction using spherical harmonics as implemented in SCALE3 ABSPACK scaling algorithm. The absorption coefficient μ of this material is 3.137 mm^{-1} at this wavelength (λ = 1.54184 Å) and the minimum and maximum transmissions are 0.562 and 1.000.

The structure was solved in the space group *C*2/*c* (# 15) by ShelXT 2018/2 (Sheldrick, 2015) using dual methods. It was refined by full matrix least squares minimisation on $|F|^2$ using version 2019/3 of ShelXL 2019/3 (Sheldrick, 2015). All non-hydrogen atoms were refined anisotropically.

Hydrogen atom positions were calculated geometrically and refined using the riding model.

There is a single formula unit in the asymmetric unit, which is represented by the reported sum formula. In other words: Z is 8 and Z' is 1. The moiety formula is $C_{41}H_{42}Br_2N_2$.

Table S3. Crystal data and structure refinement for compound **4**.

**Compound**	**4**
Formula	$C_{41}H_{42}Br_2N_2$
$D_{calc.}$/ g cm^{-3}	1.361
m/mm^{-1}	3.137
Formula Weight	722.58
Colour	clear light-purple
Shape	plate-shaped
Size/mm	0.19×0.11×0.01
T/K	140.00(10)
Crystal System	monoclinic
Space Group	*C*2/*c*
a/Å	19.5321(6)
b/Å	16.7709(5)
c/Å	21.6276(4)
α/°	90
β/°	95.339(2)
γ/°	90
V/Å^3	7053.9(3)
Z	8
Z'	1
Wavelength/Å	1.54184
Radiation type	Cu K_α
Θ_{min}/°	3.480
Θ_{max}/°	75.767
Index range h	-24 ≤ h ≤ 24
Index range k	-20 ≤ k ≤ 20
Index range l	-26 ≤ l ≤ 21
Measured Refl's.	31157
Indep't Refl's	7010
Refl's I≥2σ(I)	4709
R_{int}	0.0637
Parameters	414
Restraints	0
Largest Peak/eÅ^3	0.736
Deepest Hole/eÅ^3	-0.842
GooF	1.023
R_1 (I≥2σ(I) / all)	0.0533 / 0.0901
wR_2 (I≥2σ(I) / all)	0.1270 / 0.1446
CCDC number	2515161

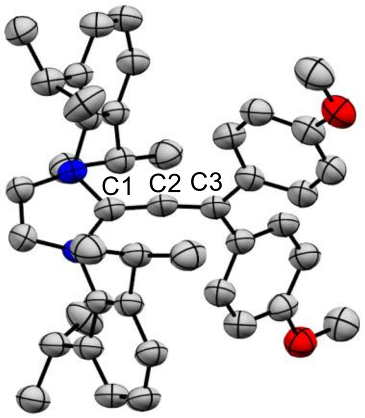

Figure S82. Molecular structure of compound **6** in the crystal. Thermal ellipsoids are depicted at 50% probability. Hydrogen atoms and solvent molecules are not shown. Selected bond lengths (Å) and angles (°): C1–C2 1.313(6), C2–C3 1.326(6), C1–C2–C3 172.1(4).

Experimental

Single clear light-orange plate-shaped crystals of **6** were used as supplied. A suitable crystal with dimensions 0.23 × 0.08 × 0.05 mm was selected and mounted on a XtaLAB Synergy R, DW system, HyPix-Arc 150 diffractometer. The crystal was kept at a steady *T* = 140.00(10) K during data collection. The structure was solved with the ShelXT (Sheldrick, 2015) solution program using dual methods and by using Olex2 1.5 (Dolomanov et al., 2009) as the graphical interface. The model was refined with ShelXL 2019/3 (Sheldrick, 2015) using full matrix least squares minimisation on $|F|^2$.

Structure Quality Indicators

**Reflections:**	d min (CuKα) 2Θ=133.2° 0.84	I/σ(I) 9.5	Rint m=1.65 35.98%	Full 133.2° 100
**Refinement:**	Shift 0.000	Max Peak 0.3	Min Peak -0.4	GooF 1.059

A clear light-orange plate-shaped crystal with dimensions 0.23 × 0.08 × 0.05 mm was mounted. Data were collected using a XtaLAB Synergy R, DW system, HyPix-Arc 150 diffractometer operating at *T* = 140.00(10) K.

Data were measured using ω scans with Cu K_{α} radiation. The diffraction pattern was indexed and the total number of runs and images was based on the strategy calculation from the program CrysAlis^{Pro} system (CCD 44.118a 64-bit (release 22-07-2025)). The maximum resolution

achieved was Θ = 66.599° (0.84 Å).

The unit cell was refined using CrysAlis^{Pro} on 5774 reflections, 78% of the observed reflections.

Data reduction, scaling and absorption corrections were performed using CrysAlis^{Pro}. The final completeness is 100.00 % out to 66.599° in Θ. An analytical absorption correction was performed using CrysAlis^{Pro} 1.171.44.118a (Rigaku Oxford Diffraction, 2025). The analytical numeric absorption correction was done using a multifaceted crystal model based on expressions derived by R.C. Clark & J.S. Reid. (Clark, R. C. & Reid, J. S. (1995). Acta Cryst. A51, 887-897). The empirical absorption correction was done using spherical harmonics, implemented in SCALE3 ABSPACK scaling algorithm. The absorption coefficient μ of this crystal is 0.547 mm^{-1} at this wavelength (λ = 1.54184Å) and the minimum and maximum transmissions are 0.911 and 0.979.

The structure was solved in the space group $P2_1/c$ (# 14) by ShelXT (Sheldrick, 2015) using dual methods. It was refined by full matrix least squares minimisation on $|\boldsymbol{F}|^2$ using version 2019/3 of ShelXL 2019/3 (Sheldrick, 2015). All non-hydrogen atoms were refined anisotropically.

Hydrogen atom positions were calculated geometrically and refined using the riding model.

_refine_special_details: Refined as a 2-component twin.

_twin_special_details: Component 2 rotated by 179.9555° around [-0.00 -0.00 1.00] (reciprocal) or [0.14 0.00 0.99] (direct)

There is a single formula unit in the asymmetric unit, which is represented by the reported sum formula. In other words: Z is 4 and Z' is 1. The moiety formula is $C_{43}H_{50}N_2O_2$.

Table S4. Crystal data and structure refinement for compound **6**.

**Compound**	**6**
Formula	$C_{43}H_{50}N_2O_2$
$D_{calc.}$/ g cm^{-3}	1.173
μ/mm^{-1}	0.547
Formula Weight	626.85
Colour	clear light orange
Shape	plate-shaped
Size/mm	0.23×0.08×0.05
T/K	140.00(10)
Crystal System	monoclinic
Space Group	$P2_1/c$
a/Å	10.1004(5)
b/Å	17.1917(9)
c/Å	20.4839(10)
α/°	90
β/°	94.030(5)
γ/°	90
V/Å^3	3548.1(3)
Z	4
Z'	1
Wavelength/Å	1.54184
Radiation type	Cu K_α
Θ_{min}/°	3.360
Θ_{max}/°	66.599
Index range h	$-12 \le h \le 12$
Index range k	$-20 \le k \le 20$
Index range l	$-23 \le l \le 24$
Measured Refl's.	7369
Indep't Refl's	7369
Refl's I≥2σ(I)	4874
R_{int}	.
Parameters	436
Restraints	0
Largest Peak/eÅ^3	0.282
Deepest Hole/eÅ^3	-0.357
GooF	1.059
R_1 (I≥2σ(I) / all)	0.0976 / 0.1359
wR_2 (I≥2σ(I) / all)	0.2609 / 0.2894
CCDC number	2516491

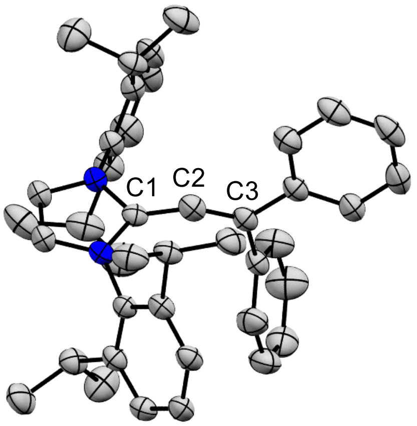

Figure S83. Molecular structure of compound **7** in the crystal. Thermal ellipsoids are depicted at 50% probability. Hydrogen atoms and solvent molecules are not shown. Selected bond lengths (Å) and angles (°): C1–C2 1.341(4), C2–C3 1.324(4), C1–C2–C3 152.3(3).

Experimental

Single orange irregular-shaped crystals of **7** were used as supplied. A suitable crystal with dimensions 0.22 × 0.16 × 0.06 mm was selected and mounted on a SuperNova, Dual, Cu at home/near, AtlasS2 diffractometer. The crystal was kept at a steady T = 140.00(10) K during data collection. The structure was solved with the ShelXT (Sheldrick, 2015) solution program using dual methods and by using Olex2 1.5 (Dolomanov et al., 2009) as the graphical interface. The model was refined with ShelXL 2019/3 (Sheldrick, 2015) using full matrix least squares minimisation on $|F|^2$.

Structure Quality Indicators

**Reflections:**	d min (CuKα) 2Θ=145.4° 0.81	I/σ(I) 14.0	Rint m=1.58 12.53%	Full 135.4° 97% to 145.4° 99.9
**Refinement:**	Shift 0.000	Max Peak 0.2	Min Peak -0.2	GooF 0.924

An orange irregular-shaped crystal with dimensions 0.22 × 0.16 × 0.06 mm was mounted. Data were collected using a SuperNova, Dual, Cu at home/near, AtlasS2 diffractometer operating at T = 140.00(10) K.

Data were measured using ω scans with Cu K_α radiation. The diffraction pattern was indexed and the total number of runs and images was based on the strategy calculation from the program

CrysAlis^{Pro} system (CCD 44.113a 64-bit (release 02-06-2025)). The maximum resolution achieved was $\Theta = 72.683^{\circ}$ (0.81 Å).

The unit cell was refined using CrysAlis^{Pro} on 4422 reflections, 59% of the observed reflections.

Data reduction, scaling and absorption corrections were performed using CrysAlis^{Pro}. The final completeness is 99.90 % out to 72.683° in Θ. A gaussian absorption correction was performed using CrysAlis^{Pro} 1.171.44.113a (Rigaku Oxford Diffraction, 2025). The numerical absorption correction was based on gaussian integration over a multifaceted crystal model. The Empirical absorption correction was using spherical harmonics, implemented in SCALE3 ABSPACK scaling algorithm. The absorption coefficient μ of this crystal is 0.484 mm^{-1} at this wavelength (λ = 1.54184Å) and the minimum and maximum transmissions are 0.607 and 1.000.

The structure was solved in the space group $P2_1/c$ (# 14) by ShelXT (Sheldrick, 2015) using dual methods. It was refined by full matrix least squares minimisation on $|F|^2$ using version 2019/3 of ShelXL 2019/3 (Sheldrick, 2015). All non-hydrogen atoms were refined anisotropically.

Hydrogen atom positions were calculated geometrically and refined using the riding model.

_refine_special_details: Refined as a 2-component twin.

_twin_special_details: Component 2 rotated by -179.8297° around [1.00 0.00 0.00] (reciprocal) or [1.00 0.00 0.09] (direct)

There is a single formula unit in the asymmetric unit, which is represented by the reported sum formula. In other words: Z is 4 and Z' is 1. The moiety formula is $C_{41}H_{46}N_2$.

Table S5. Crystal data and structure refinement for compound **7**.

**Compound**	**7**
Formula	$C_{41}H_{46}N_2$
$D_{calc.}$/ g cm^{-3}	1.121
μ/mm^{-1}	0.484
Formula Weight	566.80
Colour	orange
Shape	irregular-shaped
Size/mm	0.22×0.16×0.06
T/K	140.00(10)
Crystal System	monoclinic
Space Group	$P2_1/c$
a/Å	13.2291(4)
b/Å	13.2352(4)
c/Å	19.3429(8)
α/°	90
β/°	97.347(3)
γ/°	90
V/Å^3	3358.9(2)
Z	4
Z'	1
Wavelength/Å	1.54184
Radiation type	Cu K_α
Θ_{min}/°	3.368
Θ_{max}/°	72.683
Index range h	-16 ≤ h ≤ 16
Index range k	-16 ≤ k ≤ 16
Index range l	-23 ≤ l ≤ 23
Measured Refl's.	7517
Indep't Refl's	7517
Refl's I≥2σ(I)	4350
R_{int}	.
Parameters	397
Restraints	0
Largest Peak/eÅ^3	0.211
Deepest Hole/eÅ^3	-0.204
GooF	0.924
R_1 (I≥2σ(I) / all)	0.0638 / 0.1032
wR_2 (I≥2σ(I) / all)	0.1649 / 0.1801
CCDC number	2516490

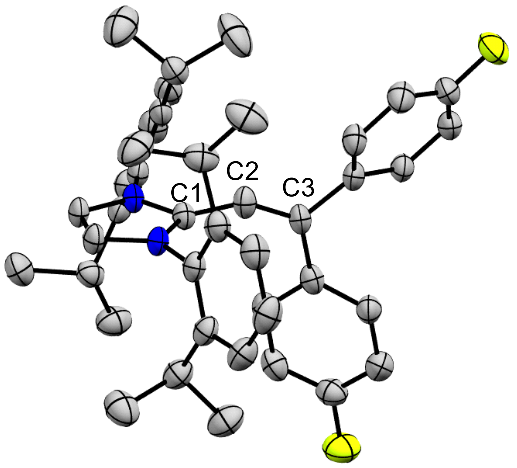

Figure S84. Molecular structure of compound **8** in the crystal. Thermal ellipsoids are depicted at 50% probability. Hydrogen atoms and solvent molecules are not shown. Selected bond lengths (Å) and angles (°): C1–C2 1.358(3), C2–C3 1.322(3), C1–C2–C3 149.2(2).

Experimental

Single clear light-orange plate-shaped crystals of **8** were used as supplied. A suitable crystal with dimensions 0.16 × 0.07 × 0.02 mm was selected and mounted on a XtaLAB Synergy R, DW system, HyPix-Arc 150 diffractometer. The crystal was kept at a steady T = 140.00(10) K during data collection. The structure was solved with the ShelXT 2018/2 (Sheldrick, 2015) solution program using dual methods and by using Olex2 1.5 (Dolomanov et al., 2009) as the graphical interface. The model was refined with ShelXL 2019/3 (Sheldrick, 2015) using full matrix least squares minimisation on $|F|^2$.

Structure Quality Indicators

**Reflections:**	d min (CuKα) 2Θ=148.3° .cif 0.80	I/σ(I) .cif 15.9	Rint m=5.46 .cif 6.90%	Full 135.4° 96% to 148.3° .cif 99.3
**Refinement:**	Shift .cif 0.000	Max Peak .cif 0.2	Min Peak .cif -0.3	GooF .cif 1.039

A clear light-orange plate-shaped crystal with dimensions 0.16 × 0.07 × 0.02 mm was mounted. Data were collected using a XtaLAB Synergy R, DW system, HyPix-Arc 150 diffractometer operating at T = 140.00(10) K.

Data were measured using ω scans with Cu K_α radiation. The diffraction pattern was indexed and

the total number of runs and images was based on the strategy calculation from the program CrysAlis^{Pro} system (CCD 44.118a 64-bit (release 22-07-2025)). The maximum resolution achieved was $\Theta = 74.143^{\circ}$.

The unit cell was refined using CrysAlis^{Pro} on 6639 reflections, 19% of the observed reflections.

Data reduction, scaling and absorption corrections were performed using CrysAlis^{Pro}. The final completeness is 99.30 % out to 74.143° in Θ. A Gaussian absorption correction was performed using CrysAlis^{Pro} 1.171.44.118a (Rigaku Oxford Diffraction, 2025) Numerical absorption correction based on Gaussian integration over a multifaceted crystal model. Empirical absorption correction using spherical harmonics as implemented in SCALE3 ABSPACK scaling algorithm. The absorption coefficient μ of this material is 0.594 mm^{-1} at this wavelength (λ = 1.54184Å) and the minimum and maximum transmissions are 0.750 and 1.000.

The structure was solved in the space group *C*2/*c* (# 15) by ShelXT 2018/2 (Sheldrick, 2015) using dual methods. It was refined by full matrix least squares minimisation on $|F|^2$ using version 2019/3 of ShelXL 2019/3 (Sheldrick, 2015). All non-hydrogen atoms were refined anisotropically.

Hydrogen atom positions were calculated geometrically and refined using the riding model.

There is a single formula unit in the asymmetric unit, which is represented by the reported sum formula. In other words: Z is 8 and Z' is 1. The moiety formula is $C_{41}H_{44}F_2N_2$.

Table S6. Crystal data and structure refinement for compound **8**.

**Compound**	**8**
Formula	$C_{41}H_{44}F_2N_2$
$D_{calc.}$/ g cm^{-3}	1.175
m/mm^{-1}	0.594
Formula Weight	602.78
Colour	clear light orange
Shape	plate-shaped
Size/mm	0.16×0.07×0.02
T/K	140.00(10)
Crystal System	monoclinic
Space Group	$C2/c$
a/Å	17.3155(4)
b/Å	19.9888(6)
c/Å	19.7101(3)
α/°	90
β/°	92.7776(19)
γ/°	90
V/Å^3	6814.0(3)
Z	8
Z'	1
Wavelength/Å	1.54184
Radiation type	Cu K_α
Θ_{min}/°	3.379
Θ_{max}/°	74.143
Index range h	-21 ≤ h ≤ 21
Index range k	-24 ≤ k ≤ 24
Index range l	-12 ≤ l ≤ 23
Measured Refl's.	35587
Indep't Refl's	6675
Refl's I≥2σ(I)	4183
R_{int}	0.0690
Parameters	414
Restraints	0
Largest Peak/eÅ^3	0.183
Deepest Hole/eÅ^3	-0.333
GooF	1.039
R_1 (I≥2σ(I) / all)	0.0546 / 0.0990
wR_2 (I≥2σ(I) / all)	0.1295 / 0.1517
CCDC number	2515160

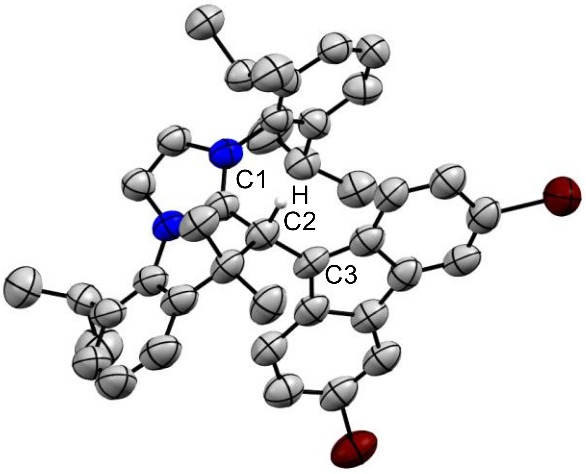

Figure S85. Molecular structure of compound **10** in the crystal. Thermal ellipsoids are depicted at 50% probability. Hydrogen atoms and solvent molecules are not shown. There are two independent molecules in the asymmetric unit, of which only one structure is shown. Selected bond lengths (Å) and angles (°) of both structures: C1–C2 1.499(7) and 1.469(9), C2–C3 1.508(9) and 1.527(9), C2–H 1.000 and 1.000, C1–C2–C3 113.1(5) and 112.1(5).

Experimental

Single clear intense orange irregular-shaped crystals of **10** were used as supplied. A suitable crystal with dimensions 0.33 × 0.08 × 0.06 mm was selected and mounted on a XtaLAB Synergy R, DW system, HyPix-Arc 150 diffractometer. The crystal was kept at a steady T = 200.0(6) K during data collection. The structure was solved with the ShelXT (Sheldrick, 2015) solution program using dual methods and by using Olex2 1.5 (Dolomanov et al., 2009) as the graphical interface. The model was refined with ShelXL 2019/3 (Sheldrick, 2015) using full matrix least squares minimisation on $|F|^2$.

Structure Quality Indicators

**Reflections:**	d min (CuKα) 2Θ=133.2° 0.84	I/σ(I) 11.1	Rint m=9.92 17.37%	Full 133.2° 99.7
**Refinement:**	Shift 0.001	Max Peak 0.5	Min Peak -0.7	GooF 1.002

A clear intense orange irregular-shaped crystal with dimensions 0.33 × 0.08 × 0.06 mm was mounted. Data were collected using a XtaLAB Synergy R, DW system, HyPix-Arc 150 diffractometer operating at T = 200.0(6) K.

Data were measured using ω scans with Cu K_α radiation. The diffraction pattern was indexed and the total number of runs and images was based on the strategy calculation from the program CrysAlis^{Pro} system (CCD 44.122a 64-bit (release 14-09-2025)). The maximum resolution achieved was Θ = 66.600° (0.84 Å).

The unit cell was refined using CrysAlis^{Pro} on 15989 reflections, 12% of the observed reflections.

Data reduction, scaling and absorption corrections were performed using CrysAlis^{Pro}. The final completeness is 99.70 % out to 66.600° in Θ. A gaussian absorption correction was performed using CrysAlis^{Pro} 1.171.44.122a (Rigaku Oxford Diffraction, 2025). The numerical absorption correction was based on gaussian integration over a multifaceted crystal model. The empirical absorption correction was done using spherical harmonics, implemented in SCALE3 ABSPACK scaling algorithm. The absorption coefficient μ of this crystal is 2.951 mm^{-1} at this wavelength (λ = 1.54184Å) and the minimum and maximum transmissions are 0.545 and 1.000.

The structure was solved in the space group $P2_1/c$ (# 14) by ShelXT (Sheldrick, 2015) using dual methods. It was refined by full matrix least squares minimisation on $|F|^2$ using version 2019/3 of ShelXL 2019/3 (Sheldrick, 2015). All non-hydrogen atoms were refined anisotropically.

Hydrogen atom positions were calculated geometrically and refined using the riding model.

There is a single formula unit in the asymmetric unit, which is represented by the reported sum formula. In other words: Z is 4 and Z' is 1. The moiety formula is $2(C_{41}H_{42}Br_2N_2)$, C_7H_8.

Table S7. Crystal data and structure refinement for compound **10**.

**Compound**	**10**
Formula	$C_{89}H_{92}Br_4N_4$
$D_{calc.}$/ g cm^{-3}	1.346
μ/mm^{-1}	2.951
Formula Weight	1537.30
Colour	clear intense orange
Shape	irregular-shaped
Size/mm	0.33×0.08×0.06
T/K	200.0(6)
Crystal System	monoclinic
Space Group	$P2_1/c$
a/Å	19.8266(11)
b/Å	22.0027(14)
c/Å	17.9365(8)
α/°	90
β/°	104.211(5)
γ/°	90
V/Å^3	7585.1(7)
Z	4
Z'	1
Wavelength/Å	1.54184
Radiation type	Cu K_α
Θ_{min}/°	2.299
Θ_{max}/°	66.600
Index range h	-23 ≤ h ≤ 23
Index range k	-26 ≤ k ≤ 26
Index range l	-21 ≤ l ≤ 19
Measured Refl's.	129826
Indep't Refl's	13367
Refl's I≥2σ(I)	6283
R_{int}	0.1737
Parameters	903
Restraints	19
Largest Peak/eÅ^3	0.516
Deepest Hole/eÅ^3	-0.681
GooF	1.002
R_1 (I≥2σ(I) / all)	0.0692 / 0.1559
wR_2 (I≥2σ(I) / all)	0.1631 / 0.2163
CCDC number	2516493

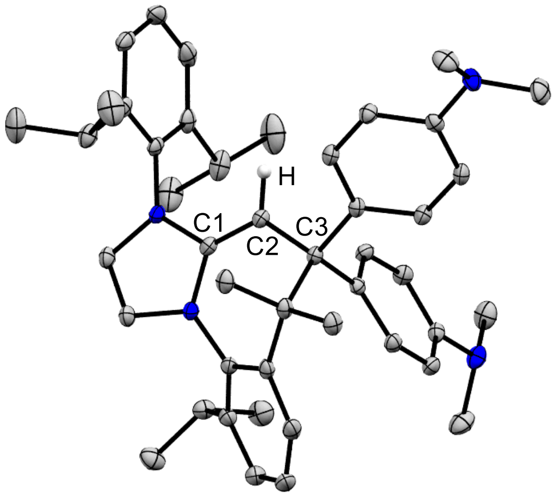

Figure S86. Molecular structure of compound **11** in the crystal. Thermal ellipsoids are depicted at 50% probability. Most hydrogen atoms and all solvent molecules are not shown. Selected bond lengths (Å) and angles (°): C1–C2 1.344(2), C2–C3 1.514(1), C2–H 0.98(1), C1–C2–C3 124.1(1).

Experimental

Single clear light-yellow hexagonal-shaped crystals of **11** were used as supplied. A suitable crystal with dimensions 0.26 × 0.19 × 0.13 mm was selected and mounted on a XtaLAB Synergy R, DW system, HyPix-Arc 150 diffractometer. The crystal was kept at a steady *T* = 100.00(10) K during data collection. The structure was solved with the ShelXT 2018/2 (Sheldrick, 2015) solution program using dual methods and by using Olex2 1.5 (Dolomanov et al., 2009) as the graphical interface. The model was refined with ShelXL 2019/3 (Sheldrick, 2015) using full matrix least squares minimisation on **|*F*|²**.

Structure Quality Indicators

**Reflections:**	d min (CuKα) 2Θ=151.4° 0.80	I/σ(I) 44.6	Rint m=5.83 2.89%	Full 135.4° 99% to 151.4° 100
**Refinement:**	Shift 0.000	Max Peak 0.3	Min Peak -0.2	GooF 1.042

A clear light-yellow hexagonal-shaped crystal with dimensions 0.26 × 0.19 × 0.13 mm was mounted. Data were collected using a XtaLAB Synergy R, DW system, HyPix-Arc 150 diffractometer operating at *T* = 100.00(10) K.

Data were measured using ω scans with Cu K_{α} radiation. The diffraction pattern was indexed and

the total number of runs and images was based on the strategy calculation from the program CrysAlis^{Pro} system (CCD 44.125a 64-bit (release 17-10-2025)). The maximum resolution achieved was $\Theta = 75.715^{\circ}$.

The unit cell was refined using CrysAlis^{Pro} on 10735 reflections, 24% of the observed reflections.

Data reduction, scaling and absorption corrections were performed using CrysAlis^{Pro}. The final completeness is 100.00% out to 75.715° in Θ. A Gaussian absorption correction was performed using CrysAlis^{Pro} 1.171.44.128a (Rigaku Oxford Diffraction, 2025) Numerical absorption correction based on Gaussian integration over a multifaceted crystal model. Empirical absorption correction using spherical harmonics as implemented in SCALE3 ABSPACK scaling algorithm. The absorption coefficient μ of this material is 0.507 mm^{-1} at this wavelength (λ = 1.54184Å) and the minimum and maximum transmissions are 0.700 and 1.000.

The structure was solved in the space group $P2_1/c$ (# 14) by ShelXT 2018/2 (Sheldrick, 2015) using dual methods. It was refined by full matrix least squares minimisation on $|\boldsymbol{F}|^2$ using version 2019/3 of ShelXL 2019/3 (Sheldrick, 2015). All non-hydrogen atoms were refined anisotropically.

All hydrogen atoms were freely refined.

There is a single formula unit in the asymmetric unit, which is represented by the reported sum formula. In other words: Z is 4 and Z' is 1. The moiety formula is $C_{45}H_{56}N_4$.

Table S8. Crystal data and structure refinement for compound **11**.

**Compound**	**11**
Formula	$C_{45}H_{56}N_4$
$D_{calc.}$/ g cm^{-3}	1.152
m/mm^{-1}	0.507
Formula Weight	652.93
Colour	clear light yellow
Shape	hexagonal-shaped
Size/mm	0.26×0.19×0.13
T/K	100.00(10)
Crystal System	monoclinic
Space Group	$P2_1/c$
a/Å	10.01431(19)
b/Å	20.8507(3)
c/Å	18.6608(3)
$\alpha/^{\circ}$	90
$\beta/^{\circ}$	104.8941(19)
$\gamma/^{\circ}$	90
V/Å^3	3765.56(12)
Z	4
Z'	1
Wavelength/Å	1.54184
Radiation type	Cu K_{α}
$\Theta_{min}/^{\circ}$	3.240
$\Theta_{max}/^{\circ}$	75.715
Index range h	-12 ≤ h ≤ 12
Index range k	-25 ≤ k ≤ 25
Index range l	-23 ≤ l ≤ 22
Measured Refl's.	44233
Indep't Refl's	7727
Refl's I≥2σ(I)	6728
R_{int}	0.0289
Parameters	667
Restraints	0
Largest Peak/eÅ^3	0.252
Deepest Hole/eÅ^3	-0.196
GooF	1.042
R_1 (I≥2σ(I) / all)	0.0361 / 0.0428
wR_2 (I≥2σ(I) / all)	0.0859 / 0.0889
CCDC number	2515159

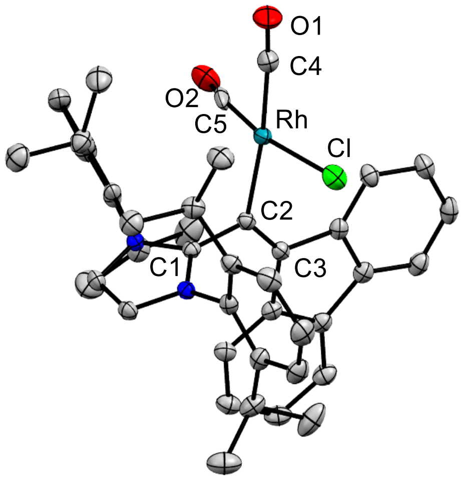

Figure S87. Molecular structure of compound **13** in the crystal. Thermal ellipsoids are depicted at 50% probability. Hydrogen atoms and solvent molecules are not shown. Selected bond lengths (Å) and angles (°): C1–C2 1.466(2), C2–C3 1.355(2), C2–Rh 2.093(2), C4–Rh 1.903(2), C5–Rh 1.933(2), C4–O1 1.129(3), C5–O2 0.995(3), Cl–Rh 2.3607(6), C1–C2–C3 119.4(1).

Experimental

Single clear intense yellow irregular-shaped crystals of **13** were used as supplied. A suitable crystal with dimensions 0.35 × 0.14 × 0.10 mm was selected and mounted on a SuperNova, Dual, Cu at home/near, Atlas diffractometer. The crystal was kept at a steady T = 140.00(10) K during data collection. The structure was solved with the ShelXT (Sheldrick, 2015) solution program using dual methods and by using Olex2 1.5 (Dolomanov et al., 2009) as the graphical interface. The model was refined with ShelXL 2019/3 (Sheldrick, 2015) using full matrix least squares minimisation on $|F|^2$.

Structure Quality Indicators

**Reflections:**	d min (CuKα) 2Θ=153.3° 0.79	I/σ(I) 44.7	Rint m=3.19 2.29%	Full 135.4° 99% to 153.3° 100
**Refinement:**	Shift -0.002	Max Peak 0.8	Min Peak -0.7	GooF 1.036

A clear intense yellow irregular-shaped crystal with dimensions 0.35 × 0.14 × 0.10 mm was mounted. Data were collected using a SuperNova, Dual, Cu at home/near, Atlas diffractometer operating at T = 140.00(10) K.

Data were measured using ω scans with Cu K_{α} radiation. The diffraction pattern was indexed and

the total number of runs and images was based on the strategy calculation from the program CrysAlis^{Pro} system (CCD 44.122a 64-bit (release 14-09-2025)). The maximum resolution achieved was $\Theta = 76.651^{\circ}$ (0.79 Å).

The unit cell was refined using CrysAlis^{Pro} on 19938 reflections, 66% of the observed reflections.

Data reduction, scaling and absorption corrections were performed using CrysAlis^{Pro}. The final completeness is 100.00 % out to 76.651° in Θ. An analytical absorption correction was performed using CrysAlis^{Pro} 1.171.44.122a (Rigaku Oxford Diffraction, 2025). The analytical numeric absorption correction was done using a multifaceted crystal model based on expressions derived by R.C. Clark & J.S. Reid. (Clark, R. C. & Reid, J. S. (1995). Acta Cryst. A51, 887-897). The empirical absorption correction was done using spherical harmonics, implemented in SCALE3 ABSPACK scaling algorithm. The absorption coefficient μ of this crystal is 3.852 mm^{-1} at this wavelength (λ = 1.54184Å) and the minimum and maximum transmissions are 0.407 and 0.758.

The structure was solved in the space group $P2_1/c$ (# 14) by ShelXT (Sheldrick, 2015) using dual methods. It was refined by full matrix least squares minimisation on $|F|^2$ using version 2019/3 of ShelXL 2019/3 (Sheldrick, 2015). All non-hydrogen atoms were refined anisotropically.

Hydrogen atom positions were calculated geometrically and refined using the riding model.

There is a single formula unit in the asymmetric unit, which is represented by the reported sum formula. In other words: Z is 4 and Z' is 1. The moiety formula is $C_{43}H_{44}ClN_2O_2Rh$, $2(C_6H_6)$.

Table S9. Crystal data and structure refinement for complex **13**.

**Compound**	**13**
Formula	$C_{55}H_{56}ClN_2O_2Rh$
$D_{calc.}$/ g cm^{-3}	1.317
μ/mm^{-1}	3.852
Formula Weight	915.37
Colour	clear intense yellow
Shape	irregular-shaped
Size/mm	0.35×0.14×0.10
T/K	140.00(10)
Crystal System	monoclinic
Space Group	$P2_1/c$
a/Å	10.24591(10)
b/Å	25.6249(2)
c/Å	17.80191(17)
α/°	90
β/°	98.8775(9)
γ/°	90
V/Å^3	4617.91(8)
Z	4
Z'	1
Wavelength/Å	1.54184
Radiation type	Cu K_α
Θ_{min}/°	3.047
Θ_{max}/°	76.651
Index range h	-12 ≤ h ≤ 12
Index range k	-24 ≤ k ≤ 32
Index range l	-22 ≤ l ≤ 21
Measured Refl's.	30222
Indep't Refl's	9583
Refl's I≥2□(I)	8855
R_{int}	0.0229
Parameters	579
Restraints	0
Largest Peak/eÅ^3	0.785
Deepest Hole/eÅ^3	-0.711
GooF	1.036
R_1 (I≥2σ(I) / all)	0.0284 / 0.0313
wR_2 (I≥2σ(I) / all)	0.0724 / 0.0742
CCDC number	2516494

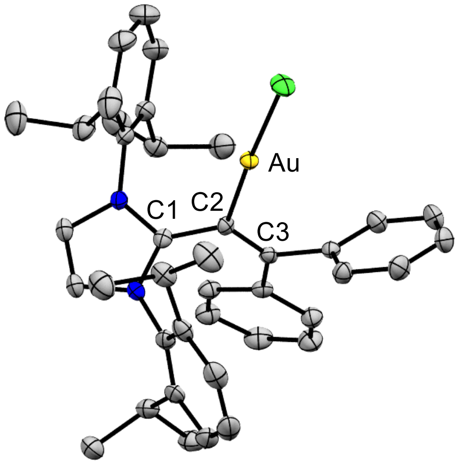

Figure S88. Molecular structure of compound **14** in the crystal. Thermal ellipsoids are depicted at 50% probability. Hydrogen atoms and solvent molecules are not shown. Selected bond lengths (Å) and angles (°): C1–C2 1.446(4), C2–C3 1.364(4), C2–Au 2.021(2), C1–C2–C3 122.5(2).

Experimental

Single clear light-orange irregular-shaped crystals of **14** were used as supplied. A suitable crystal with dimensions 0.19 × 0.14 × 0.11 mm was selected and mounted on a SuperNova, Dual, Cu at home/near, AtlasS2 diffractometer. The crystal was kept at a steady *T* = 140.00(10) K during data collection. The structure was solved with the ShelXT (Sheldrick, 2015) solution program using dual methods and by using Olex2 1.5 (Dolomanov et al., 2009) as the graphical interface. The model was refined with ShelXL 2019/3 (Sheldrick, 2015) using full matrix least squares minimisation on $|F|^2$.

Structure Quality Indicators

**Reflections:**	d min (CuKα) 2Θ=145.3° 0.81	I/σ(I) 29.6	Rint m=2.70 3.48%	Full 135.4° 98% to 145.3° 99.9
**Refinement:**	Shift 0.003	Max Peak 1.1	Min Peak -1.3	GooF 1.048

A clear light-orange irregular-shaped crystal with dimensions 0.19 × 0.14 × 0.11 mm was mounted. Data were collected using a SuperNova, Dual, Cu at home/near, AtlasS2 diffractometer operating at *T* = 140.00(10) K.

Data were measured using ω scans with Cu K_{α} radiation. The diffraction pattern was indexed and the total number of runs and images was based on the strategy calculation from the program

CrysAlis^{Pro} system (CCD 44.119a 64-bit (release 12-08-2025)). The maximum resolution achieved was $\Theta = 72.656^{\circ}$ (0.81 Å).

The unit cell was refined using CrysAlis^{Pro} on 15159 reflections, 66% of the observed reflections.

Data reduction, scaling and absorption corrections were performed using CrysAlis^{Pro}. The final completeness is 99.90 % out to 72.656° in Θ. An analytical absorption correction was performed using CrysAlis^{Pro} 1.171.44.118a (Rigaku Oxford Diffraction, 2025). The analytical numeric absorption correction was done using a multifaceted crystal model based on expressions derived by R.C. Clark & J.S. Reid. (Clark, R. C. & Reid, J. S. (1995). Acta Cryst. A51, 887-897). The empirical absorption correction was done using spherical harmonics, implemented in SCALE3 ABSPACK scaling algorithm. The absorption coefficient μ of this crystal is 6.988 mm^{-1} at this wavelength (λ = 1.54184Å) and the minimum and maximum transmissions are 0.473 and 0.614.

The structure was solved in the space group $P2_1/c$ (# 14) by ShelXT (Sheldrick, 2015) using dual methods. It was refined by full matrix least squares minimisation on $|F|^2$ using version 2019/3 of ShelXL 2019/3 (Sheldrick, 2015). All non-hydrogen atoms were refined anisotropically.

Hydrogen atom positions were calculated geometrically and refined using the riding model.

There is a single formula unit in the asymmetric unit, which is represented by the reported sum formula. In other words: Z is 4 and Z' is 1. The moiety formula is $C_{41}H_{46}AuClN_2$, $2(C_6H_6)$.

Table S10. Crystal data and structure refinement for complex **14**.

**Compound**	**14**
Formula	$C_{53}H_{58}AuClN_2$
$D_{calc.}$/ g cm^{-3}	1.416
μ/mm^{-1}	6.988
Formula Weight	955.43
Colour	clear light orange
Shape	irregular-shaped
Size/mm	0.19×0.14×0.11
T/K	140.00(10)
Crystal System	monoclinic
Space Group	$P2_1/c$
a/Å	16.38797(14)
b/Å	16.08117(10)
c/Å	18.44628(16)
α/°	90
β/°	112.8081(10)
γ/°	90
V/Å^3	4481.18(7)
Z	4
Z'	1
Wavelength/Å	1.54184
Radiation type	Cu K_α
Θ_{min}/°	2.925
Θ_{max}/°	72.656
Index range h	$-20 \le h \le 19$
Index range k	$-19 \le k \le 6$
Index range l	$-19 \le l \le 22$
Measured Refl's.	22928
Indep't Refl's	8674
Refl's I≥2σ(I)	8067
R_{int}	0.0348
Parameters	523
Restraints	0
Largest Peak/eÅ^3	1.126
Deepest Hole/eÅ^3	-1.307
GooF	1.048
R_1 (I≥2σ(I) / all)	0.0276 / 0.0299
wR_2 (I≥2σ(I) / all)	0.0711 / 0.0729
CCDC number	2516495

5. Quantum Chemical Calculations

The solid-state structures of compounds **3** and **6** were used as starting points for geometry optimization. Optimization was performed at the TPSSh/def2-TZVP level of theory[9,10] in implicit *n*-hexane treated by the SMD solvation model[11] using software Gaussian 16.[12] The optimized stationary points were confirmed to be minima by a frequency calculation (NImag = 0).

The calculated Kohn-Sham orbitals were visualized using software Avogadro 1.2.0.[13]

Natural population analysis (NPA) charges and Wiberg bond indices (WBI) were calculated using package NBO 7.0[14] at the same level of theory in the gas phase.

Software Multiwfn 3.8[15,16] was used for the ELF[17] basin analysis, and the selected basins were visualized using software UCSF ChimeraX.[18]

Table S11. Comparison of selected structural parameters between computed and experimental structures of allenes **3** and **6**.

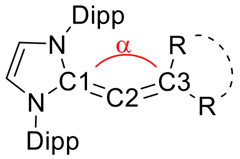

Allene	$\alpha_{(C1–C2–C3)}$ (°)	$d_{(C1–C2)}$ (Å)	$d_{(C2–C3)}$ (Å)
**3** exp.*	138.0(1)	1.371(2)	1.331(2)
**3** opt.**	147.7	1.359	1.330
**6** exp.	172.0(5)	1.313(6)	1.326(6)
**6** opt.	175.7	1.328	1.324

*exp.: measured by XRD.

**opt.: optimized at TPSSh/def2-TZVP level in *n*-hexane (SMD).

	Front view	***Side view***
**3** HOMO	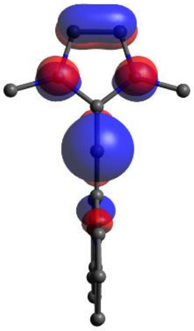	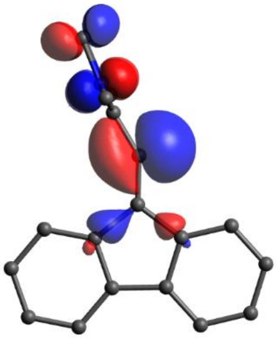
**3** HOMO–2	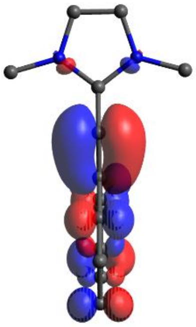	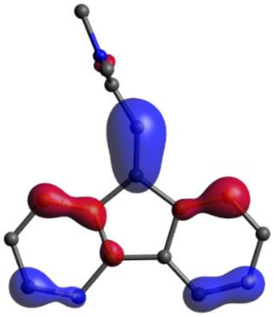
**6** HOMO	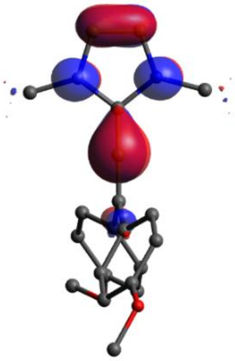	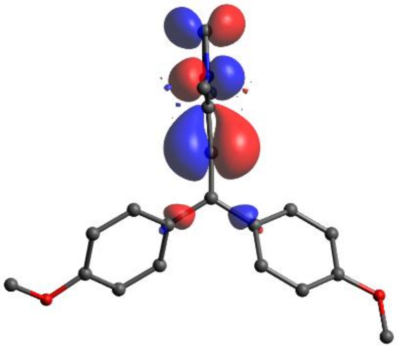
**6** HOMO–1	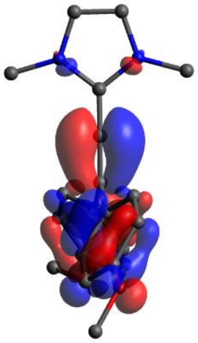	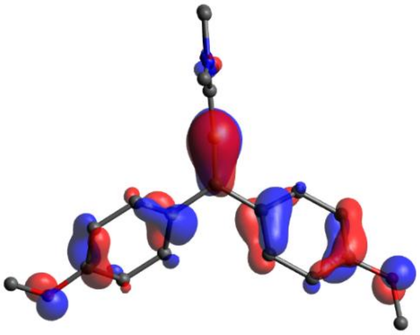

Figure S89. Selected Kohn-Sham orbitals of **3** and **6** (iso = 0.05). Hydrogen atoms are omitted for clarity.

Table S12. Computed NPA charges and Wiberg bond indices of allenes **3** and **6**.

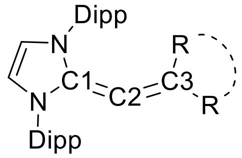

Allene	$\delta_{(C1)}$ (e⁻)	$\delta_{(C2)}$ (e⁻)	$\delta_{(C3)}$ (e⁻)	$WBI_{(C1–C2)}$	$WBI_{(C2–C3)}$	$WBI_{(totalC2)}$
**3**	+ 0.3088	– 0.1470	– 0.0679	1.535	1.770	3.860
**6**	+ 0.2788	– 0.1430	– 0.0335	1.656	1.799	3.951

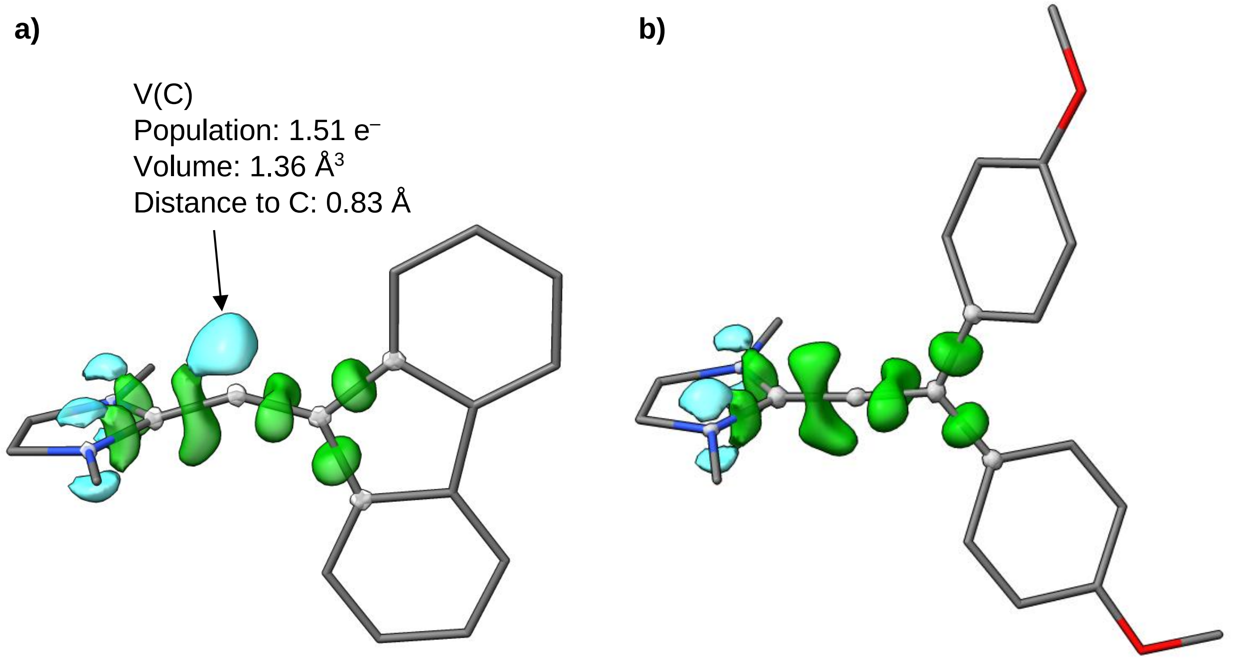

Figure S90. ELF isosurfaces (η = 0.82) of **3** (**a**) and **6** (**b**) computed at the TPSSh/def-2TZVP level of theory with the SMD solvation model (*n*-hexane). Hydrogens and Dipp groups are omitted for clarity. Only basins around the central allene units are shown. Color coding: core basins – white, monosynaptic valence basins – cyan, disynaptic valence basins – green. Image rendered using UCSF ChimeraX.

To investigate the preference of allene **3** for a bent geometry and allene **6** for a linear geometry, we performed a relaxed scan with respect to the bending angle at the TPSSh/def2-SVP level of theory in implicit *n*-hexane treated by the SMD solvation model.

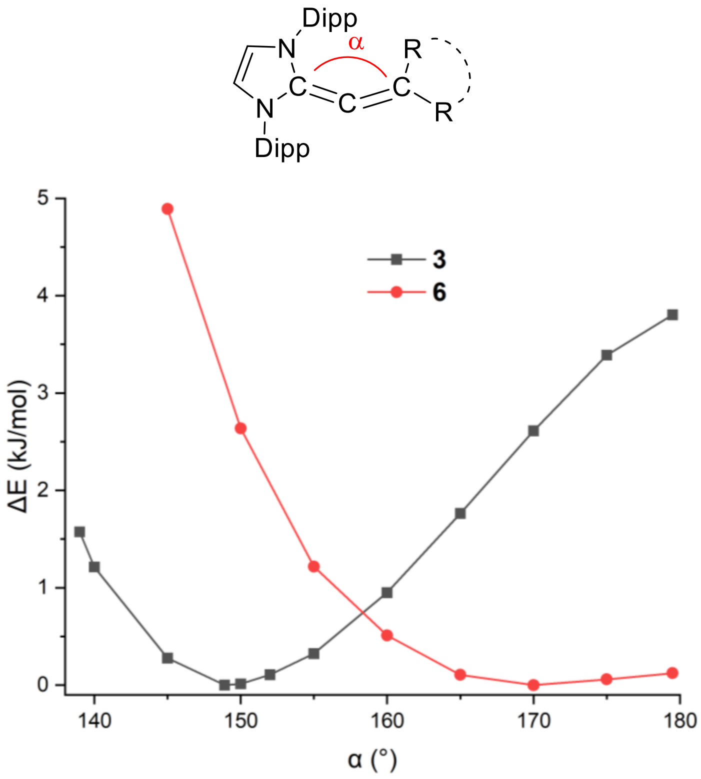

Figure S91. Relative electronic energies of compounds **3** and **6** with respect to the allene bending angle (α). Calculated at the TPSSh/def2-SVP(SMD, *n*-hexane) level. For each allene, the energy of the global minimum was chosen as 0.

The excitation energies of compound **3** were calculated by TD-DFT at the CAM-B3LYP/aug-cc-pVDZ level of theory[19,20] in *n*-hexane (SMD), based on the TPSSh/def2-TZVP (SMD, *n*-hexane) structure.

Table S13. Computed excitation energies (Δ*E*), wavelengths (*λ*), oscillator strengths (*f*), and main orbital contributions of allene **3**, calculated at the TD-CAM-B3LYP/aug-cc-pVTZ(SMD, *n*-hexane)//TPSSh/def2-TZVP(SMD, *n*-hexane) level. Only the first five excitations are shown.

Excitation	Δ*E* (eV)	*λ* (nm)	*f*	main contributions (>2%)
1	2.474	501.2	0.0013	HOMO->LUMO (45%)
2	4.0441	306.6	0.0157	HOMO-1 -> LUMO (48%)
3	4.1399	299.5	0.2483	HOMO->LUMO+7 (17%), HOMO-2->LUMO (9%), HOMO->LUMO+10 (9%)
4	4.2173	293.4	0.002	HOMO->LUMO+3 (47%)
5	4.3308	286.3	0.4564	HOMO-2->LUMO (28%), HOMO->LUMO+1 (5%), HOMO->LUMO+7 (3%)

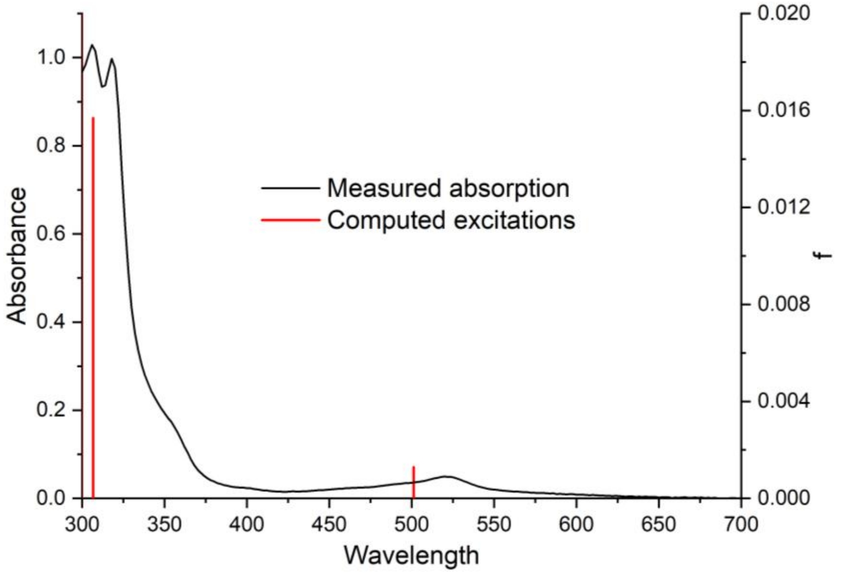

Figure S92. Overlay of measured UV-vis absorption of **3** (5.9 x 10^{-5} M, THF, black) and TD-DFT-calculated transitions at the CAM-B3LYP/aug-cc-pVTZ(SMD, *n*-hexane)//TPSSh/def2-TZVP (SMD, *n*-hexane) level (red sticks, oscillator strengths scaled for clarity).

6. References

1. P. Varava, Z. Dong, R. Scopelliti, F. Fadaei-Tirani, K. Severin, *Nat. Chem.* **2021**, *13*, 1055–1060.
2. D. E. Bergbreiter, J. M. Killough, J*J. Am. Chem. Soc.* **1978**, *100*, 2126–2134.
3. G. B. Wijeratne, E. M. Zolnhofer, S. Fortier, L. N. Grant, P. J. Caroll, C.-H. Chen, K. Meyer, J. Krzystek, A. Ozarowski, T. A. Jackson, D. J. Mindiola, J. Telser, *Inorg. Chem*. **2015**, *54*, 10380−10397.
4. G. M. Sheldrick, *Acta Cryst.*, *Sect. A* **2015**, *71*, 3–8.
5. O. V. Dolomanov, L. J. Bourhis, R. J. Gildea, J. A. K. Howard, H. Puschmann, *J. Appl. Cryst.* **2009**, *42*, 339–341.
6. G. M. Sheldrick, *Acta Cryst.*, *Sect. C* **2015**, *71*, 3–8.
7. **CrysAlis^{Pro}** Software System, Rigaku Oxford Diffraction, (2022).
9. J. Tao, J. P. Perdew, V. N. Staroverov, G. E. Scuseria, *Phys. Rev. Lett.* **2003**, *91*, 146401.
10. F. Weigend, R. Ahlrichs, *Phys. Chem. Chem. Phys.* **2005**, *7*, 3297–3305.
11. A. V. Marenich, C. J. Cramer, D. G. Truhlar, *J. Phys. Chem. B* **2009**, *113*, 6378–6396.
12. M. J. Frisch, *et al.* Gaussian 16, Revision C.01. Gaussian, Inc., Wallingford, CT, **2016**.
13. M. D. Hanwell, D. E. Curtis, D. C. Lonie, T. Vandermeersch, E. Zurek, G. R. Hutchison, *J. Cheminf.* **2012**, *4*, 17.
14. E. D. Glendening, C. R. Landis, F. Weinhold, *J. Comput. Chem.* **2019**, *40*, 2234–2241.
15. T. Lu, F. Chen, *J. Comput. Chem.* **2012**, *33*, 580–592.
16. T. Lu, *J. Chem. Phys.* **2024**, *161*, 082503.
17. A. D. Becke, K. E. Edgecombe, *J. Chem. Phys.* **1990**, *92*, 5397–5403.
18. E. F. Pettersen, T. D. Goddard, C. C. Huang, E. C. Meng, G. S. Couch, T. I. Croll, J. H. Morris, T. E. Ferrim, *Protein Sci.* **2021**, *30*, 70–82. *(Molecular graphics and analyses performed with UCSF ChimeraX, developed by the Resource for Biocomputing, Visualization, and Informatics at the University of California, San Francisco, with support from National Institutes of Health R01-GM129325 and the Office of Cyber Infrastructure and Computational Biology, National Institute of Allergy and Infectious Diseases.)*
19. T. Yanai, D. P. Tew, N. C. Handy, *Chem. Phys. Lett.* **2004**, *393*, 51–57.
20. T. H. Dunning Jr., *J. Chem. Phys.* **1989**, *90*, 1007–1023.
